# Mutational signature distribution varies with DNA replication timing and strand asymmetry

**DOI:** 10.1186/s13059-018-1509-y

**Published:** 2018-09-10

**Authors:** Marketa Tomkova, Jakub Tomek, Skirmantas Kriaucionis, Benjamin Schuster-Böckler

**Affiliations:** 1 Ludwig Cancer Research Oxford, University of Oxford, Old Road Campus Research Building, Oxford, OX3 7DQ UK; 2 Department of Physiology, Anatomy and Genetics, University of Oxford, Oxford, OX1 3PT UK

**Keywords:** Mutagenesis, DNA replication, DNA repair

## Abstract

**Background:**

DNA replication plays an important role in mutagenesis, yet little is known about how it interacts with other mutagenic processes. Here, we use somatic mutation signatures—each representing a mutagenic process—derived from 3056 patients spanning 19 cancer types to quantify the strand asymmetry of mutational signatures around replication origins and between early and late replicating regions.

**Results:**

We observe that most of the detected mutational signatures are significantly correlated with the timing or direction of DNA replication. The properties of these associations are distinct for different signatures and shed new light on several mutagenic processes. For example, our results suggest that oxidative damage to the nucleotide pool substantially contributes to the mutational landscape of esophageal adenocarcinoma.

**Conclusions:**

Together, our results indicate an interaction between DNA replication, the associated damage repair, and most mutagenic processes.

**Electronic supplementary material:**

The online version of this article (10.1186/s13059-018-1509-y) contains supplementary material, which is available to authorized users.

## Background

Understanding the mechanisms of mutagenesis in cancer is important for the prevention and treatment of the disease [1, 2]. Mounting evidence suggests replication itself contributes to cancer risk [3]. Copying of DNA is intrinsically asymmetrical, with leading and lagging strands being processed by distinct sets of enzymes [4], and different genomic regions replicating at defined times during S phase [5]. Previous analyses have focused either on the genome-wide distribution of mutation rate or on the strand specificity of individual base changes. These studies revealed that the average mutation frequency is increased in late-replicating regions [6, 7], and that the asymmetric synthesis of DNA during replication leads to strand-specific frequencies of base changes [8–11]. However, the extent to which DNA replication influences distinct mutational mechanisms, with their manifold possible causes, remains incompletely understood.

Mutational signatures have been established as a powerful approach to quantify the presence of distinct mutational mechanisms in cancer [12]. A mutational signature is a unique combination of the frequencies of all base-pair mutation types (C:G > A:T, T:A > G:C, etc.) and their flanking nucleotides. Since it is usually not known which base in a pair was the source of a mutation, the convention is to annotate mutations from the pyrimidine (C > A, T > A, etc.), leading to 96 possible combinations of mutation types and neighboring bases. Non-negative matrix factorization is used to extract mutational signatures from somatic mutations in cancer samples [12]. This approach has the important advantage of being able to distinguish between processes that have the same major mutation type (such as C > T transitions) but differ in their sequence context. We built upon this feature of mutational signatures and developed a computational framework to identify the replication-strand-specific impact of distinct mutational processes. Using this system, we quantified the replication strand and timing bias of mutational signatures across 19 cancer types. We show that replication affects the distribution of nearly all mutational signatures across the genome, including those that represent chemical mutagens. The unique strand asymmetry and replication timing profile of different signatures reveal novel aspects of the underlying mechanism. For example, we discovered a strong lagging strand bias of T > G mutations in esophageal adenocarcinoma, suggesting an involvement of oxidative damage to the nucleotide pool in the etiology of the disease. Together, our results highlight the critical role of DNA replication and the associated repair in the accumulation of somatic mutations.

## Results and discussion

### Replication bias of mutational signatures

**Fig. 1:**
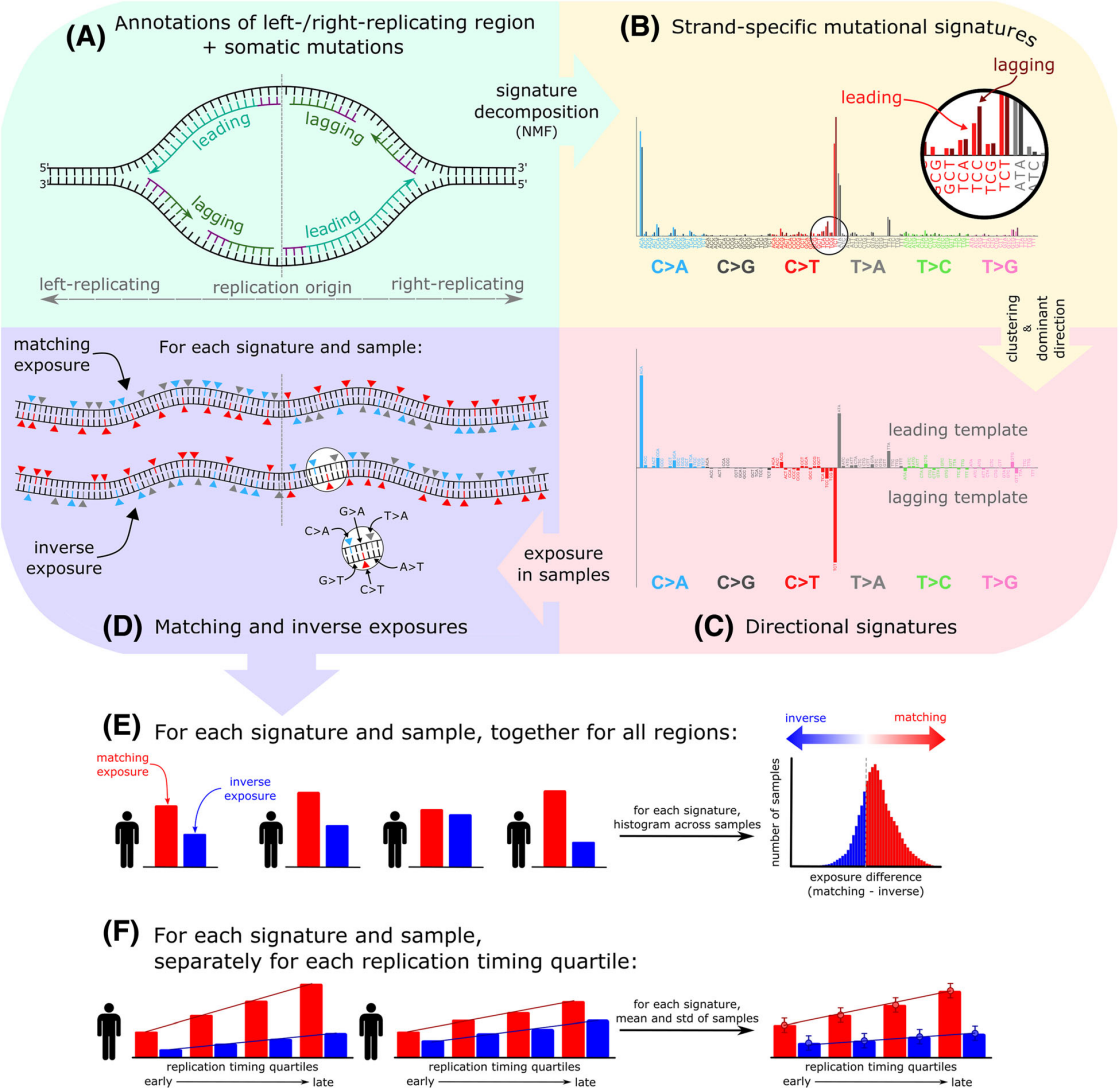
Methods overview. **a** Mutation frequency on the leading and lagging strands is computed using annotated left/right-replicating regions and somatic single-nucleotide mutations oriented according to the strand of the pyrimidine in the base pair. **b** Leading and lagging strand-specific mutational signatures are extracted using non-negative matrix factorization. **c** The signatures are clustered and in each cluster a representative signature is selected (“Methods”). In the cluster representatives, each of the 96 mutation types is annotated according to its dominant direction (*upwards-facing bars* for leading, *downwards-facing bars* for lagging template preference). **d** Exposures to the directional signatures are separately quantified for the leading and lagging strands of each patient. The exposure in the *matching orientation* reflects the extent to which mutations in pyrimidines on the leading (and lagging) strand can be explained by the leading (and lagging) component of the signature, respectively. Conversely, the exposure in the *inverse orientation* reflects how mutations in pyrimidines on the leading strand can be explained by the lagging component of the signature (or vice versa) (“Methods”). Top part of **d** shows an example of a sample with completely matching exposure, given the signature in **c**, with C > T mutations on the leading template and C > A and T > C mutations on the lagging template, whereas the bottom part of **d** shows an example of a sample with completely inverse exposure. **e** Example of matching and inverse exposure quantification in individual patients (for a given signature). Significance of the asymmetry of this signature across the cohort is evaluated based on the distribution of difference between the matching and inverse exposures. The histogram shows an example of a signature with significant matching asymmetry. **f** Signature exposures are next quantified in bins representing four quartiles of replication timing. The graph on the *right* shows average and standard deviation values in individual quartiles, representing an example of a signature enriched in the late-replicated regions both in the matching and inverse exposures

DNA replication in eukaryotic cells is initiated around replication origins (ORI), from where it proceeds in both directions, synthesizing the leading strand continuously and the lagging strand discontinuously (Fig. 1a). We used two independent data sets to describe replication direction relative to the reference sequence, one derived from high-resolution replication timing data [11] and the other from direct detection of ORIs by short nascent strand sequencing (SNS-seq) [13], corrected for technical artifacts [14] (see “Methods”). The former provides information for more genomic loci, while the latter is of higher resolution. As a third measure of DNA replication, we compared regions replicating early during S phase to regions replicating late [11]. We calculated *strand-specific* signatures [15] that add strand information to each mutation type, based on the direction of DNA replication [11] (Fig. 1b). We clustered the strand-specific signatures and further condensed them into *directional signatures* consisting of 96 mutation types, each assigned either “leading” or “lagging” direction depending on the frequency in the strand-specific signature (Fig. 1c; see “Methods”). These directional signatures can be used to separately compute the presence of the signature on the leading and lagging strands in individual samples, which is analogous to what is called the *exposure* to the signature in a sample [16], representing the genome-scale normalized contribution of mutations to the signature (Fig. 1d). Depending on whether the strand bias matches the consensus of the directional signature, the exposure can be *matching* or *inverse.* The latter can occur if the strand bias of a signature in a subset of samples does not match the bias observed in the samples that most strongly contributed to the definition of the signature. We applied this novel algorithm to somatic mutations detected in whole-genome sequencing of 3056 tumor samples from 19 cancer types (Additional file 1: Table S1). We excluded protein-coding genes from the analysis in order to prevent potential confounding of the results by transcription strand asymmetry [11, 12] or selection. Samples with microsatellite instability (MSI) and POLE mutations were treated as separate groups, since they are associated with specific mutational processes. In total, we detected 25 mutational signatures that each corresponded to one of the COSMIC signatures (http://cancer.sanger.ac.uk/cosmic/signatures) and four novel signatures, which were primarily found in samples that had not been previously used for signature extraction (myeloid blood, skin, MSI, and ovarian cancers; Additional file 2: Figures S1–S5).

**Fig. 2:**
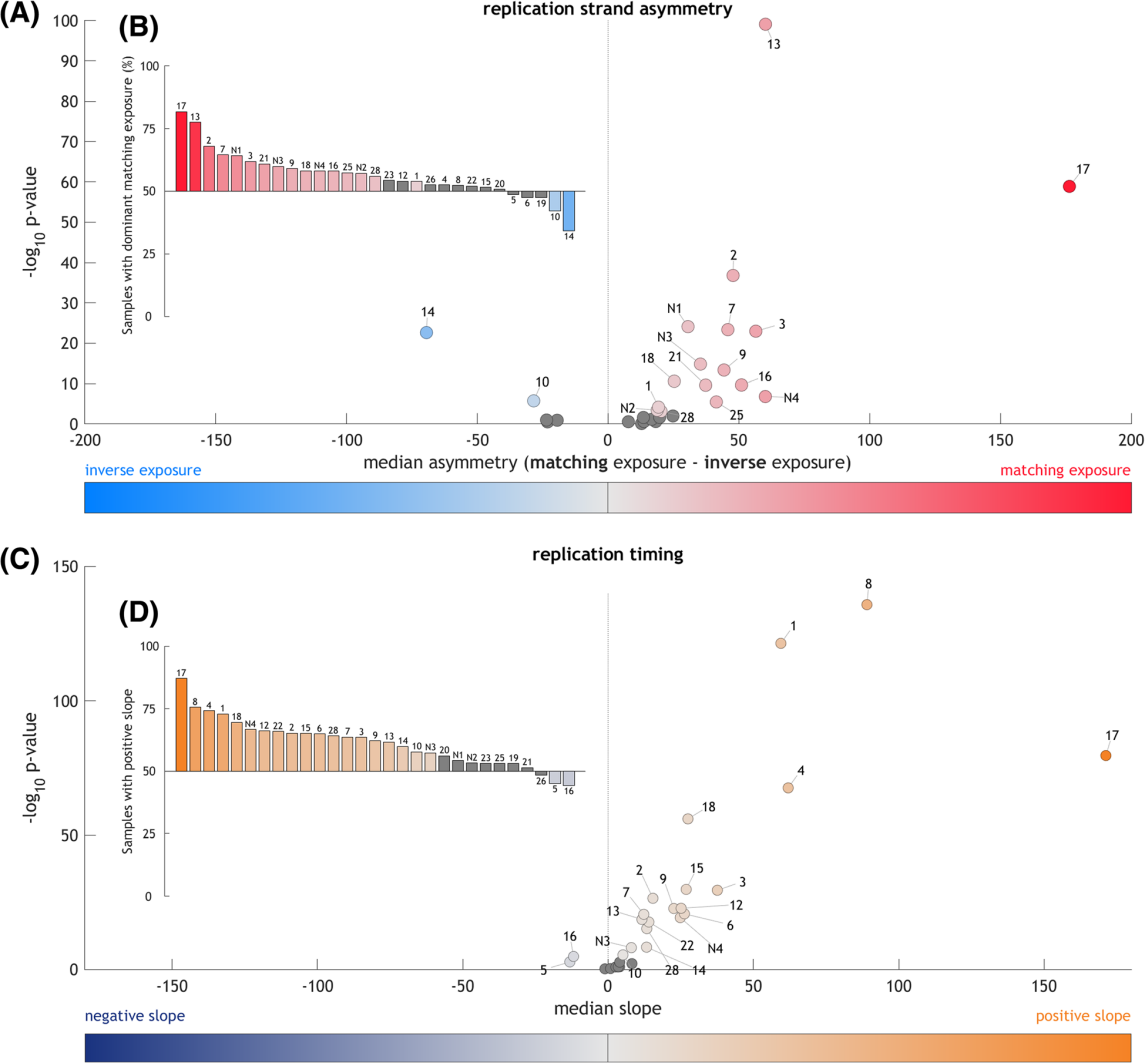
Most mutational signatures exhibit a significant replication strand asymmetry and/or correlation with replication timing. **a** The difference of matching and inverse exposure is computed for each sample and signature. For each signature, the median value of these differences (in samples exposed to this signature) is plotted against -log_10_ q-value (signtest of strand asymmetry per sample; with Benjamini-Hochberg correction). **b** Percentage of samples that have higher matching than inverse exposure to the signature denoted above/below each bar. **c** Correlation of exposures with replication timing. The 20-kbp replication domains were divided into four quartiles by their average replication timing (early-replicated in the first quartile, late-replicated in the last quartile) and exposures to signatures were computed in each quartile. Median slope of correlation with the replication timing is plotted on the x-axis, i.e., values on the *right* denote more mutations in late-replicated regions, values on the *left* reflect more mutations in early replicating regions. The y-axis represents significance of the correlation of signature with replication timing in individual samples (signtest of correlation slope per sample; with Benjamini-Hochberg correction). **d** Percentage of samples with a positive correlation of replication timing with exposure to the signature denoted above/below each bar

In total, 21 out of 29 signatures exhibited significant replication strand asymmetry, 23 were significantly correlated with replication timing, and 27 were significant in at least one of these two metrics (signtest *p* < 0.05, with Benjamini-Hochberg correction; Fig. 2, Additional file 2: Figures S7–S8, S13–S24, Additional file 3: Table S2, Additional file 4: Table S3). Such widespread replication bias across the mutational landscape is surprising, considering that previous reports documented strand bias for only a few mutational processes, such as activity of the APOBEC class of enzymes that selectively edit exposed single-stranded cytosines on the lagging strand [11, 15, 17–19]. Including protein coding genes did not qualitatively change the results (Additional file 2: Figure S9a–c), nor did the exclusion of non-coding in addition to protein-coding genes (Additional file 2: Figure S9d–f). Similarly, using SNS-seq data to determine replication strand direction leads to highly similar findings (Additional file 2: Figure S9g–i). Furthermore, we validated that the strand asymmetry and correlation with replication timing is lost when using random genomic loci as replication origins or replication timing domains (Additional file 2: Figures S10 and S11).

**Fig. 3:**
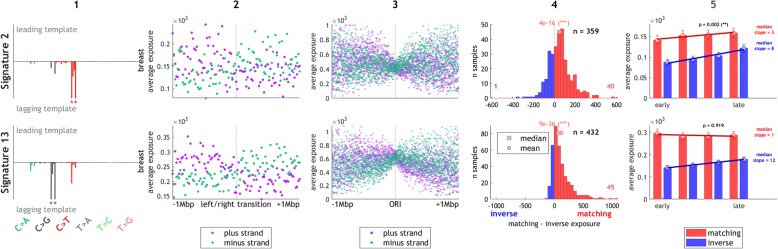
APOBEC signatures in breast cancers show strong but distinct effects of replication. *Column 1*: directional signatures for the two APOBEC signatures, showing proportional contributions of individual mutation types (the absolute values sum to one). A maximum absolute value of 0.2 is shown and mutation types exceeding 0.2 are denoted by an *asterisk*. *Column 2*: mean signature exposure on the plus (Watson) and minus (Crick) strand around transitions between left- and right-replicating regions. The transition corresponds to a region enriched for replication origins. The bin size is 20 kbp. *Column 3*: mean signature exposure on the plus and minus strand around directly ascertained replication origins by SNS-seq, with a bin size of 1 kbp. *Column 4*: distribution of differences between matching and inverse exposure amongst patients with sufficient exposure. Number of outliers is denoted by the small numbers on the sides. *Column 5*: mean matching and inverse exposure in four quartiles of replication timing (*p* value is computed as signtest of slopes of correlation with replication timing in individual samples; the median values of the slope in the matching and inverse directions are shown on the right to the bars). The error bars represent standard error of the mean. The leading and lagging strand annotations used in *columns 2*, *4*, and *5* are based on the direction of replication derived from replication timing data. Plots in *columns 2–5* are based on samples not defined as outliers (by Tukey fences method with *k* = 2)

Our observations confirm that both APOBEC signatures (2 and 13) exhibit clear strand asymmetry, with signature 13 being the most significantly asymmetric signature (q-value 2e^− 98^). In breast cancer samples, we observed differences in these signatures with respect to replication timing: signature 2, but not signature 13, shows a significant enrichment in late replicating regions (Fig. 3), which is consistent with previous reports [15]. These results validate that our approach is able to correctly identify strand and timing asymmetries of mutagenic processes. Consequently, we next tried to interpret the replication biases we observed in other mutational signatures.

Interestingly, the lack of correlation with replication timing in signature 13 seems to be specific to breast cancers, as other cancer types (such as lung squamous, esophageal adenocarcinoma, and pancreas) show a significant correlation (Additional file 2: Figure S12). Nevertheless, the replication strand asymmetry of both signatures 2 and 13 is significantly present in all these tissues.

### Processes directly involving DNA replication or repair

**Fig. 4:**
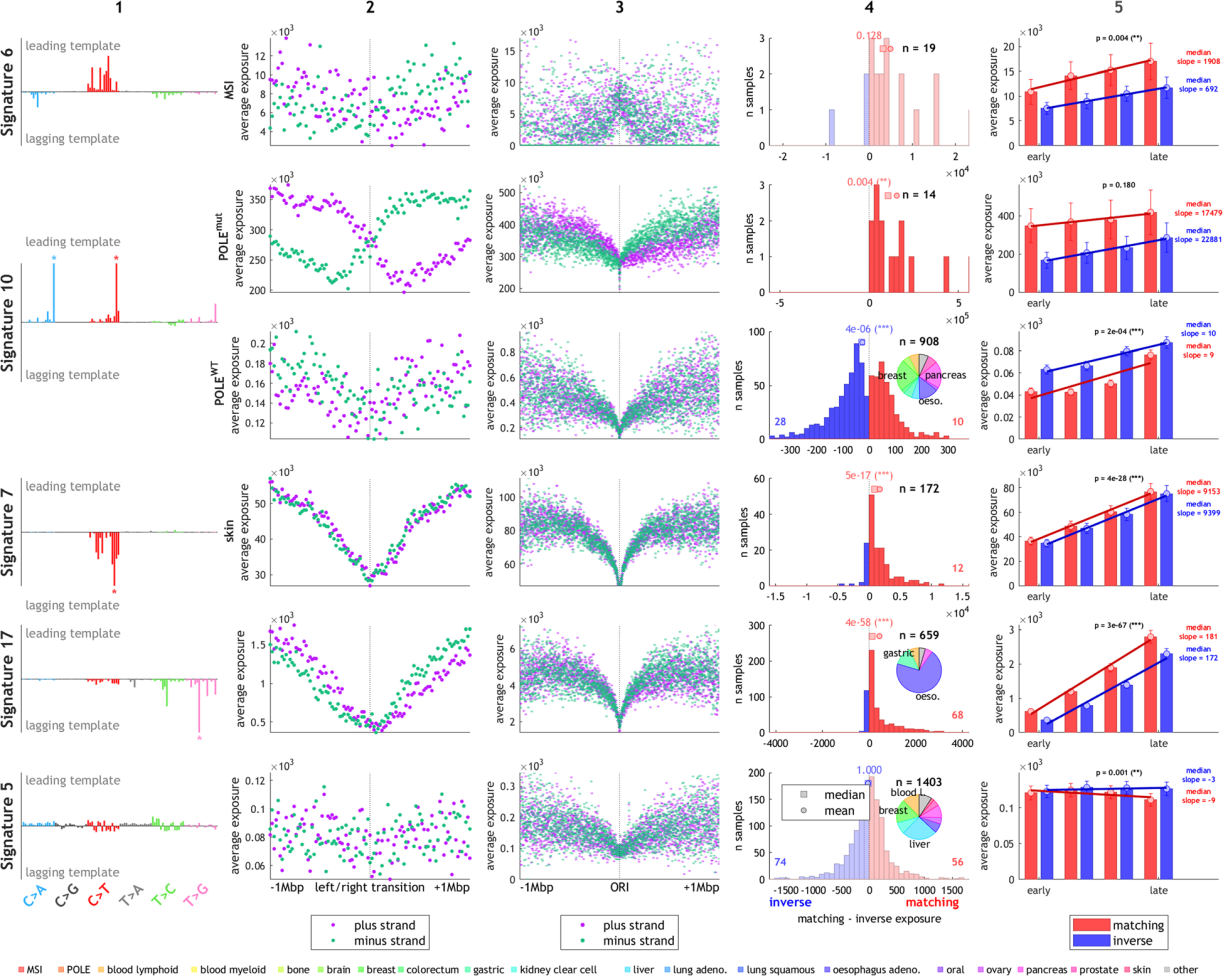
Different mutational signatures exhibit characteristic timing and strand asymmetry profiles. Columns show directional signature (*column 1*), distribution around timing transition regions (*column 2*), and around replication origins (*column 3*), per-patient mutation strand asymmetry (*column 4*; non-significant asymmetry is shown in light-colored histogram), and correlation with replication timing (*column 5*), as described in Fig. 3. The pie chart shows the proportional contributions by individual tissue types (number of samples weighted by their exposure) and the colors of the tissues are explain in the legend at the *bottom*. *Row 1*: signature 6, associated with mismatch-repair deficiency. *Rows 2–3*: signature 10, associated with POLE errors, shown for patients with known POLE mutations (*row 2*), and those without (*row 3*). *Row 4*: signature 7, representing UV-induced damage. *Row 5*: signature 17, characteristic of gastric and esophageal cancers. *Row 6*: signature 5, of unknown etiology, is not discernibly affected by replication

Amongst the better understood mutational mechanisms, several involve replicative processes and DNA repair, such as mismatch-repair deficiency (MMR) [20] or mutations in the proofreading domain of Pol ε (“POLE-MUT samples”) [8, 21]. We first analyzed the signatures representing these mechanisms, since they can be directly attributed to a known molecular process. All five signatures previously associated with MMR and the novel MSI-linked signature N4 exhibit a clear trend of replication strand asymmetry (significant in signature (sig.) 6 and N4 in MSI), generally with enrichment of C > T mutations on the leading strand template and C > A and T > C mutations on the lagging strand template (Fig. 4, Additional file 2: Figure S13), in line with the previously suggested role of MMR to balance mutational asymmetries generated by DNA polymerases during replication [9, 11].

It has previously been proposed that the correlation of overall mutation rate with replication timing (as shown in Fig. 2b) is a direct result of the activity of MMR [22]. In contrast, when splitting the signal by mutational signatures, we observed a more complex relationship. Some MMR signatures in MMR-deficient patients do not significantly correlate with replication timing (sig. 15, 21, 26) or do so only in one direction of replication (such as a negative correlation in the leading direction in sig. 20), whereas others show a clear increase in the late replicated regions (sig. 6 and N4; Additional file 2: Figure S13). We next explored the effect of MMR on correlation with replication timing (irrespective of the replication strand) in all signatures with exposure > 10 in at least four MSI samples. Four signatures exhibited a significant correlation with replication timing in MSI samples (a positive correlation in sig. 6, 18, and N4; negative correlation in sig. 20; Additional file 2: Figure S25). Interestingly, in all these four signatures, the slope of the correlation was significantly steeper in MSI than MSS samples (Additional file 2: Figure S26). Furthermore, some of the other signatures significantly correlated with replication timing in MSS (e.g., sig. 17 and 8) also showed a weak but consistent correlation in MSI. In other signatures (e.g., sig. 1) the correlation is lost. Altogether, these results indicate that MMR is only one of several factors influencing mutagenesis in a timing-dependent manner.

Unexpectedly, two MMR signatures (sig. 6 and N4) showed increased exposures around ORIs (Fig. 4, Additional file 2: Figures S13, S14, S27). Based on experiments in yeast, it has been suggested that MMR is involved in balancing the differences in fidelity of the leading and lagging polymerases [9], in particular repairing errors made by Pol α [9], which primes the leading strand at ORIs and each lagging strand Okazaki fragment [23] and lacks intrinsic proofreading capabilities [24]. It has been recently shown that error-prone Pol α-synthesized DNA is retained in vivo, causing an increase of mutations on the lagging strand [10]. Since regions around ORIs have a higher density of Pol α-synthesized DNA (as discussed, e.g., in [25]), it is possible that increased exposure to signatures 6 and N4 around ORIs is caused by incomplete repair of Pol α-induced errors. The most common Pol α-induced mismatches normally repaired by MMR are G-dT and C-dT, leading to C > T mutations on the leading strand and C > A mutations on the lagging strand [26], matching our observations in the MMR-linked signatures. Notably, we also detected weaker but still significant exposure to MMR signatures in samples with seemingly intact mismatch repair (Additional file 2: Figure S14). Replication strand asymmetry in these samples was substantially smaller, but the higher exposure to signatures 6 and N4 around ORIs remained (Additional file 2: Figure S27). These findings are compatible with a model in which one of the functions of mismatch repair is to balance the effect of mis-incorporation of nucleotides by Pol α. Signatures 6, N4, and possibly 26 appear to reflect this mechanism, while the other MMR signatures might be a result of unrelated functions of MMR, such as its involvement in balancing errors made by other polymerases, e.g., Pol δ.

POLE-MUT samples were previously reported to be “ultra-hypermutated” with excessive C > A and C > T mutations on the leading strand [8, 11, 21]. Mutational signature 10 has been associated with mutations in the proofreading domain of Pol ε, the main leading strand polymerase [23, 27]. We noticed that mutational signature 14 is also strongly associated with POLE mutations in the data sets by Shlien et al. [21], Alexandrov et al. [12], and in The Cancer Genome Atlas (TCGA). Since then, Andrianova et al. confirmed this observation, showing that signature 14 is enriched in POLE-MUT samples with mismatch repair deficiency [28]. As expected, we observed very strong strand asymmetry for these two signatures in all POLE-MUT samples, with an increase of C > A, C > T, and T > G mutations on the leading strand (Fig. 4, Additional file 2: Figure S15). As with MMR signatures, we also found weak but significant evidence of signature 10 and 14 in samples without Pol ε defects (POLE-WT). Strikingly, however, in these samples the strand asymmetry was in the inverse orientation compared to the POLE-MUT samples, i.e., more C > A, C > T, and T > G mutations on the lagging strand (Fig. 4, Additional file 2: Figure S16). Conversely, we detected the presence of two signatures of unknown etiology, signatures 18 and 28, in POLE-MUT samples, but in the inverse orientation compared to POLE-WT samples. We performed two additional analyses in order to validate that this is not an artifact of spurious/wrong associations in the signature exposures decomposition. First, we removed exposures not robust to perturbations (see “Methods”) and confirmed that all four signatures (10, 14, 18, and 28) remained significantly strand asymmetric in both POLE-MUT and POLE-WT samples (Additional file 2: Figures S17–S18). Second, we directly compared the frequencies of the most prominent mutation types for each of the four signatures (sig. 10, 14, 18, and 28) in POLE-MUT and POLE-WT samples on the leading and lagging strands. The inverse strand preference observed in the signatures was also detected for individual mutation types. For example, the frequency of mutations in TCT > A, TCG > T, and TTT > G, the three major components of signature 10, is higher on the lagging strand than on the leading strand in POLE-WT samples, whereas it is higher on the leading strand in POLE-MUT (Additional file 2: Figures S28–S31). We therefore hypothesize that POLE-linked signatures are originally caused by a process that affects both strands and, under normal circumstances, is slightly enriched on the lagging strand. This could be caused by certain types of DNA lesions which under normal circumstances are less accurately replicated when on the template of the lagging strand (e.g., due to a lower fidelity of Pol δ or Pol α compared to wild-type Pol ε when replicating these lesions). In POLE-MUT samples the lack of replication-associated proofreading would then lead to a strong relative increase in these mutations on the leading strand, explaining the flipped orientation of signatures.

Apart from replication strand asymmetry, we also observed a significant correlation with replication timing in signatures 10, 14, 18, and 28 in POLE-WT samples (Additional file 2: Figure S16). The correlation was significant only in signature 28 in POLE-MUT samples, but at least 75% of samples showed a positive slope of the correlation also in signatures 10, 14, and 18, and the slope was significantly increased in POLE-MUT compared to MSS POLE-WT samples in signatures 10, 18, and 28 (Additional file 2: Figures S15, S16, S32, S33). Future studies with larger sample size are needed to evaluate the effect of POLE mutations and MMR on the replication timing characteristics on this group of mutational signatures.

### Signatures linked to environmental mutagens

We next focused on signatures that have not previously been reported to be connected to replication, or for which the causal mechanism is unknown. Our data show a link between DNA replication and exogenous mutagens such as UV light (signature 7), tobacco smoke (signature 4), or aristolochic acid (AA; signature 22) [29]. In these signatures, we observed marked correlation with replication timing (Fig. 4, Additional file 2: Figures S19 and S20). Higher mutation frequency late in replication has been observed in mouse embryonic fibroblast (MEFs) treated with AA or Benzo[a]pyrene (B[a]P; a mutagen in tobacco smoke) [30]. This increased mutagenicity might be attributed to different DNA damage tolerance pathways being active during early and late replication. Regions replicated early in S-phase are thought to prefer high-fidelity template switching, whereas regions replicated late are more likely to require translesion synthesis (TLS), which has a higher error rate [31–37]. This is consistent with the observation in yeast that a disruption of TLS leads to decreased mutation frequency in late-replicating regions and therefore a more even distribution of mutation frequency between early and late-replicating regions [32]. In particular, TLS has been observed to increase in activity and mutagenicity later in the cell cycle when replicating DNA damaged by B[a]P [38]. Alternatively, differences in chromatin accessibility could be responsible for the decreased mutagenicity in early-replicated regions. Open chromatin is, on average, replicated earlier and is also more accessible to repair enzymes, which could contribute to the decreased mutation frequency in early-replicating regions [39].

We also observed weak but significant replication strand asymmetry in the mutagen-induced signatures in the tissues associated with the respective mutagen (Additional file 2: Figure S19). Signature 4 has a significant strand asymmetry in lung cancers, similar to signature 7 in skin cancers. Signature 22 in kidney cancers has a small sample size but shows the same trend. This matches a previously observed lower efficiency of bypass of DNA damage on the lagging strand [40] and strong mutational strand asymmetry in cells lacking Pol η, the main TLS polymerase responsible for the replication of UV-induced photolesions [41]. Altogether, our data highlight the importance of replication in converting DNA damage into actual mutations and suggest that bypass of DNA damage occurring on the lagging template results in detectably lower fidelity on this strand.

Signature 17 had the largest median strand asymmetry (sixth largest log_2_ ratio of the two strands) and also was one of the signatures with the strongest correlations with replication timing (Figs. 2 and 4, Additional file 2: Figure S7). The mutational process causing this signature is unclear. We noted that the timing asymmetry and exposure distribution around ORIs to signature 17 closely resembled that of signatures 4 and 7, suggesting a possible link to DNA damage. Signature 17 is most prominent in gastric cancers and esophageal adenocarcinoma (EAC), where it appears early during disease development [42], and it is also present in Barrett’s esophagus (BE), a precursor to EAC [43]. Due to the importance of gastro-esophageal and duodeno-gastric reflux in the development of BE and EAC [44–46] and the resulting oxidative stress [47–50], it has been speculated that oxidative damage could cause the mutation patterns characteristic for signature 17 [51, 52]. Increased oxidative damage to guanine has been reported in the epithelial cells of dysplastic BE as well as after incubation of BE tissue with a cocktail mimicking bile reflux [50]. Oxidative stress affects bases in not only the DNA but also the nucleotide pool, such as the oxidation of dGTP to 8-oxo-dGTP. This oxidized dGTP derivative has been shown to induce T > G transversions [53–55] through incorporation by TLS polymerases into DNA opposite A on the template strand [56]. In contrast, oxidation of guanine in the DNA produces 8-oxo-G, which has been shown to result in C > A mutations when paired with adenine during replication [57]. These C > A mutations are normally prevented by DNA glycosylases in the base excision repair pathway, such as MUTYH and OGG1, which repair 8-oxo-G:A pairs to G:C. However, if an 8-oxo-G:A mismatch resulted from incorporation of 8-oxo-dGTP in the de novo synthesized strand, the “repair” to G:C would actually lead to a T > G mutation [57]. Consequently, depletion of MUTYH led to an increase of C > A mutations [57, 58] but a decrease of T > G mutations induced by 8-oxo-dGTP [59]. Importantly, the mismatch of 8-oxo-G and A has been shown in yeast to be more efficiently repaired into G:C when 8-oxo-G is on the lagging strand template [60, 61], resulting in an enrichment of T > G mutations on the lagging strand template if the 8-oxoG:A mismatch originated from incorporation of 8-oxo-dGTP opposite A. Our data show strong lagging-strand bias of T > G mutations and overall higher exposure to signature 17 on the lagging strand, supporting the hypothesis that signature 17 is a by-product of oxidative damage.

### DNA methylation-linked mutagenesis

A small but significant strand asymmetry was detected in signature 1 (Additional file 2: Figure S22). This observation is difficult to explain with spontaneous deamination of 5-methylcytosine, the assumed etiology of signature 1. However, the observation would be in line with a previously hypothesized model in which Pol ε has decreased fidelity of replicating 5-methylcytosine, causing an enrichment of C > T mutations in methylated cytosines on the leading strand, especially in samples with deficiency in MMR or Pol ε proofreading [62]. Our analysis shows that signature 1 is slightly enriched on the leading strand, even in samples proficient for post-replicative proofreading and repair. Moreover, we observed that signature 1 is significantly correlated with replication timing in MSS, but not in MSI samples (Additional file 2: Figure S26), in line with the possibility that MMR repairs C > T errors in a CpG context introduced by Pol ε, as MMR is thought to be active primarily in the early-replicated regions [22].

## Conclusions

Our findings demonstrate how the relationship between mutational signatures and DNA replication can help to illuminate the mechanisms underlying several currently unexplained mutational processes, as exemplified by signature 17 in esophageal cancer. Crucially, our computational analysis produces testable hypotheses which we anticipate to be experimentally validated in the future; for instance, that bypass of external-mutagen-induced DNA damage (such as UV light, tobacco smoking, and aristolochic acid) is more error prone during synthesis of the lagging strand, or that oxidative damage to the dNTP pool contributes to the etiology of signature 17. Our results also add a new perspective to the recent debate regarding the correlation of tissue-specific cell division rates with cancer risk [3]. It has been argued that this correlation is primarily attributable to “bad luck” in the form of random errors that are introduced during replication by DNA polymerases. Critics of this theory have pointed out that the range of mutational signatures observed in cancer samples makes a purely replication-driven etiology of cancer mutations unlikely [63, 64]. Our analysis at least partially reconciles the two arguments, showing that most mutational signatures are themselves affected by DNA replication, including signatures linked to environmental mutagens. The presence of mutational signatures on the one hand and a strong relationship between replication and the risk of cancer on the other therefore need not be mutually exclusive. In conclusion, our results provide evidence that DNA replication interacts with most processes that introduce mutations in the genome, suggesting that differences amongst DNA polymerases and post-replicative repair enzymes might play a larger part in the accumulation of mutations than previously appreciated.

## Methods

### Somatic mutations

Cancer somatic mutations in 3056 whole-genome sequencing samples (Additional file 1: Table S1, Additional file 5: Table S4) were obtained from the data portal of TCGA, the data portal of the International Cancer Genome Consortium (ICGC), and previously published data in peer review journals [12, 21, 51, 65, 66]. For TCGA samples, aligned reads of paired tumor and normal samples were downloaded from the Genomic Data Commons Data Portal website under TCGA access request #10140 and somatic variants were called using Strelka (version 1.0.14) [67] with default parameters. The status of POLE-MUT and MSI samples were obtained from the supplementary data of [11, 21, 66].

### Direction of replication

Left- and right-replicating domains were taken from [11] where replication timing profiles were generated in six lymphoblastoid cell lines [68], valleys and peaks (defined as regions with a slope with a magnitude lower than 250 rtu per Mb) were removed, after which left- and right-replicating domains were defined as timing transition regions with a negative and positive slope, respectively [11]. In the left-replicated regions, the reference strand is used as a template for the leading strand, while the opposite strand is used as a template for the lagging strand, and vice versa for the right-replicated regions. Each domain (called territory in the original source code and data) is 20 kbp wide and annotated with the direction of replication and with replication timing.

### Excluded regions

The following regions were excluded: regions with low unique mappability of sequencing reads (positions with mean mappability in 100-bp sliding windows below 0.99 from UCSC mappability track: alignability of 50mers, accession number wgEncodeEH000320), gencode protein coding genes, and blacklisted regions defined by Anshul Kundaje [69] (Anshul_Hg19UltraHighSignalArtifactRegions.bed, Duke_Hg19SignalRepeatArtifactRegions.bed, and wgEncodeHg19ConsensusSignalArtifactRegions.bed from http://mitra.stanford.edu/kundaje/akundaje/release/blacklists/hg19-human/).

### Mutation frequency analysis

All variants were classified by the pyrimidine of the mutated Watson-Crick base pair (C or T), strand of this base pair (C or T), and the immediate 5′ and 3′ sequence context into 96 possible mutation types as described by Alexandrov et al. [12]. The frequency of trinucleotides on each strand was computed for each replication domain. Then the mutation frequency of each mutation type in each replication domain on the leading (plus = Watson strand in left-replicating domains; minus = Crick strand in right-replicating domains) and lagging strand (vice versa) was computed for each sample.

### Extraction of mutational signatures

Matlab code [12] was used for extraction of strand-specific mutational signatures. The input data were the mutation counts on the leading and lagging strands (summed from all replicating domains together, but without the excluded regions) in each sample. The 192-elements-long mutational signatures (example in Fig. 1b) were extracted in each cancer type separately (for *K* number of signatures between 2 and 7). The best *K* with minimal error and maximal stability (minimizing error_*K*_/max(error) + (1 − stability_*K*_) and with stability of at least 0.8) was selected for each cancer type. The stability metric (computed as average silhouette width of clusters of signatures computed by Non-negative Matrix Factorisation) represents reproducibility of the model, while the error (computed as the average Frobenius reconstruction error) evaluates the accuracy with which the deciphered mutational signatures and their respective exposures describe the original matrix of mutations. Signatures present in only a small number of samples with very low exposures were excluded ((95th percentile of exposures of this signature)/(Mean total exposure per samples) < 0.2). The remaining signatures were then normalized by the frequency of trinucleotides in the leading and lagging strands and subsequently multiplied by the frequency of trinucleotides in the genome. This made them comparable with the 30 previously identified whole-genome-based COSMIC signatures (http://cancer.sanger.ac.uk/cosmic/signatures). Signatures extracted in each cancer type and COSMIC signatures were all pooled together (with equal values in the leading and lagging parts in the COSMIC signatures) and were clustered using unsupervised hierarchical clustering (with cosine distance and complete linkage). A threshold of 27 signatures was selected to identify clusters of similar signatures. Mis-clustering was avoided by manual examination (and whenever necessary re-assignment) of all signatures in all clusters (Additional file 2: Figure S6). The resulting 29 signatures (representing the detected clusters) contained 25 previously observed (COSMIC) and four new signatures. For the subsequent analysis, the signatures were converted back to 96 values: the 25 COSMIC signatures were used in their original form (i.e., having the same values as on the COSMIC website) and for the four newly identified signatures we used an average of the leading and lagging parts of the 192-elements-long signatures.

### Annotation of signatures with leading and lagging direction

Each signature was annotated with the dominant strand direction (leading vs lagging) in each of the 96 mutation types (Fig. 1c). This was based on the dominant strand direction within the signature’s cluster. Types with unclear direction and small values were assigned according to the predominant direction of other trinucleotides of the same mutation group, such as C > T.

### Calculating strand-specific exposures in individual samples

Exposures to leading and lagging parts of the signatures on the leading and lagging strands in individual samples were quantified using non-negative least squares regression using the Matlab function *e = lsqnonneg(S, m)*, where:

$$ S=\left(\begin{array}{cc}{S}_{LD}& {S}_{LG}\\ {}{S}_{LG}& {S}_{LD}\end{array}\right),m=\left(\genfrac{}{}{0pt}{}{m_{LD}}{m_{LG}}\right),e=\left(\genfrac{}{}{0pt}{}{e_{matching}}{e_{inverse}}\right). $$

The matrix *S*_*LD*_ has 96 rows and 29 columns and represents the leading parts of the signatures, i.e., the elements of the lagging parts contain zeros in this matrix. Similarly, *S*_*LG*_ has the same size but contains zeros in the leading parts. The vector *m*_*LD*_ of length 96 contains mutations on the leading strand (again normalized by trinucleotides in leading strand/whole genome), and similarly *m*_*LG*_ contains mutations from the lagging strand. Finally, *lsqnonneg* finds a non-negative vector of exposures *e* such that it minimizes a function *|m – S · e|*. A similar approach has been used in [70] for finding exposures to a given set of signatures. Our extension includes the strand-specificity of the signatures. The interpretation of the model is that the *matching exposure e*_*matching*_ represents exposure of the leading part of the signature on the leading strand and exposure of the lagging part of the signature on the lagging strand, whereas *e*_*inverse*_ represents the two remaining options. It is important to note that the direction of the mutation is relative to the nucleotide in the base pair chosen as the reference, i.e., mutations of a pyrimidine on the leading strand correspond to mutations of a purine on the lagging strand. In order to minimize the number of spurious signature exposures, the least exposed signature was incrementally removed (in both leading and lagging parts) as long as the resulting error did not exceed the original error by 0.5%. The resulting reported values in each sample and signature were the difference (or fold change) of *e*_*matching*_ and *e*_*inverse*_. In each signature, the signtest was used to compare matching and inverse exposures across samples with sufficient minimal exposure (at least 10) to the signature. Benjamini-Hochberg correction was applied to correct for multiple testing.

### Replication origins

The left/right transitions of the replication domains represent regions with, on average, higher density of replication origins. In order to get better resolution of the replication origins, and to validate the results using an independent estimates of left- and right-replicating domains, genome-wide maps of human replication origins from SNS-seq by [13] were used. Eight fastq files (HeLa, iPS, hESC, IMR; each with two replicates) were downloaded and mapped to hg19 using bowtie2 (version 2.1.0). To control for the inefficient digestion of λ-exo step of SNS-seq, reads from non-replicating genomic DNA (LexoG0) were used as a control [14]. Peaks were called using “macs callpeak” with parameters --gsize = hs --bw = 200 --qvalue = 0.05 --mfold 5 50 and LexoG0 mapped reads as a control. Only peaks covered in at least seven of the eight samples were used. We generated 1000 1-kbp bins to the left and right of each origin, as long as they did not reach half the distance to the next origin. We then used these replication direction annotations in the 1-kbp bins to calculate strand-specific exposures in individual samples as above and ascertained that both approaches lead to qualitatively very similar mutational strand asymmetries in individual signatures (Additional file 2: Figure S9).

### Quantification of exposures with respect to replication timing, left/right transitions, and replication origins

Replication domains were divided into four quartiles by their average replication timing. The entire exposure quantification was computed separately in each quartile, or bin around left/right transition or bin around replication origin. In replication timing plots, a linear regression model (function fitlm in MatLab) was fitted to the mean exposure in each quartile (separately for matching and inverse exposures) and signtest on slopes of the fits in individual samples was used to evaluate significance of correlation with replication timing across the cohort. Benjamini-Hochberg correction was applied to correct for multiple testing.

### Evaluation of robustness to noise in signature exposures

For each sample, 1000 perturbations of the input mutation frequency vector were performed, adding noise generated from normal distribution with mean 0 and standard deviation as 5% of the original mutation frequency. Exposures to signatures were quantified for each perturbation. Signatures with extremely variable exposures ((qtl75 − x)/x ≥ 0.3 or (x − qtl25)/x) ≥ 0.3) were removed for this sample and then the exposures were re-computed using only the signatures that passed the filtering.

## Additional files

Additional file 1: **Table S1.** Overview of the used whole-genome sequencing samples. (PDF 211 kb)

Additional file 2: **Figures S1–S33.** Supplementary figures. (PDF 6116 kb)

Additional file 3: **Table S2.** Overview of the strand asymmetry and correlation with replication timing in mutational signatures. (PDF 205 kb)

Additional file 4: **Table S3.** Values of data points. (XLSX 12 kb)

Additional file 5: **Table S4.** List of all samples. (XLSX 1426 kb)

Additional file 6: **Table S5.** Values of the strand-specific mutational signatures. (XLSX 51 kb)

Additional file 7: Review history. (DOCX 64 kb)
