# Supplementary material for: Mutational signature distribution varies with DNA replication timing and strand asymmetry
Source: Genome Biol. 2018 Sep 10;19:129. doi: 10.1186/s13059-018-1509-y (PMC6130095; doi:10.1186/s13059-018-1509-y)
Supplement: Supplementary file 1 — Table S1. Overview of the used whole-genome sequencing samples. (PDF 211 kb) [file 13059_2018_1509_MOESM1_ESM.pdf]

Table S1: Overview of the used whole genome sequencing samples.

Cohort	Cancer type	samples	Source
**Alexandrov_Ding_AML**	Blood myeloid	7	(Alexandrov et al. 2013) Publicly available at ftp://ftp.sanger.ac.uk/pub/cancer/AlexandrovEtAl/
**Alexandrov_Imielinski_Lung_Adeno**	Lung adenocarcinoma	24	(Alexandrov et al. 2013) Publicly available at ftp://ftp.sanger.ac.uk/pub/cancer/AlexandrovEtAl/
**Alexandrov_Lymphoma_B_cell**	Blood lymphoid	24	(Alexandrov et al. 2013) Publicly available at ftp://ftp.sanger.ac.uk/pub/cancer/AlexandrovEtAl/
**Bass_Colon**	Colorectum	9	(Bass et al. 2011) Publicly available at http://www.broadinstitute.org/~lawrence/crc/
**bMMRD**	POLE	2	(Shlien et al. 2015) Access provided by the authors
**Dulak_Oesophagus**	Oesophagus adenocarcinoma	16	(Dulak et al. 2013) Publicly available at https://personal.broadinstitute.org/shouyong/eac/
**ICGC_BOCA_FR**	Bone	97	ICGC BOCA-FR

**ICGC_BRCA_EU**	Breast	560	ICGC BRCA-EU
**ICGC_CLLE_ES**	Blood lymphoid	150	ICGC CLLE-ES
**ICGC_COCA_CN**	Colorectum	26	ICGC COCA-CN
**ICGC_EOPC_DE**	Prostate	62	ICGC EOPC-DE
**ICGC_ESAD_UK**	Oesophagus adenocarcinoma	203	ICGC ESAD-UK
**ICGC_LICA_FR**	Liver	14	ICGC LICA-FR
**ICGC_LINC_JP**	Liver	31	ICGC LINC-JP
**ICGC_LIRI_JP**	Liver	258	ICGC LIRI-JP
**ICGC_LUSC_CN**	Lung squamous	4	ICGC LUSC-CN
**ICGC_LUSC_KR**	Lung squamous	30	ICGC LUSC-KR
**ICGC_MALY_DE**	Blood lymphoid	100	ICGC MALY-DE
**ICGC_MELA_AU**	Skin	183	ICGC MELA-AU
**ICGC_ORCA_IN**	Oral	25	ICGC ORCA-IN
**ICGC_OV_AU**	Ovary	93	ICGC OV-AU
**ICGC_PACA_AU**	Pancreas	161	ICGC PACA-AU
**ICGC_PACA_CA**	Pancreas	159	ICGC PACA-CA
**ICGC_PAEN_AU**	Pancreas	48	ICGC PAEN-AU
**ICGC_PAEN_IT**	Pancreas	37	ICGC PAEN-IT
**ICGC_PBCA_DE**	Brain	236	ICGC PBCA-DE
**ICGC_PRAD_CA**	Prostate	124	ICGC PRAD-CA
**ICGC_PRAD_UK**	Prostate	108	ICGC PRAD-UK

**ICGC_RECA_EU**	Kidney clear cell	95	ICGC RECA-EU
**TCGA_AML_Strelka**	Blood myeloid	49	TCGA LAML
**TCGA_MSI_Strelka**	MSI	9	TCGA COAD
**TCGA_POLE_COAD_Strelka**	POLE	7	TCGA COAD
**TCGA_POLE_READ_Strelka**	POLE	3	TCGA READ
**TCGA_POLE_UCEC_Strelka**	POLE	2	TCGA UCEC
**Wang_Gastric_MSI**	MSI	10	(Wang et al. 2014) Publicly available at http://web.hku.hk/~suetyi/.
**Wang_Gastric_MSS**	Gastric	90	(Wang et al. 2014) Publicly available at http://web.hku.hk/~suetyi/.
