# Supplementary material for: Mutational signature distribution varies with DNA replication timing and strand asymmetry
Source: Genome Biol. 2018 Sep 10;19:129. doi: 10.1186/s13059-018-1509-y (PMC6130095; doi:10.1186/s13059-018-1509-y)
Supplement: Supplementary file 2 — Figures S1–S33. Supplementary figures. (PDF 6116 kb) [file 13059_2018_1509_MOESM2_ESM.pdf]

Supplementary Figures for

Mutational signature distribution varies with DNA replication timing and strand asymmetry

Marketa Tomkova, Jakub Tomek, Skirmantas Kriaucionis and Benjamin Schuster-Böckler

correspondence to: benjamin.schuster-boeckler@ludwig.ox.ac.uk

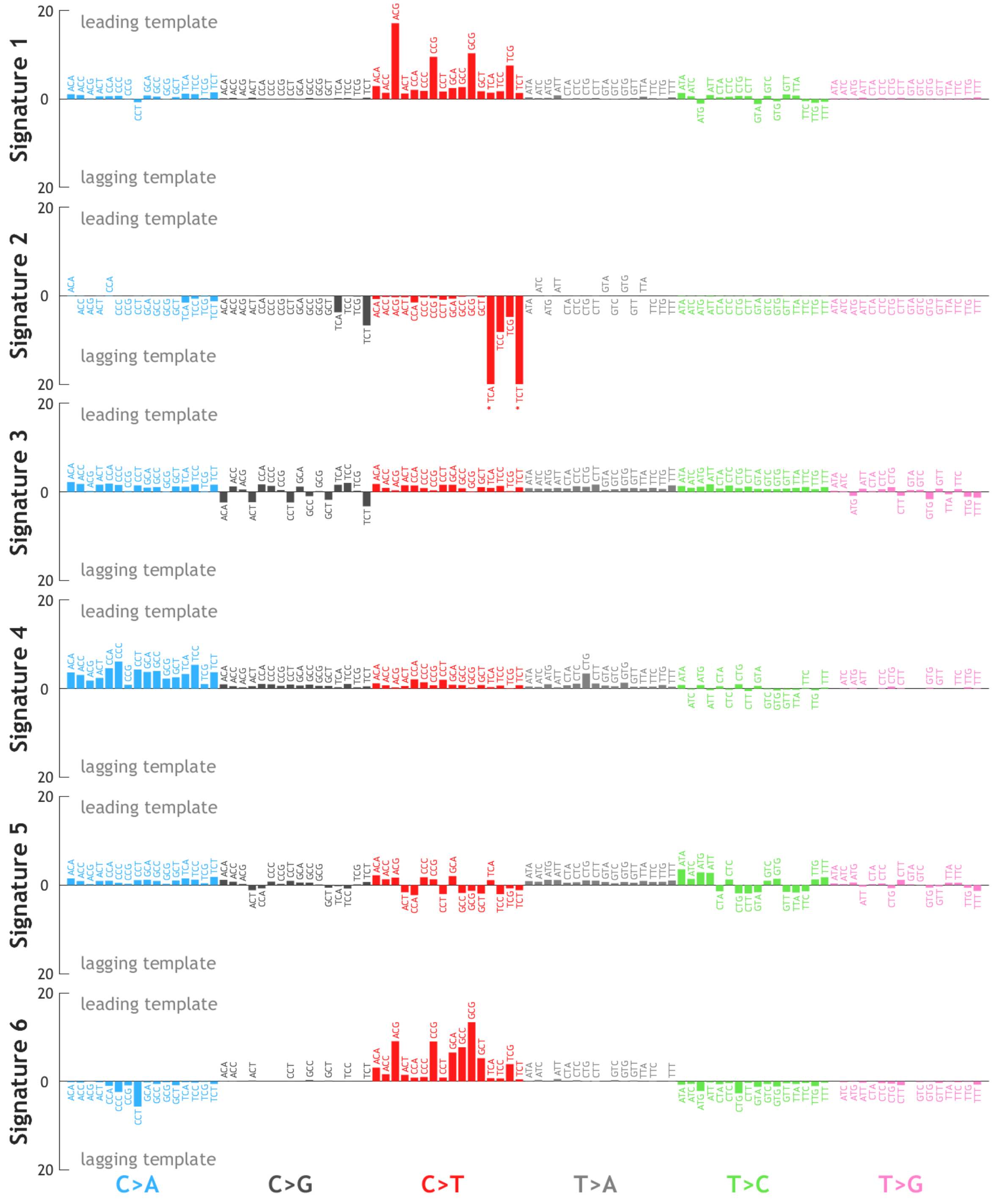

Fig. S1: Directional signatures 1-6. Each of the 96 mutation types is annotated with a dominant direction: leading (pointing up), or lagging (pointing down). Asterisks indicate mutation types exceeding 20%.

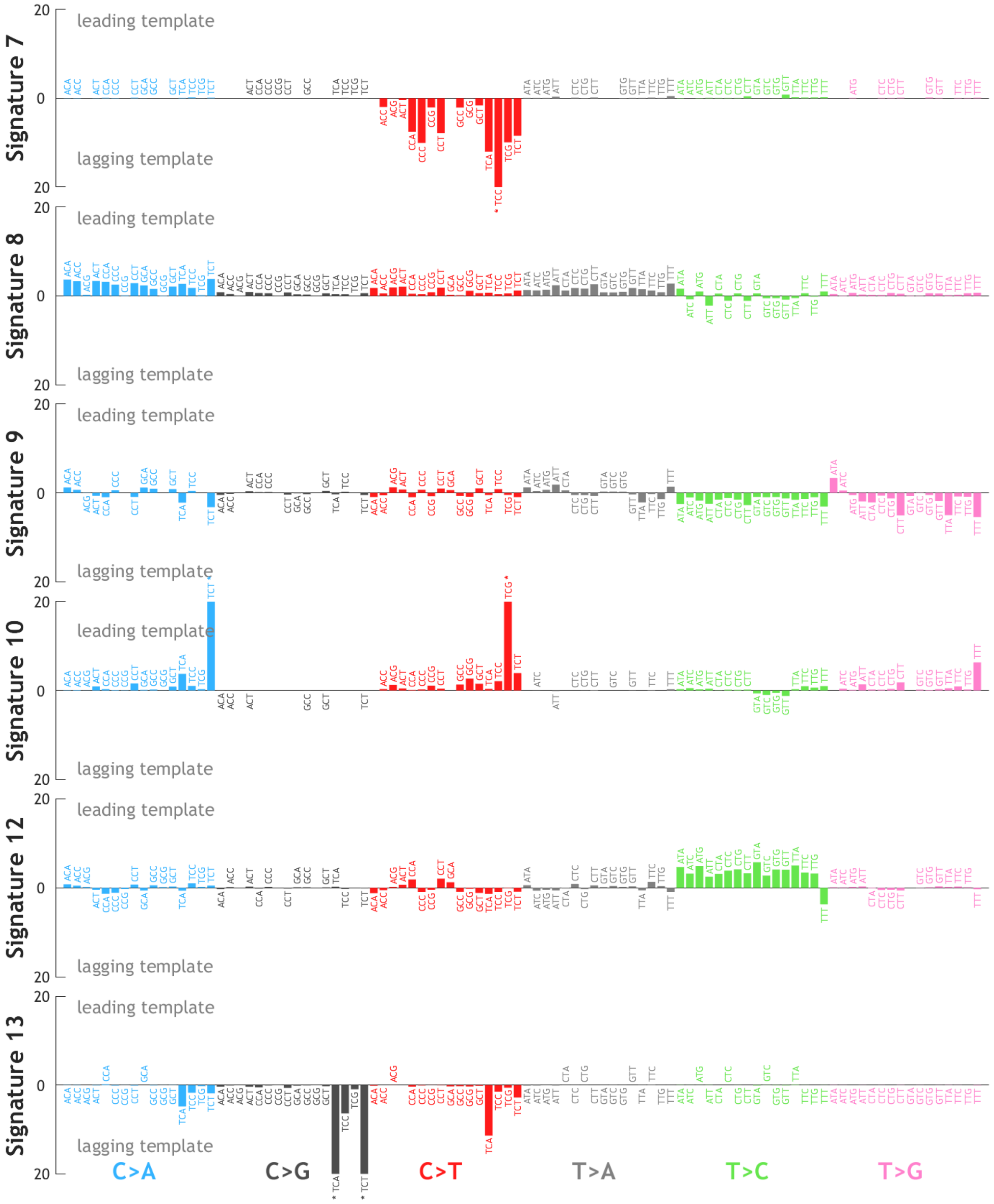

Fig. S2: Directional signatures 7-13. Each of the 96 mutation types is annotated with a dominant direction: leading (pointing up), or lagging (pointing down). Asterisks indicate mutation types exceeding 20%.

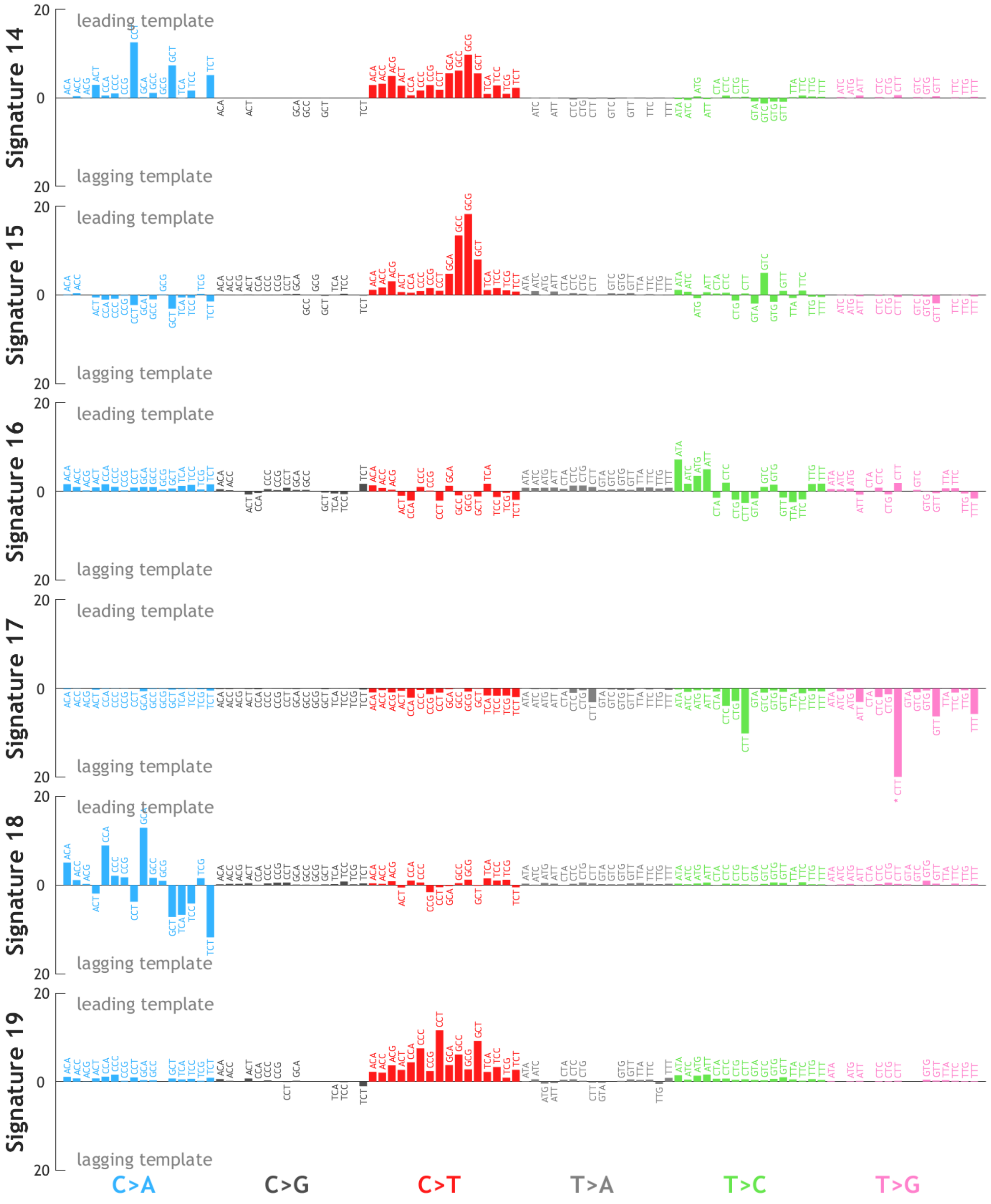

Fig. S3: Directional signatures 14-19. Each of the 96 mutation types is annotated with a dominant direction: leading (pointing up), or lagging (pointing down). Asterisks indicate mutation types exceeding 20%.

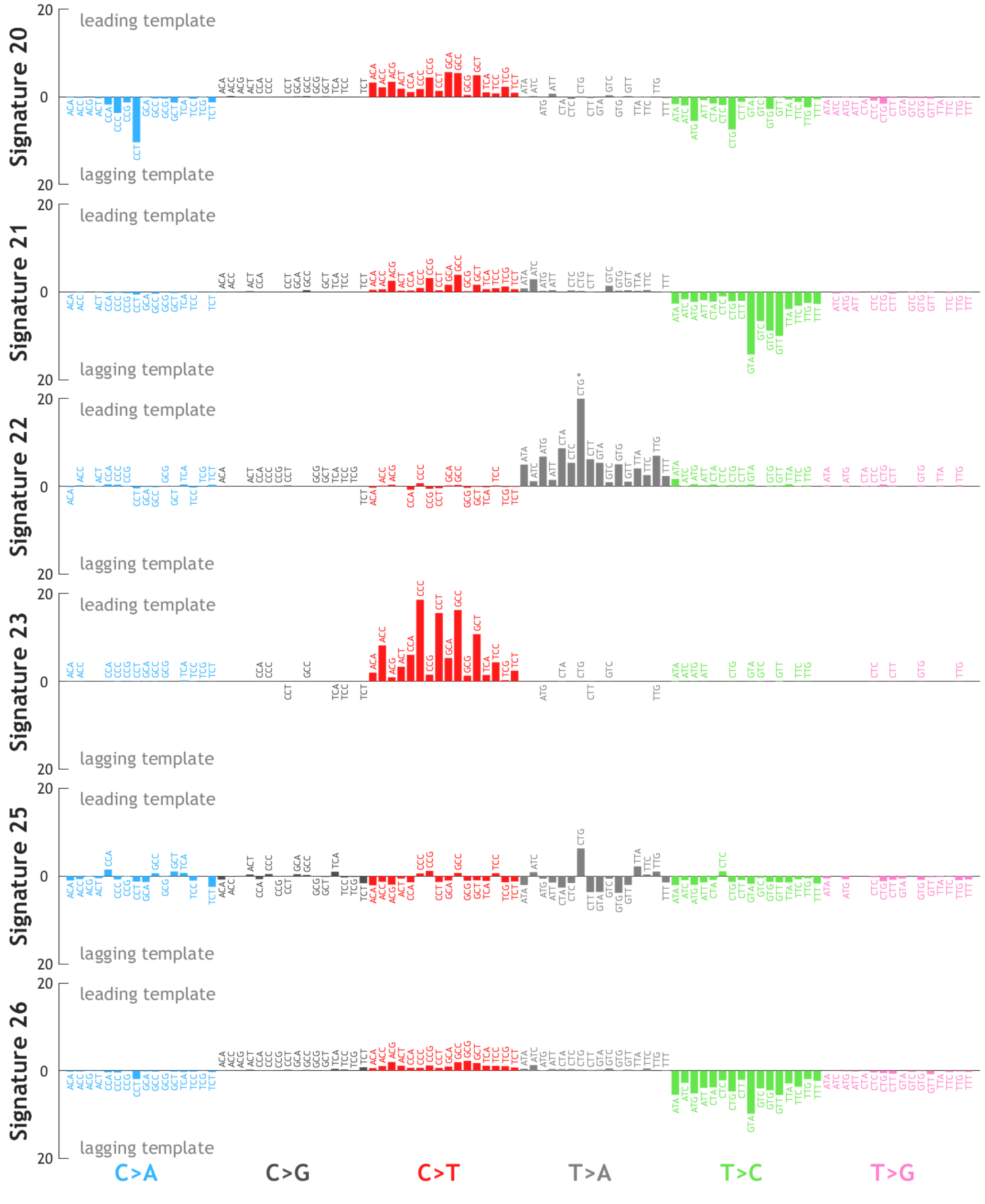

Fig. S4: Directional signatures 20-26. Each of the 96 mutation types is annotated with a dominant direction: leading (pointing up), or lagging (pointing down). Asterisks indicate mutation types exceeding 20%.

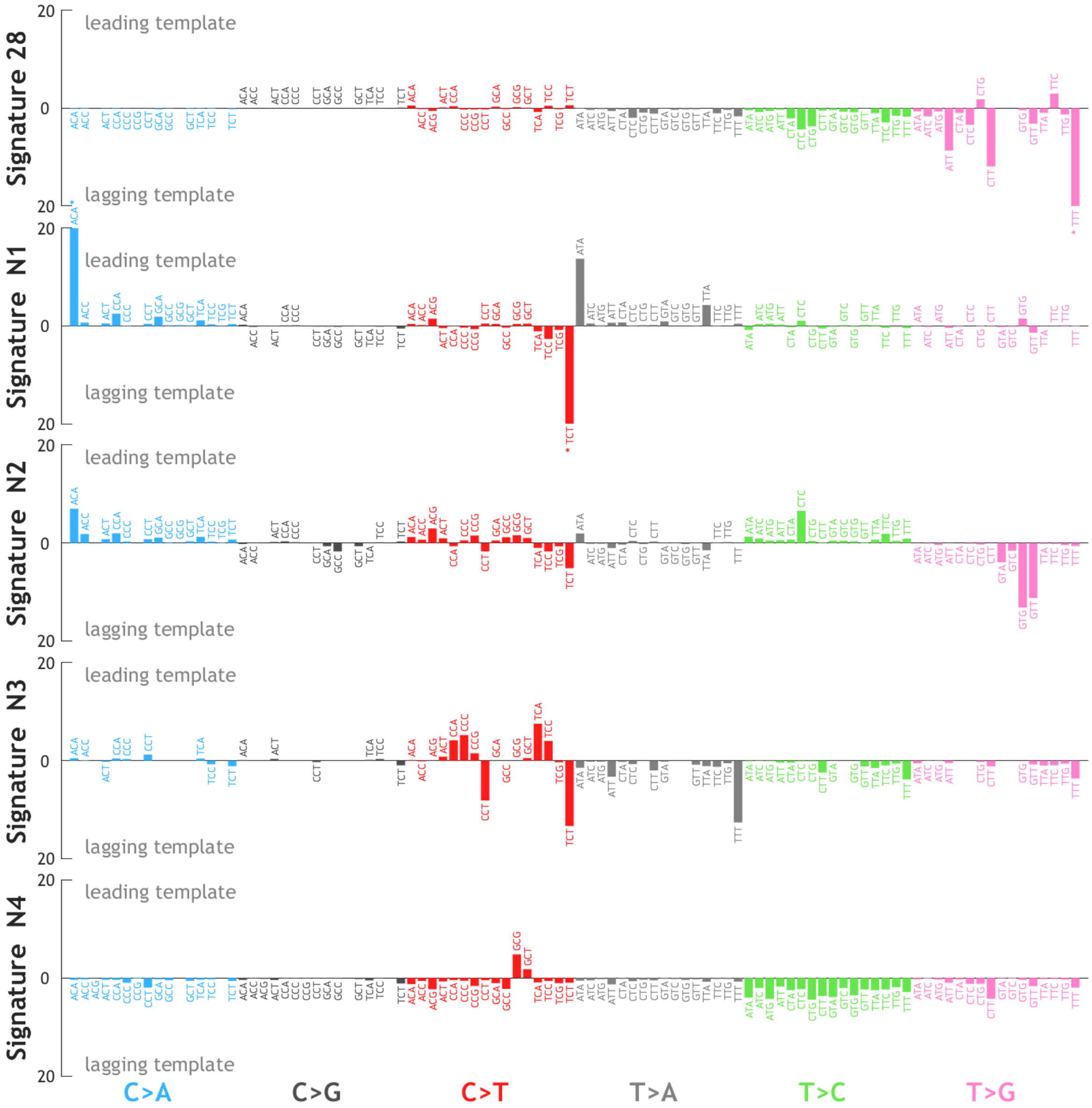

Fig. S5: Directional signatures 28 and N1-N4. Each of the 96 mutation types is annotated with a dominant direction: leading (pointing up), or lagging (pointing down). Asterisks indicate mutation types exceeding 20%.

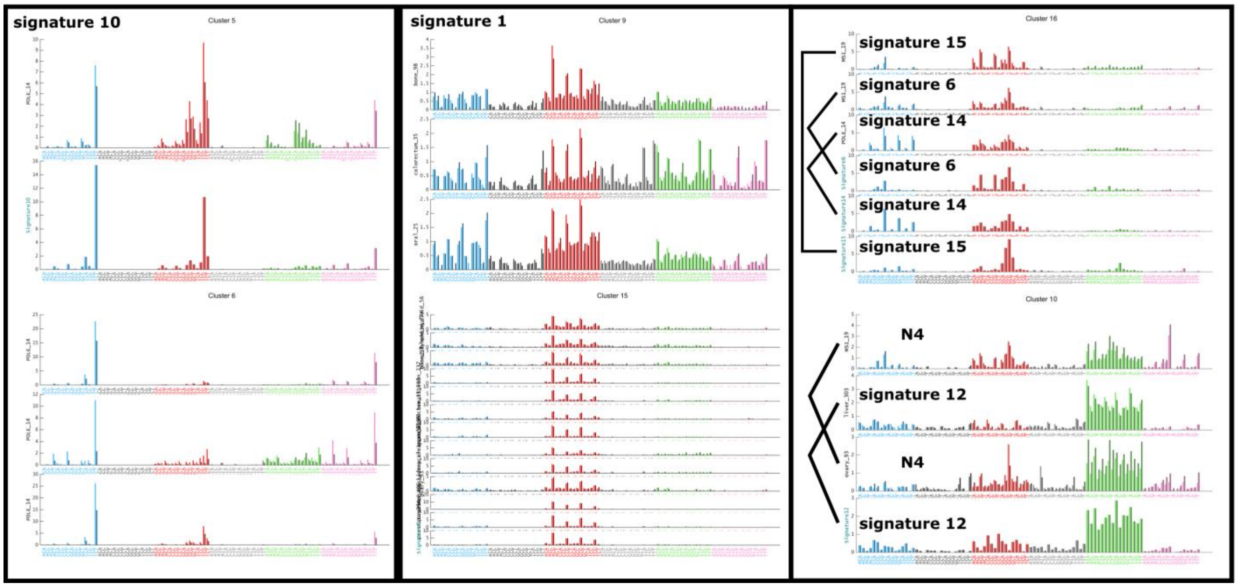

Fig. S6: Mis-clustering was avoided by manual examination (and whenever necessary re-assignment) of all signatures in all clusters: joining two clusters corresponding to signature 10, joining two clusters corresponding to signature 1, splitting one cluster into clusters corresponding to signature 6, signature 14, and signature 15, and finally splitting one cluster into signature 12 and N4.

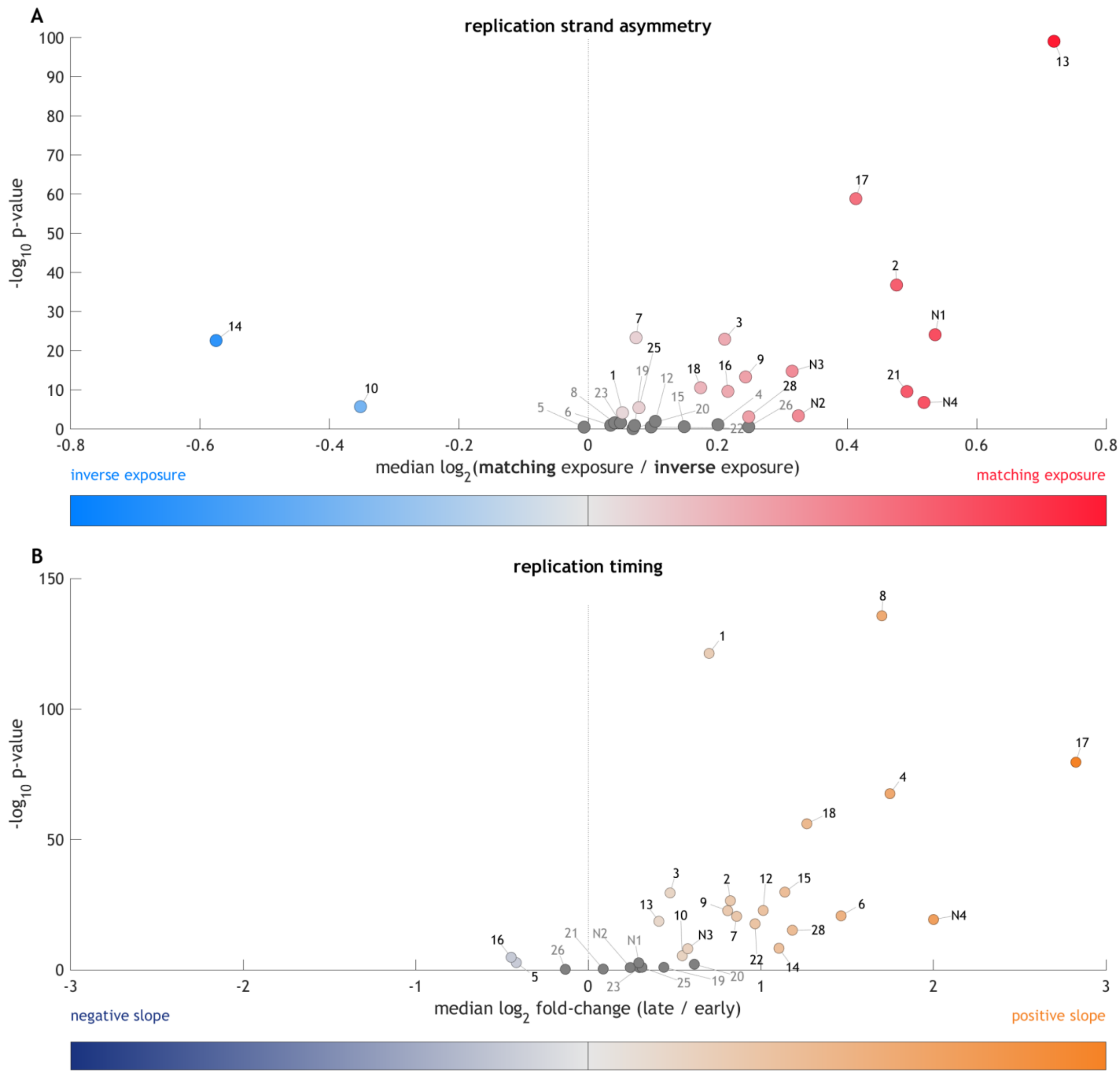

Fig. S7: Most mutational signatures exhibit a significant replication strand asymmetry and/or correlation with replication timing. (A) The log_2-transformed ratio of matching and inverse exposure is computed for each sample and signature. For each signature, the median value of these ratios (in samples exposed to this signature) is plotted against $-log_{10}$ q-value (signtest of strand asymmetry per sample; with Benjamini-Hochberg correction). **(B)** Correlation of exposures with replication timing. The 20 kbp replication domains were divided into four quartiles by their average replication timing (early-replicated in the first quartile, late-replicated in the last quartile) and exposures to signatures were computed in each quartile. Median log_2-transformed fold change from average exposure in early (first quartile) to late (last quartile) is plotted on the x-axis, *i.e.* values on the right denote more mutations in late-replicated regions, values on the left reflect more mutations in early replicating

regions. The y-axis represents significance of the correlation of signature with replication timing in individual samples (signtest of correlation slope per sample; with Benjamini-Hochberg correction).

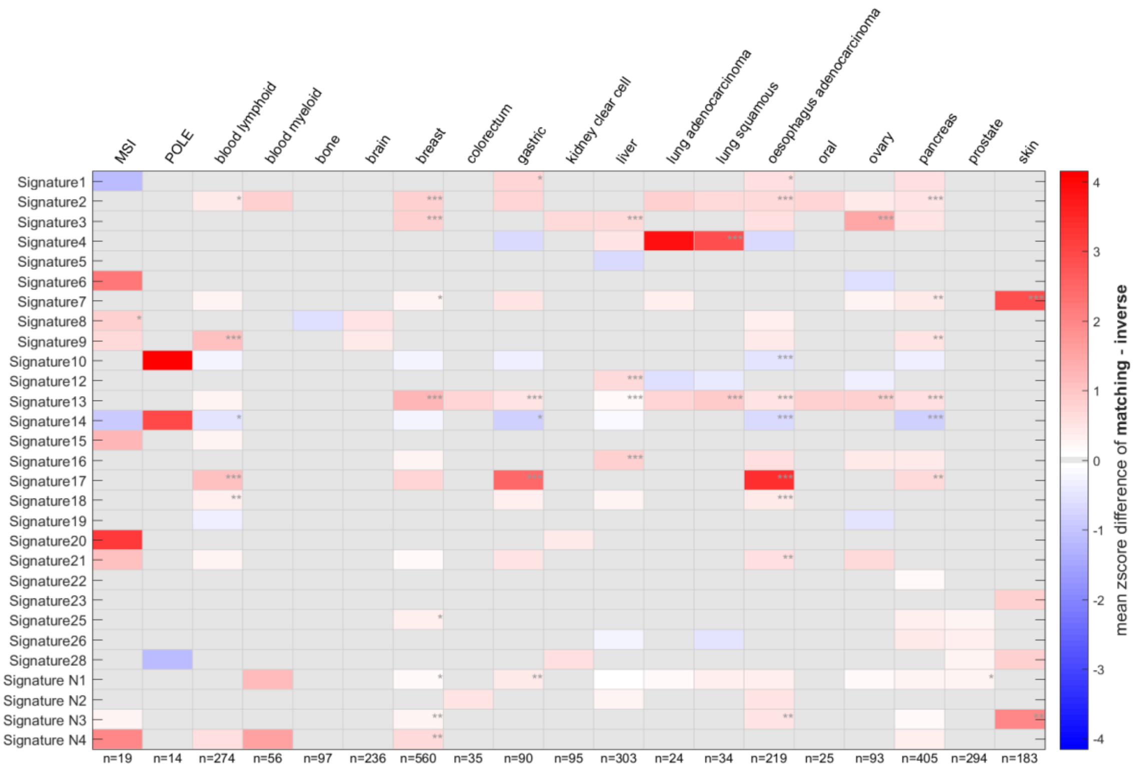

Fig. S8: Mean replication strand asymmetry per signature and cancer type (z-score normalised per sample). Red represents matching strand asymmetry between signature and sample, blue represents inverse asymmetry. Only significant values are shown (non-significant are in grey). Asterisks represent values that also pass Bonferroni correction for multiple testing.

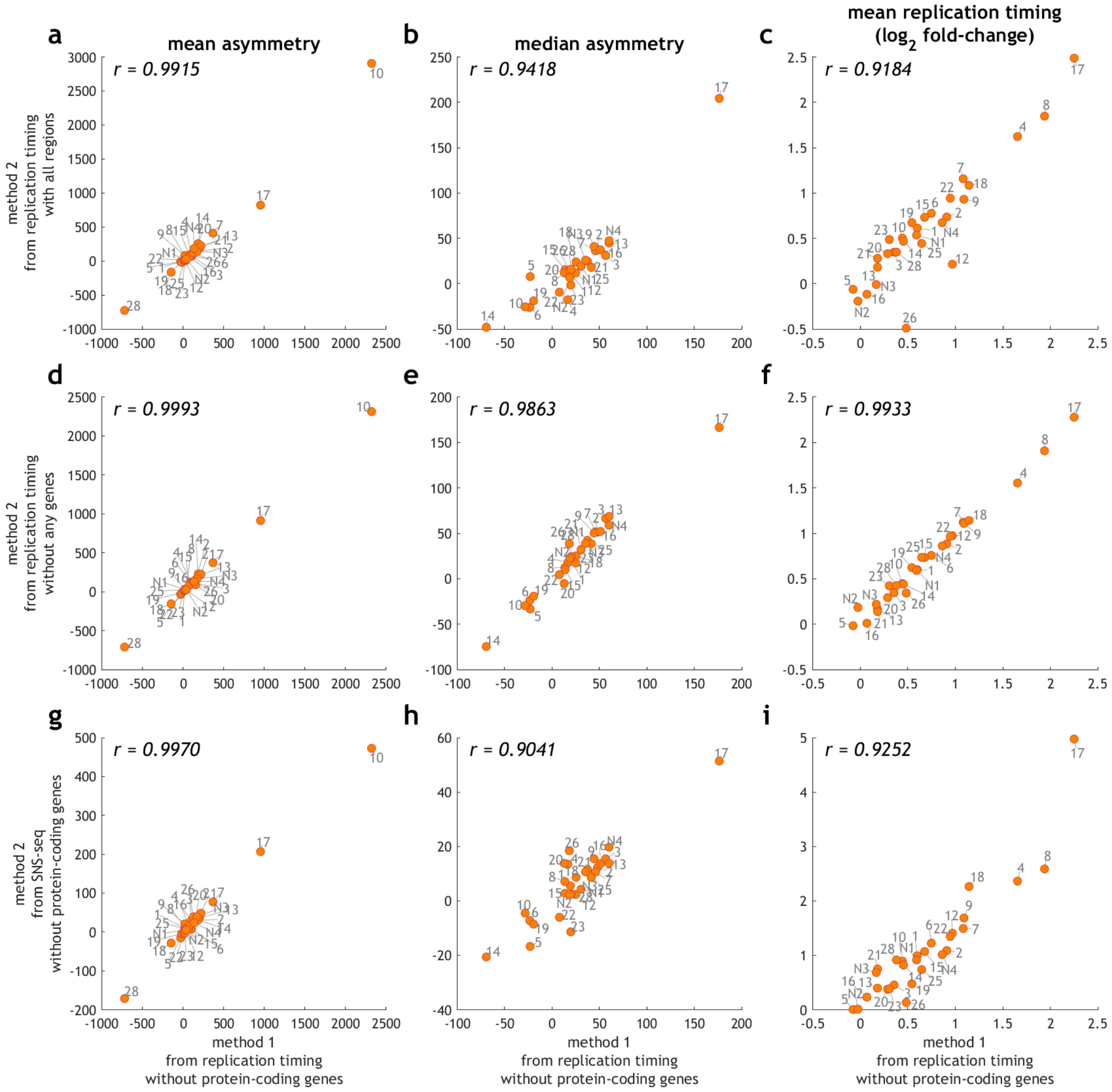

Fig. S9: Inclusion of protein coding genes, exclusion of all or non-protein coding genes, and both methods of estimating direction of replication result in very similar mutation strand asymmetries. Comparison of the method 1 (x axis; protein-coding genes and regions with low mappability were excluded; the direction of replication is derived from replication timing (20 kbp bins annotated as in Haradhvala *et al.*)) with three alternatives on the y-axis: with all regions included (first row), without any genes, including non-protein coding ones, and without regions with low mappability (second row), and with direction of replication derived from measurements of ORIs using NS-seq (1 kbp bins, see Methods; the absolute values of exposures are different between the two methods since regions around ORIs cover fewer bases, and therefore also fewer mutations) (third

row). Each dot represents mean value per signature (first column), median value per signature (second column), or mean log2 fold-change between early and late replicating regions (third column).

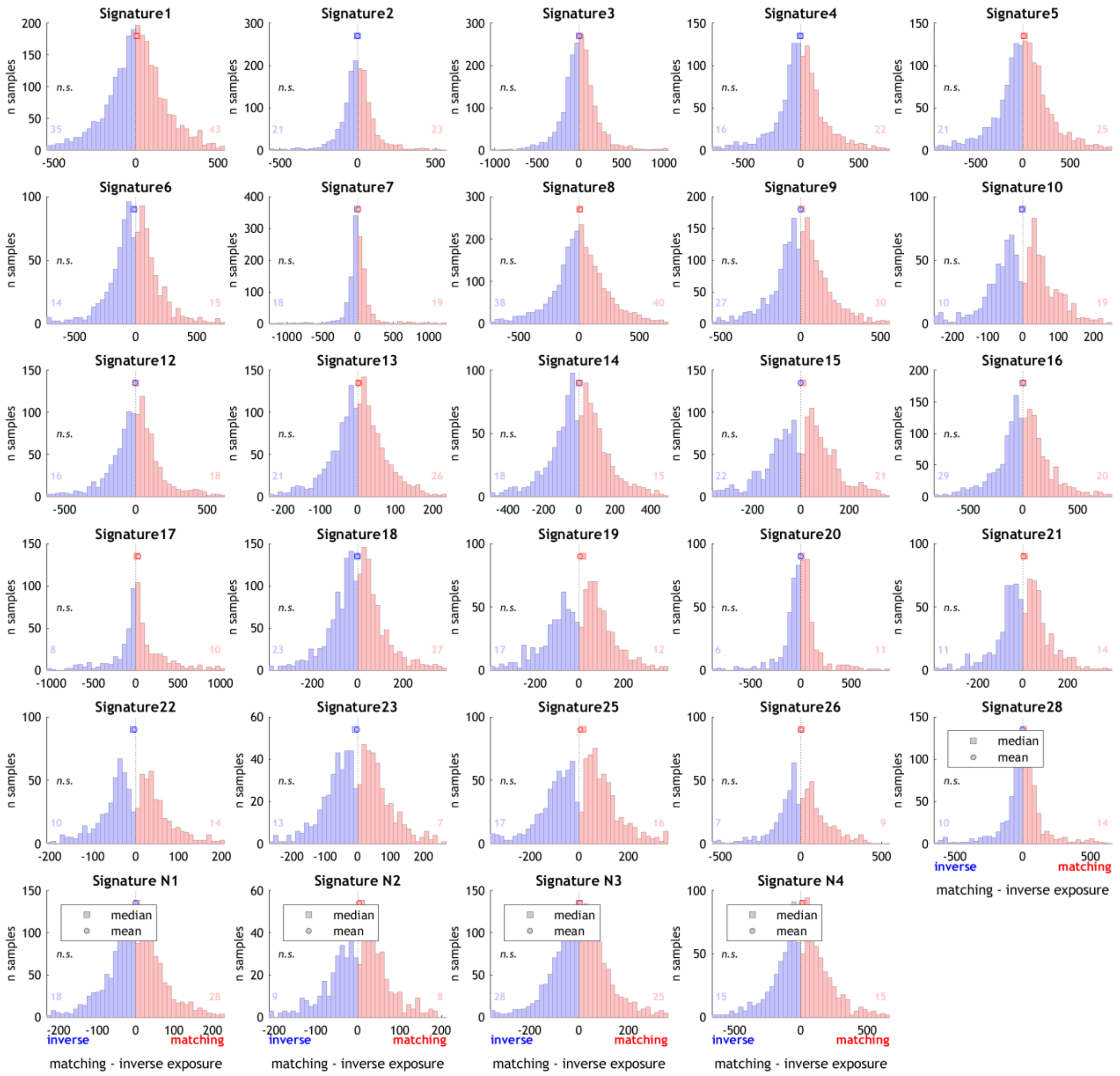

Fig. S10: The strand asymmetry is lost in randomized origins and replication timing domains. Histograms of distributions of strand asymmetry in individual signatures in randomized origins: a list or origins with random locations in the human genome were generated, such that the numbers of origins in individual chromosomes corresponded to the numbers detected using the main method (from replication timing). Up to 100 regions of 20kbp on both sides were taken into account into the analysis. For each signature, ranksum test was used to evaluate strand asymmetry, and Bonferroni correction for multiple testing applied; all resulting p-values in this figure were non-significant (n.s.).

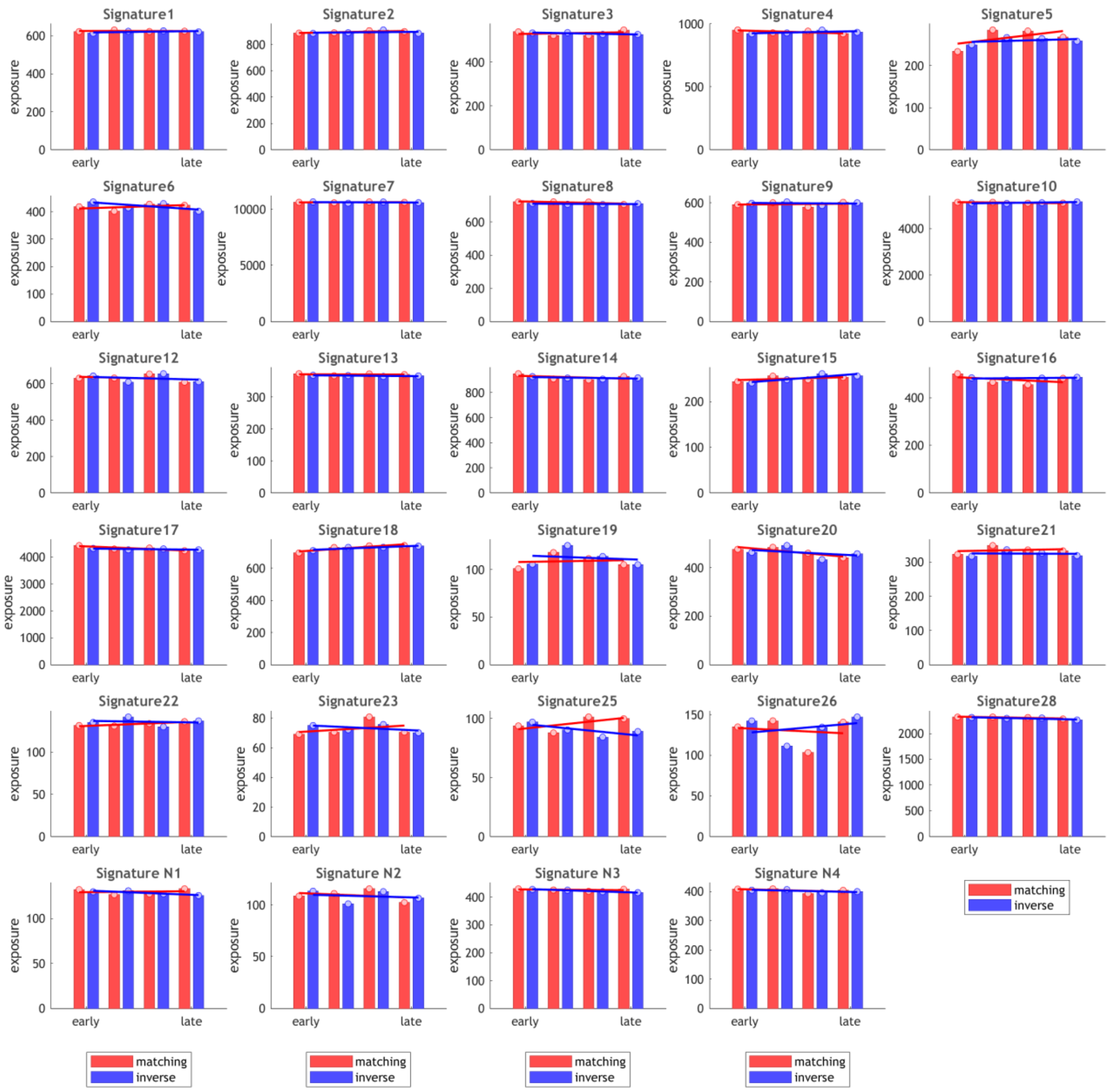

Fig. S11: The correlation with replication timing is lost in randomized origins and replication timing domains. Average correlation of exposures (matching and inverse separately) with replication timing in individual signatures using randomized origins, generated as described in Fig. 2–figure supplement 3. The original values of replication timing in the individual regions were randomly permuted in the generated domains.

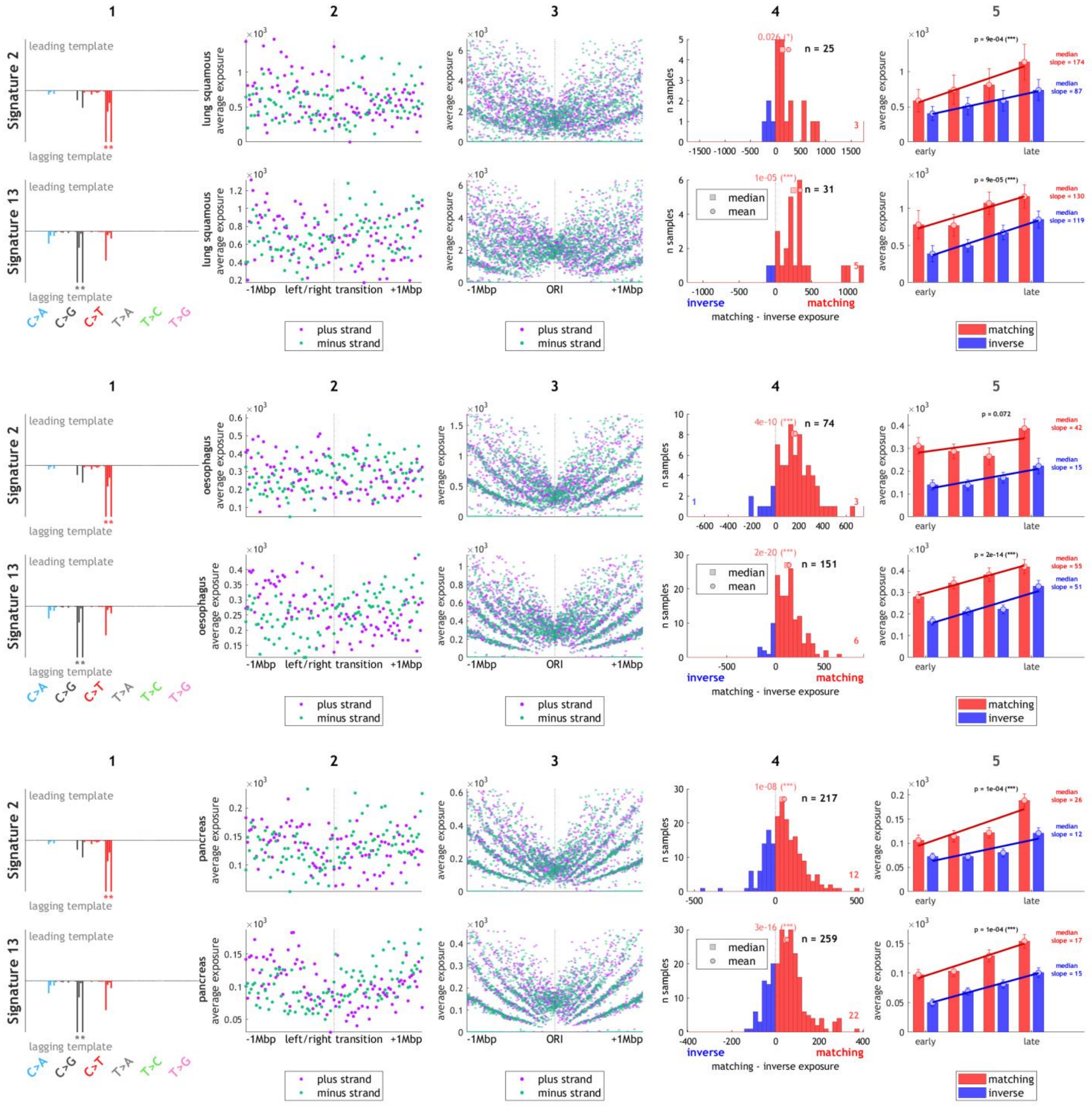

Fig. S12: Replication strand asymmetry and replication timing in APOBEC signatures. Columns show directional signature (column 1), distribution around timing transition regions (column 2) and around replication origins (column 3), per-patient mutation strand asymmetry (column 4; non-significant asymmetry is shown in light-colored histogram) and correlation with replication timing (column 5), as described in Fig 3.

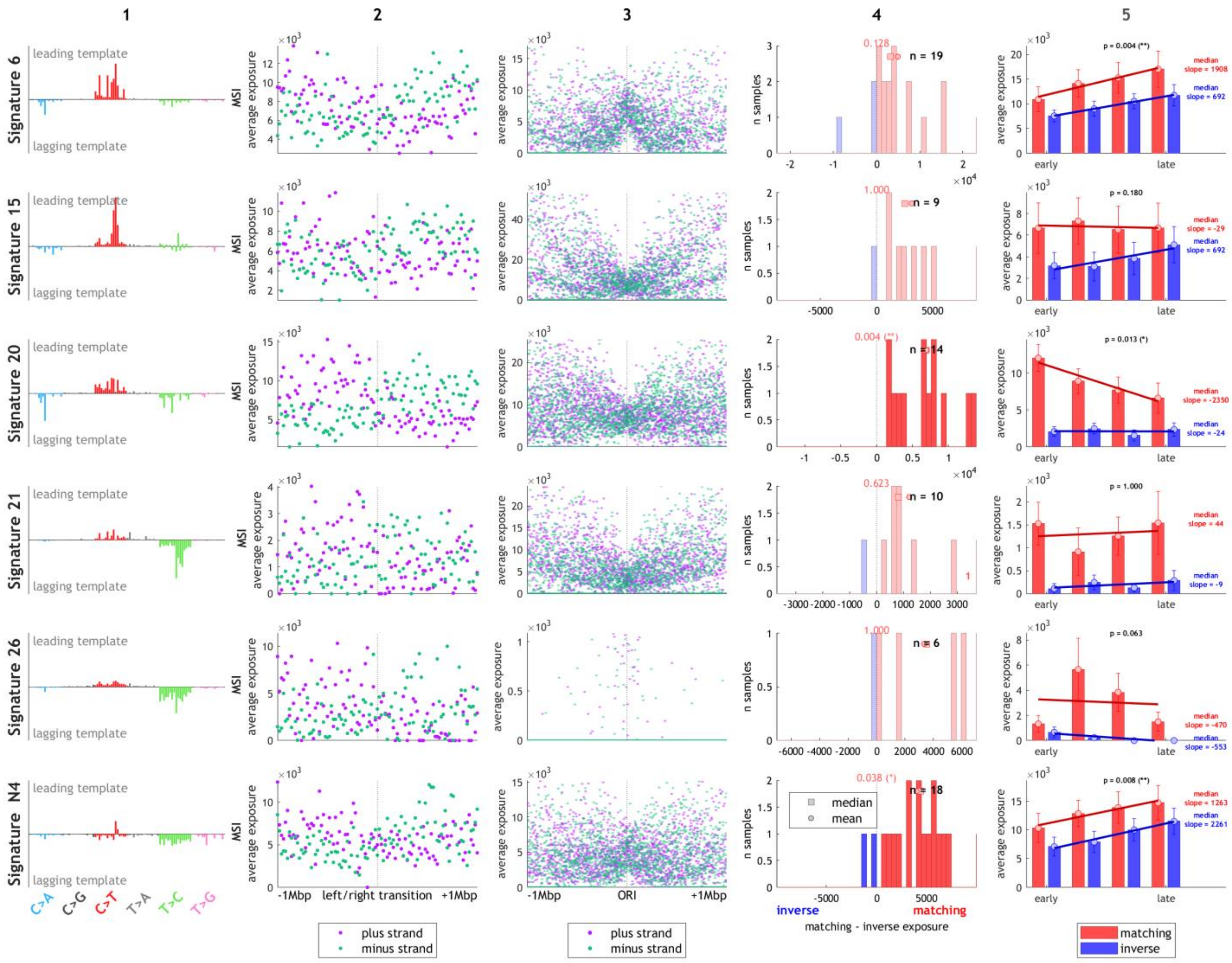

Fig. S13: Replication strand asymmetry and replication timing in MMR signatures in MSI samples. Columns show directional signature (column 1), distribution around timing transition regions (column 2) and around replication origins (column 3), per-patient mutation strand asymmetry (column 4; non-significant asymmetry is shown in light-colored histogram) and correlation with replication timing (column 5), as described in Fig 3.

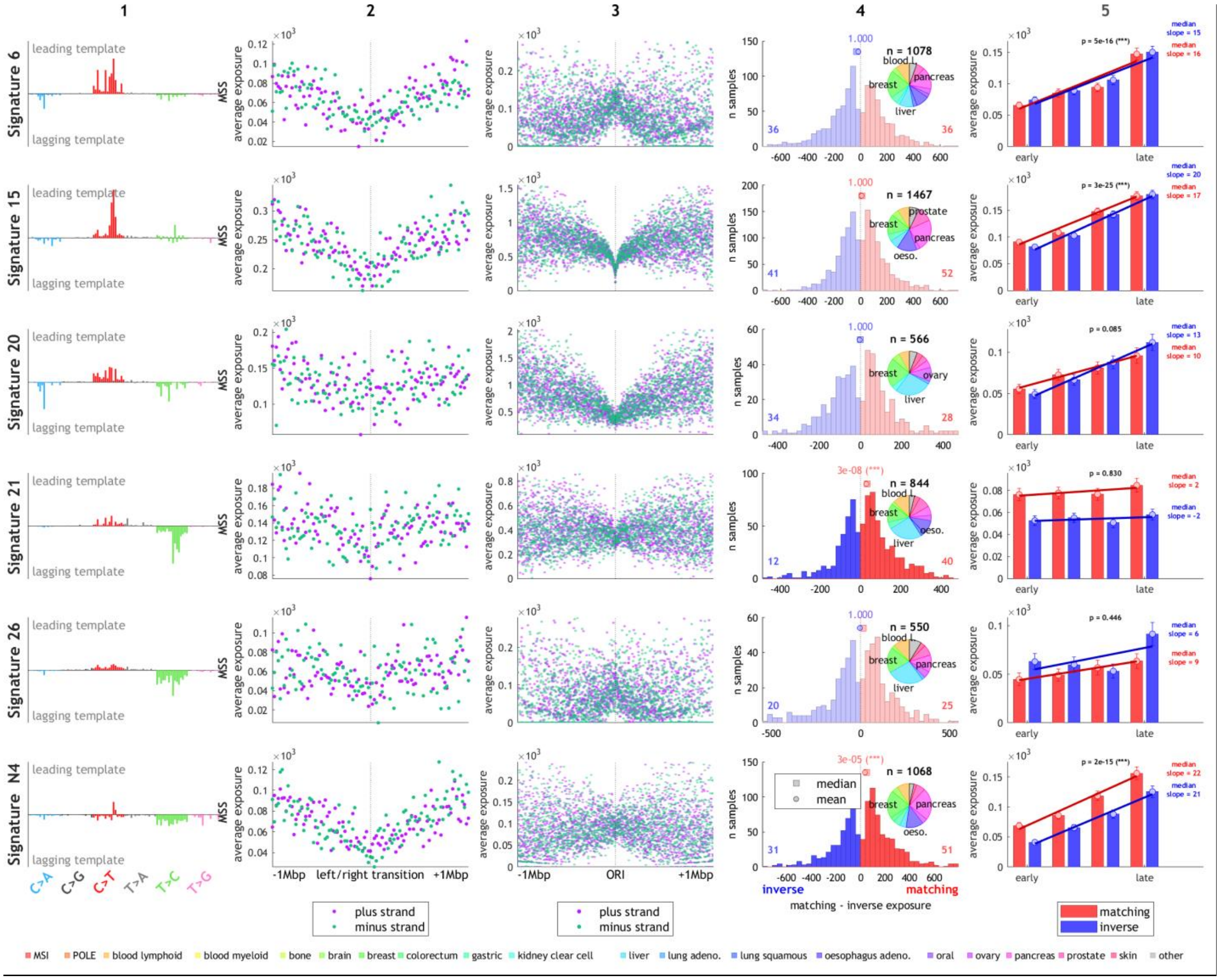

Fig. S14: Replication strand asymmetry and replication timing in MMR signatures in microsatellite stable samples (MSS). Columns show directional signature (column 1), distribution around timing transition regions (column 2) and around replication origins (column 3), per-patient mutation strand asymmetry (column 4; non-significant asymmetry is shown in light-colored histogram) and correlation with replication timing (column 5), as described in Fig 3.

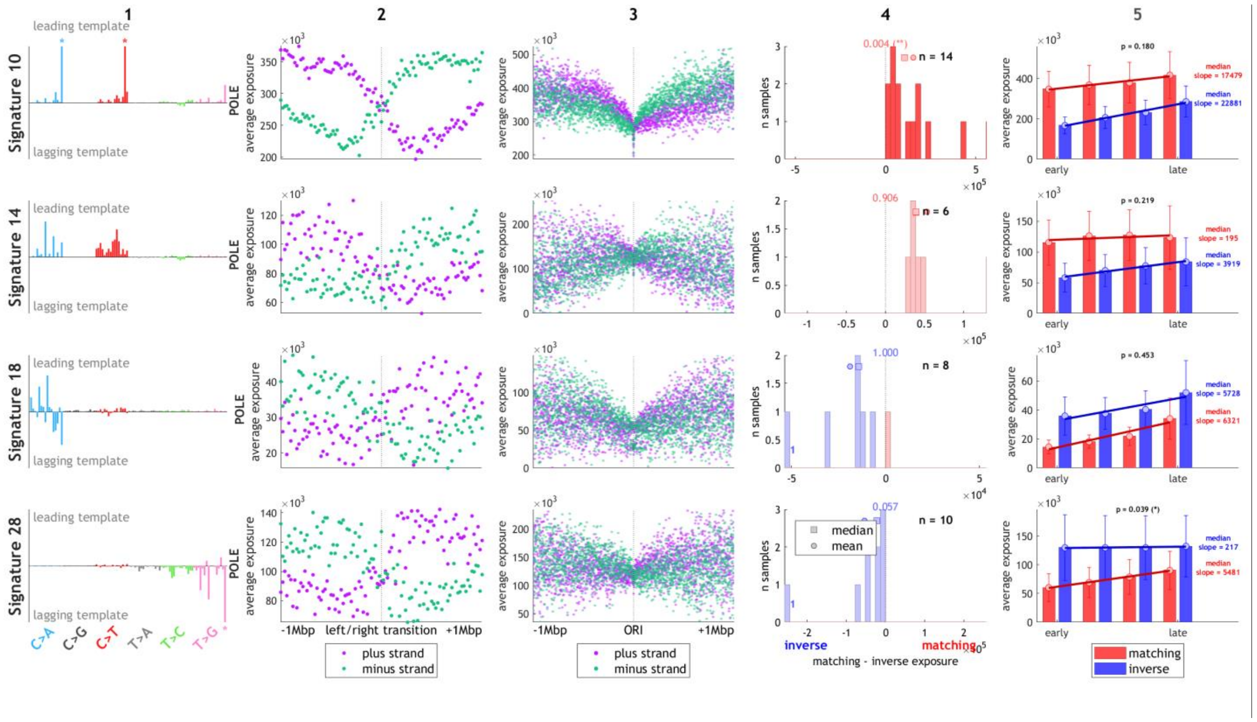

Fig. S15: Replication strand asymmetry and replication timing in signatures detected in POLE-M samples. Columns show directional signature (column 1), distribution around timing transition regions (column 2) and around replication origins (column 3), per-patient mutation strand asymmetry (column 4; non-significant asymmetry is shown in light-colored histogram) and correlation with replication timing (column 5), as described in Fig 3.

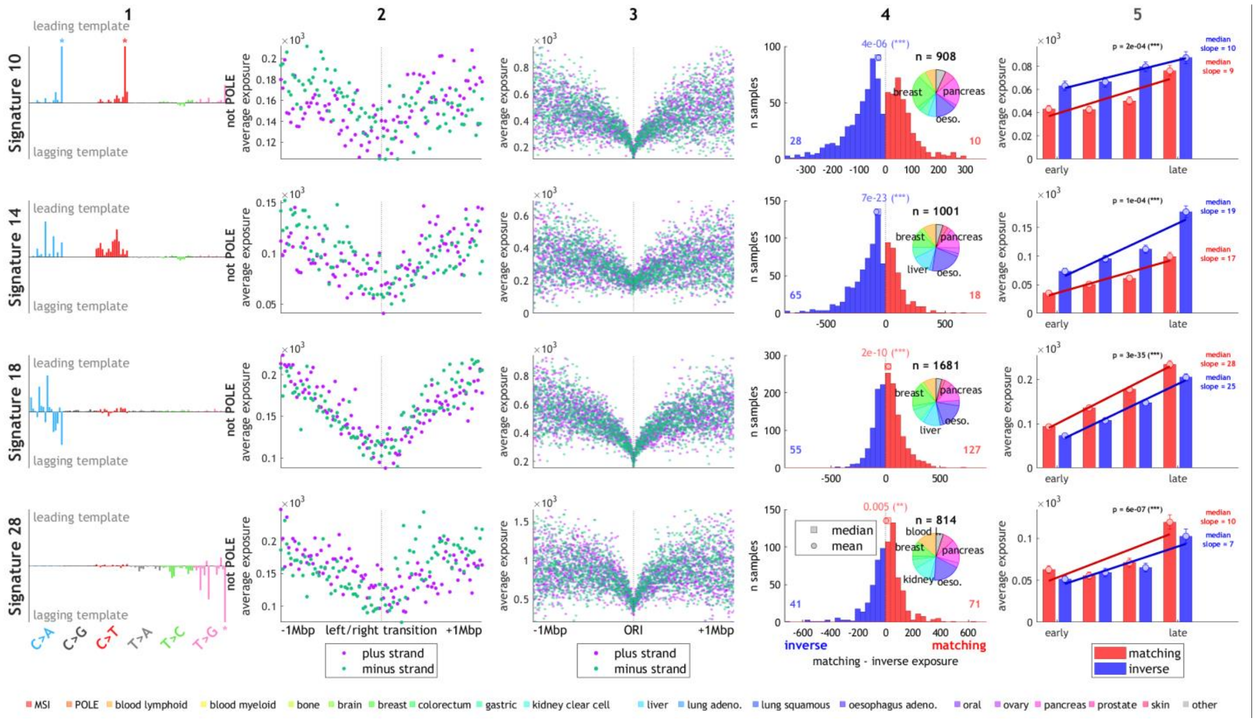

Fig. S16: Replication strand asymmetry and replication timing in signatures 10, 14, 18, and 28, in POLE-WT samples. Columns show directional signature (column 1), distribution around timing transition regions (column 2) and around replication origins (column 3), per-patient mutation strand asymmetry (column 4; non-significant asymmetry is shown in light-colored histogram) and correlation with replication timing (column 5), as described in Fig 3.

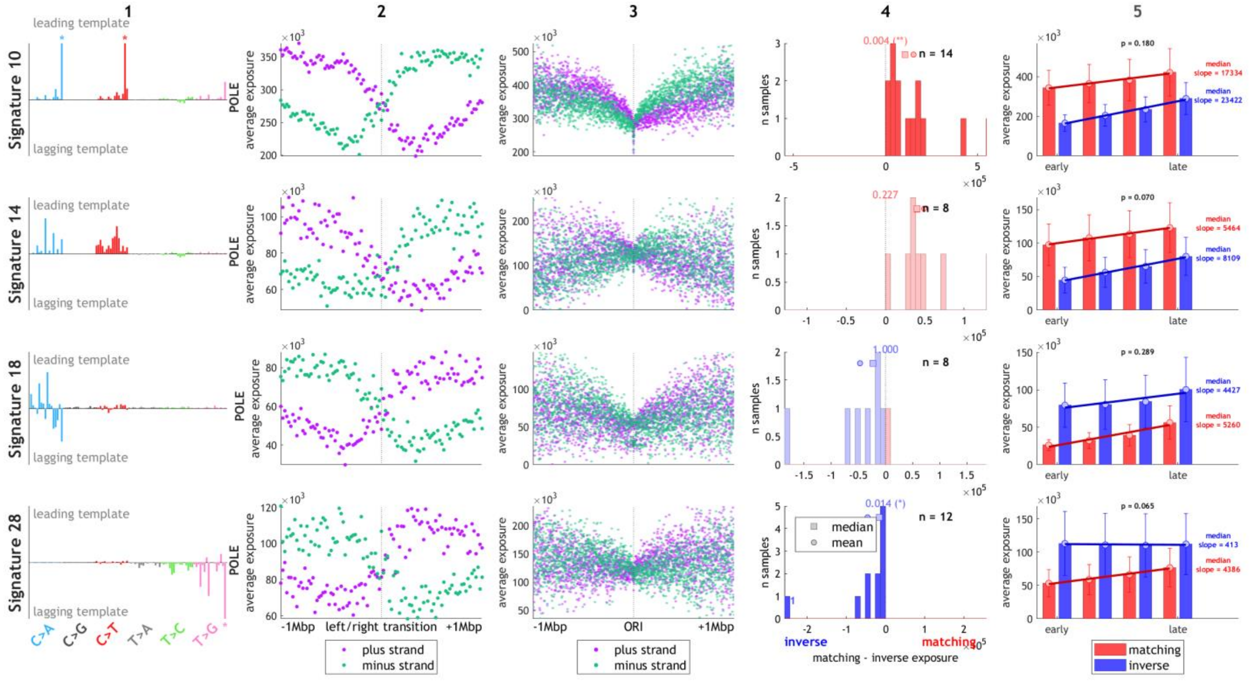

Fig. S17: Evaluation of robustness to noise: replication strand asymmetry and replication timing in signatures detected in POLE-M samples. Results of evaluation of robustness to noise in signature exposures using 1000 perturbations (see Methods). Columns show directional signature (column 1), distribution around timing transition regions (column 2) and around replication origins (column 3), per-patient mutation strand asymmetry (column 4; non-significant asymmetry is shown in light-colored histogram) and correlation with replication timing (column 5), as described in Fig 3.

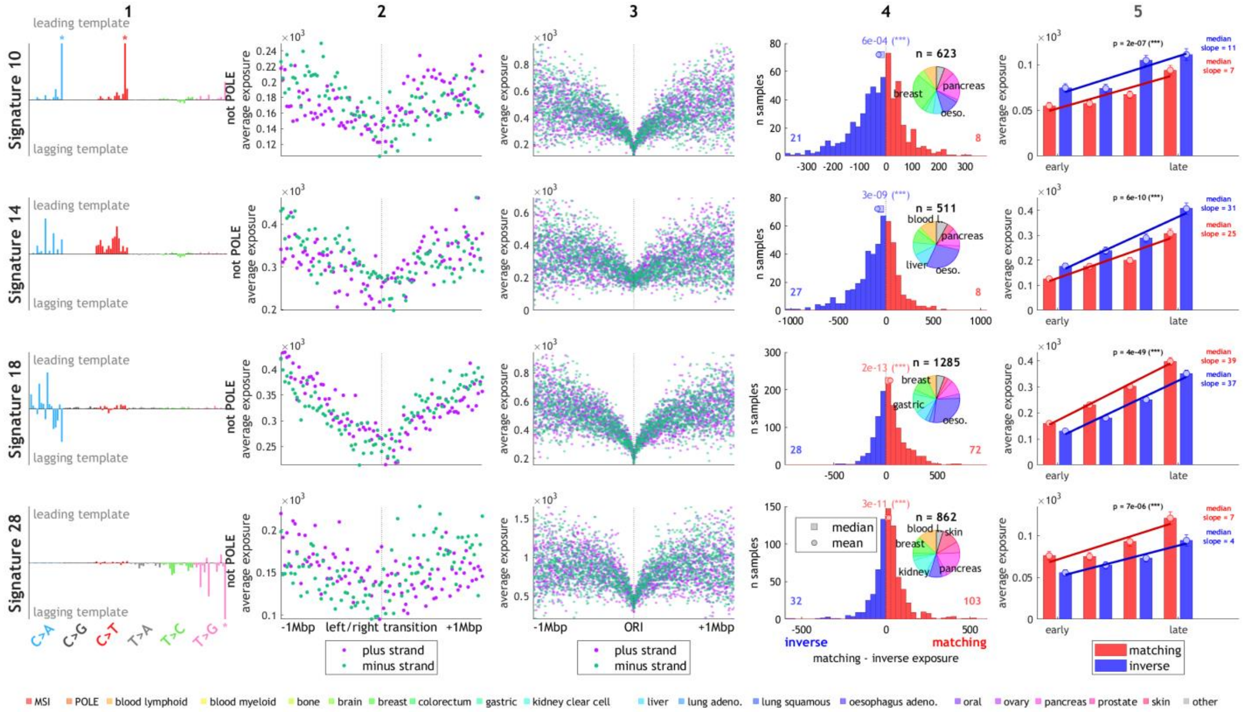

Fig. S18: Evaluation of robustness to noise: replication strand asymmetry and replication timing in signatures 10, 14, 18, and 28, in POLE-WT samples. Results of evaluation of robustness to noise in signature exposures using 1000 perturbations (see Methods). Columns show directional signature (column 1), distribution around timing transition regions (column 2) and around replication origins (column 3), per-patient mutation strand asymmetry (column 4; non-significant asymmetry is shown in light-colored histogram) and correlation with replication timing (column 5), as described in Fig 3.

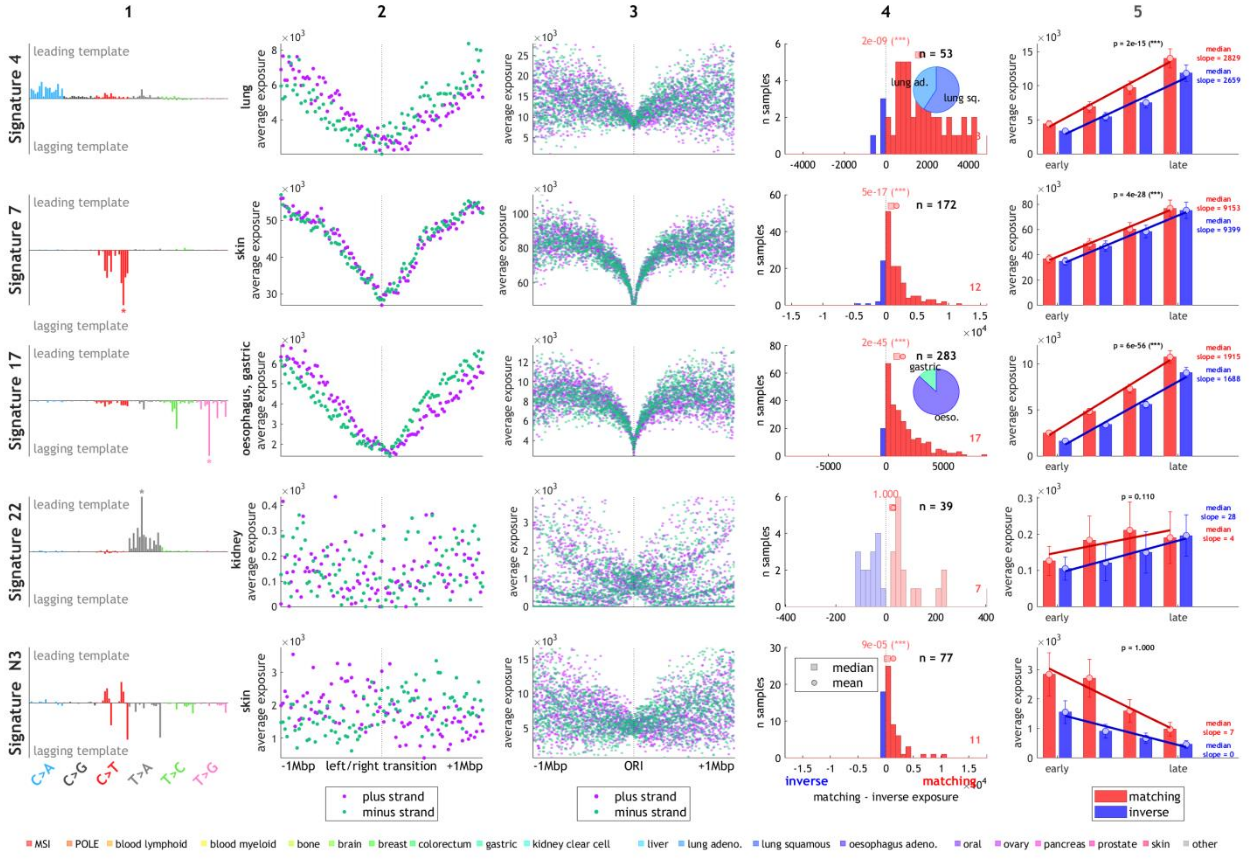

Fig. S19: Replication strand asymmetry and replication timing in three mutagen signatures (4 in lung cancer samples, 7 in skin cancer samples, 22 in kidney cancer samples) and two signatures with unknown etiology: 17 (oesophagus and gastric cancer samples) and N3 (skin cancer samples). Columns show directional signature (column 1), distribution around timing transition regions (column 2) and around replication origins (column 3), per-patient mutation strand asymmetry (column 4; non-significant asymmetry is shown in light-colored histogram) and correlation with replication timing (column 5), as described in Fig 3.

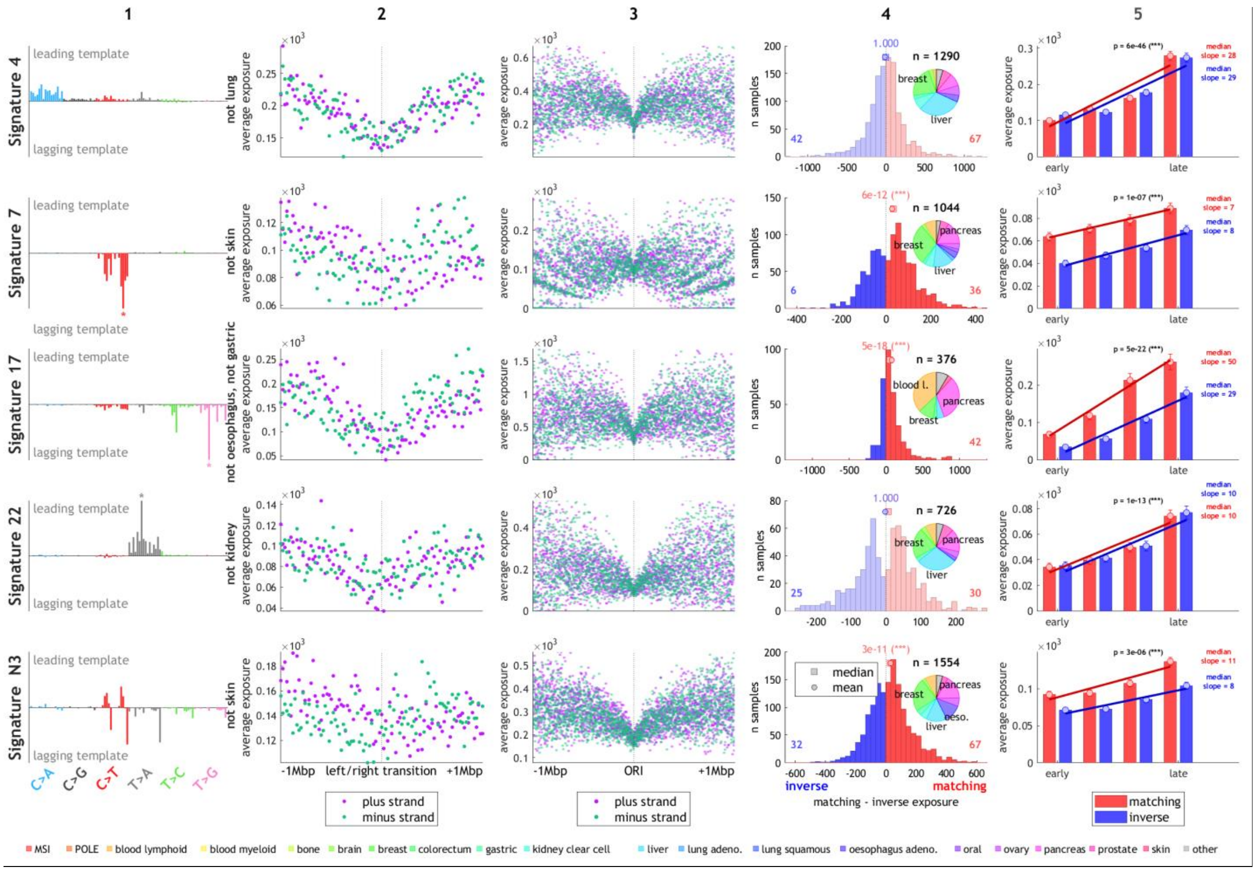

Fig. S20: Replication strand asymmetry and replication timing in signatures 4, 7, 17, 22, and N3 shown in other than their dominant tissue. Columns show directional signature (column 1), distribution around timing transition regions (column 2) and around replication origins (column 3), per-patient mutation strand asymmetry (column 4; non-significant asymmetry is shown in light-colored histogram) and correlation with replication timing (column 5), as described in Fig 3.

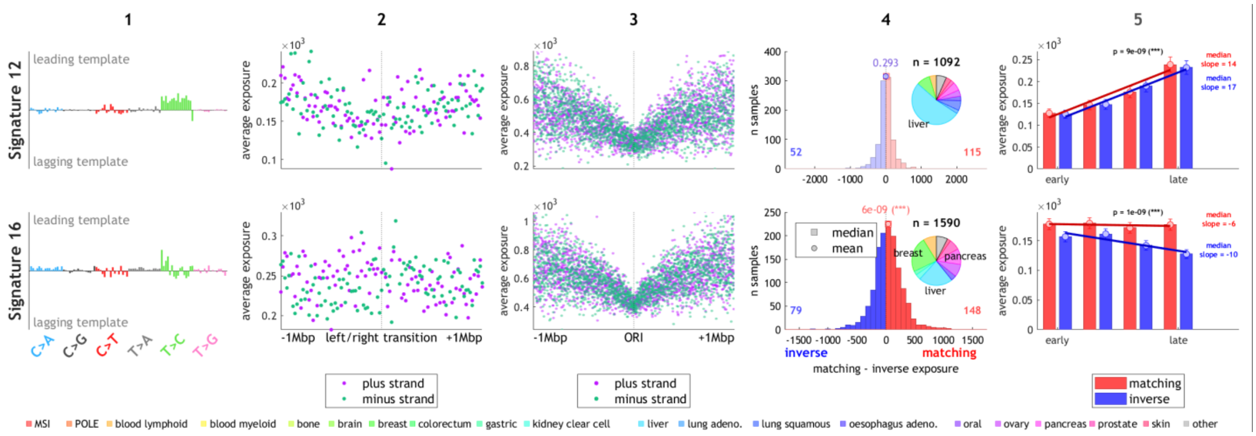

Fig. S21: Replication strand asymmetry and replication timing in liver-associated signatures. Columns show directional signature (column 1), distribution around timing transition regions (column 2) and around replication origins (column 3), per-

patient mutation strand asymmetry (column 4; non-significant asymmetry is shown in light-colored histogram) and correlation with replication timing (column 5), as described in Fig 3.

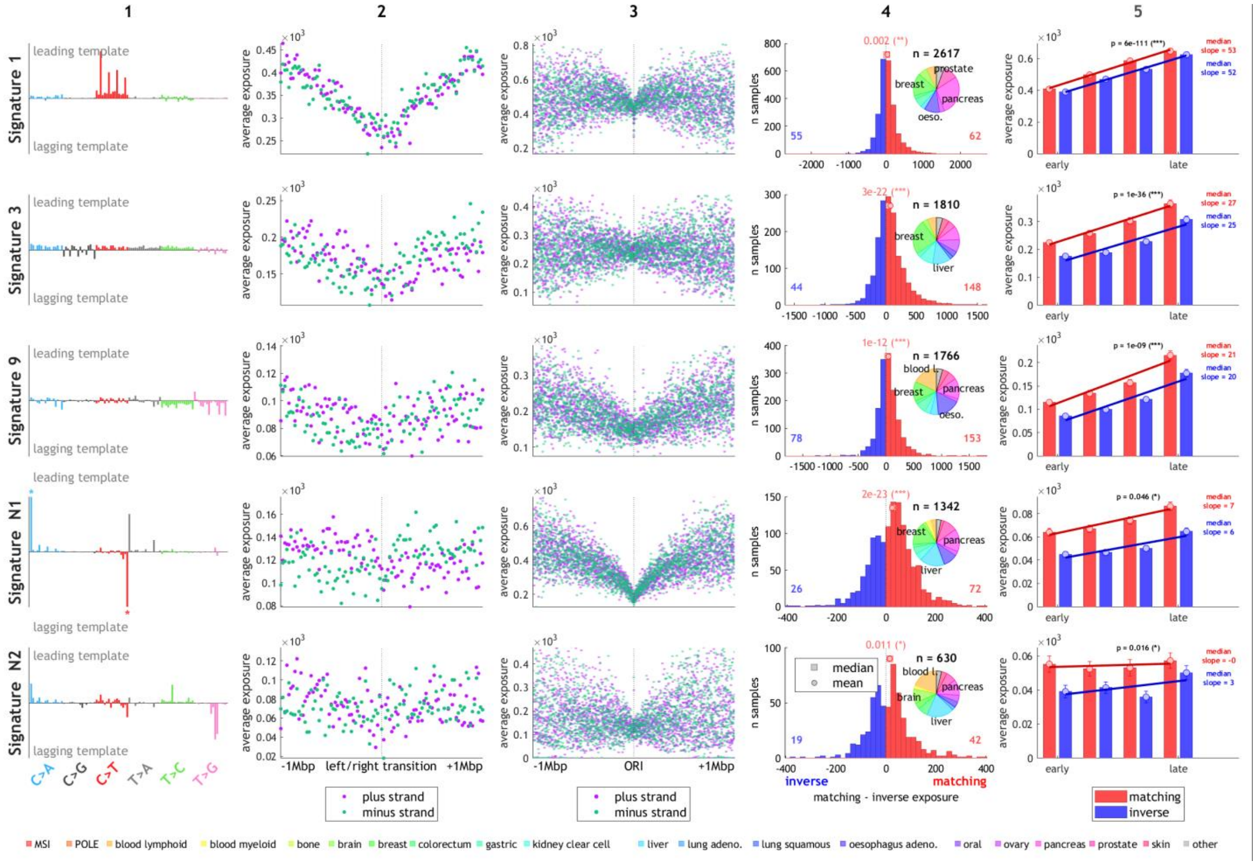

Fig. S22: Replication strand asymmetry and replication timing in signatures 1, 3, 9, N1, N2. Columns show directional signature (column 1), distribution around timing transition regions (column 2) and around replication origins (column 3), per-patient mutation strand asymmetry (column 4; non-significant asymmetry is shown in light-colored histogram) and correlation with replication timing (column 5), as described in Fig 3.

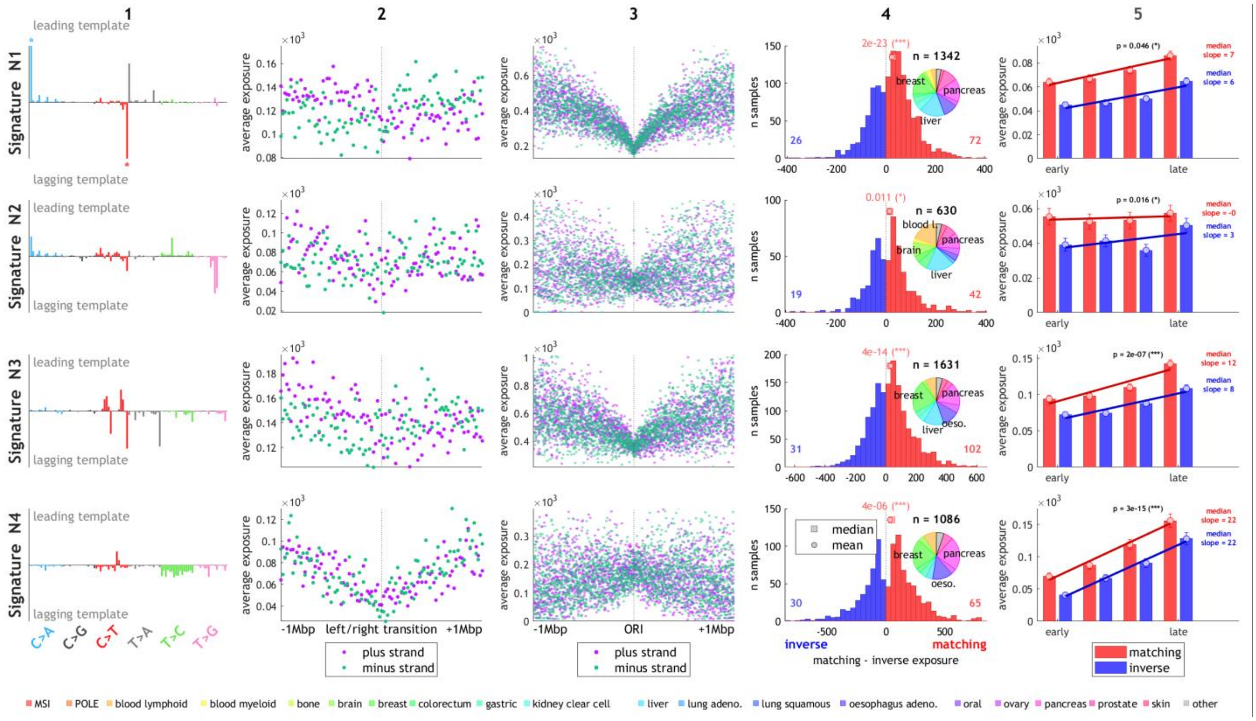

Fig. S23: Replication strand asymmetry and replication timing in the four new signatures: N1, N2, N3, and N4. Columns show directional signature (column 1), distribution around timing transition regions (column 2) and around replication origins (column 3), per-patient mutation strand asymmetry (column 4; non-significant asymmetry is shown in light-colored histogram) and correlation with replication timing (column 5), as described in Fig 3.

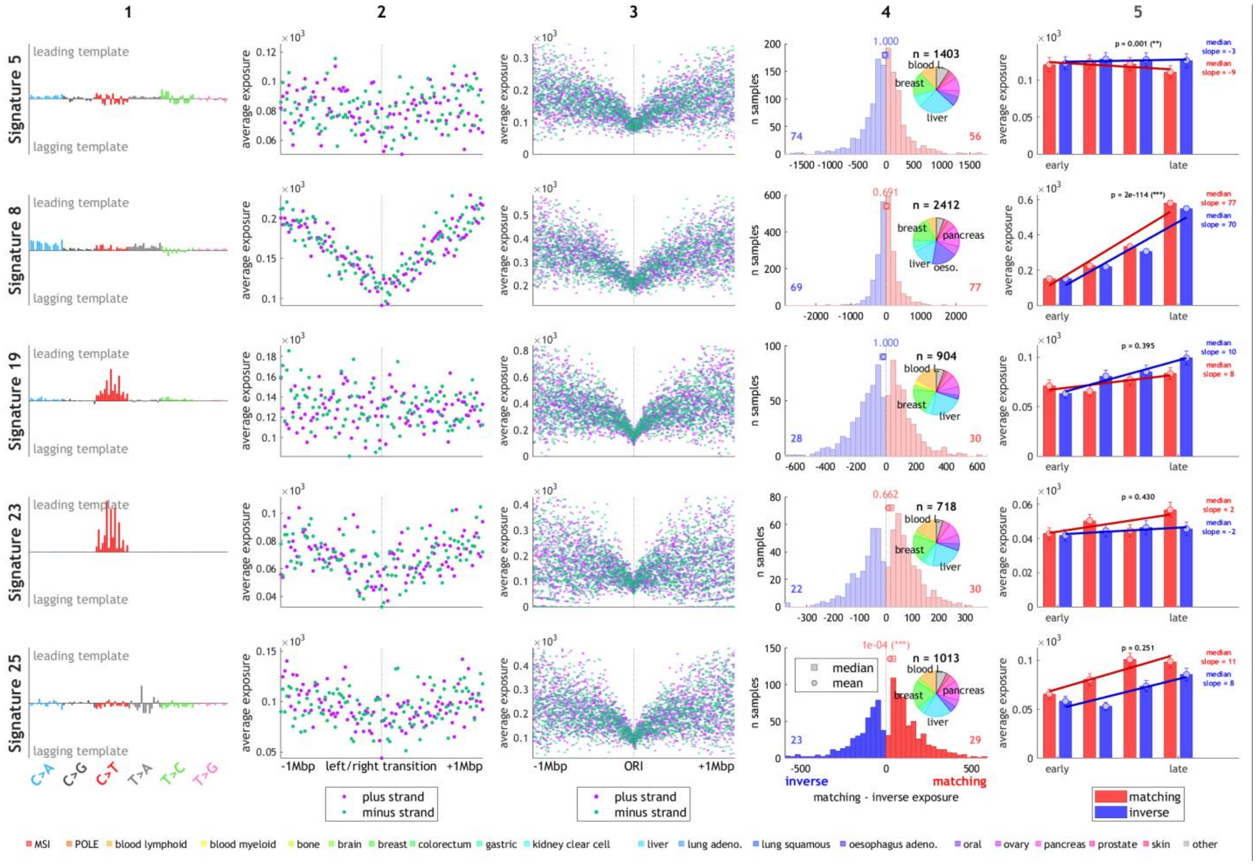

Fig. S24: Replication strand asymmetry and replication timing in signatures with little or no asymmetry: 5, 8, 19, 23, and 25. Columns show directional signature (column 1), distribution around timing transition regions (column 2) and around replication origins (column 3), per-patient mutation strand asymmetry (column 4; non-significant asymmetry is shown in light-colored histogram) and correlation with replication timing (column 5), as described in Fig 3.

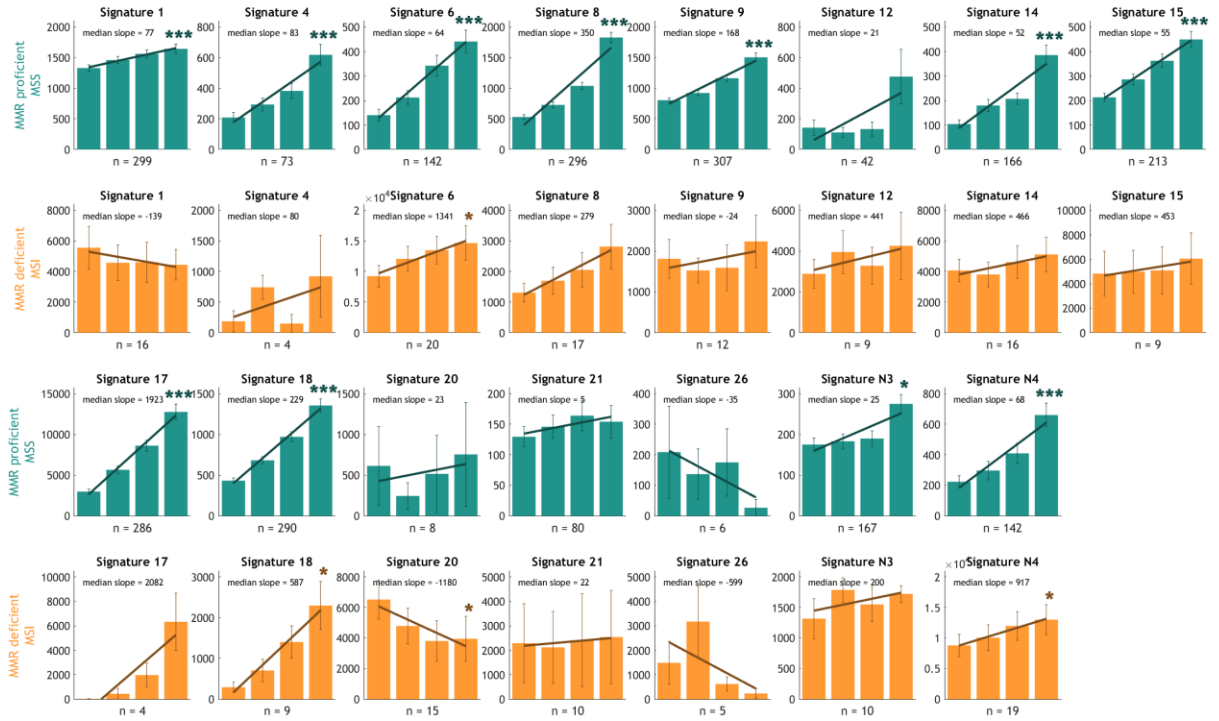

Fig. S25: Comparison of effects of replication timing on mutational signatures in MSI and MSS samples. Average exposure to mutational signatures with respect to replication timing quartiles: early (first bin) to late (last bin). Samples are grouped according to their MSI status: MSS in green (first and third row) and MSI in orange (second and fourth row). The error bars represent standard error of the mean. Signatures with exposure of at least 10 in at least 4 MSI samples were included. The analysis was performed on tissues with MSI samples: gastric, colorectum, oesophageal adenocarcinoma. The asterisks denote signatures with significant effect of replication timing (signtest of the slopes, Benjamini-Hochberg corrected).

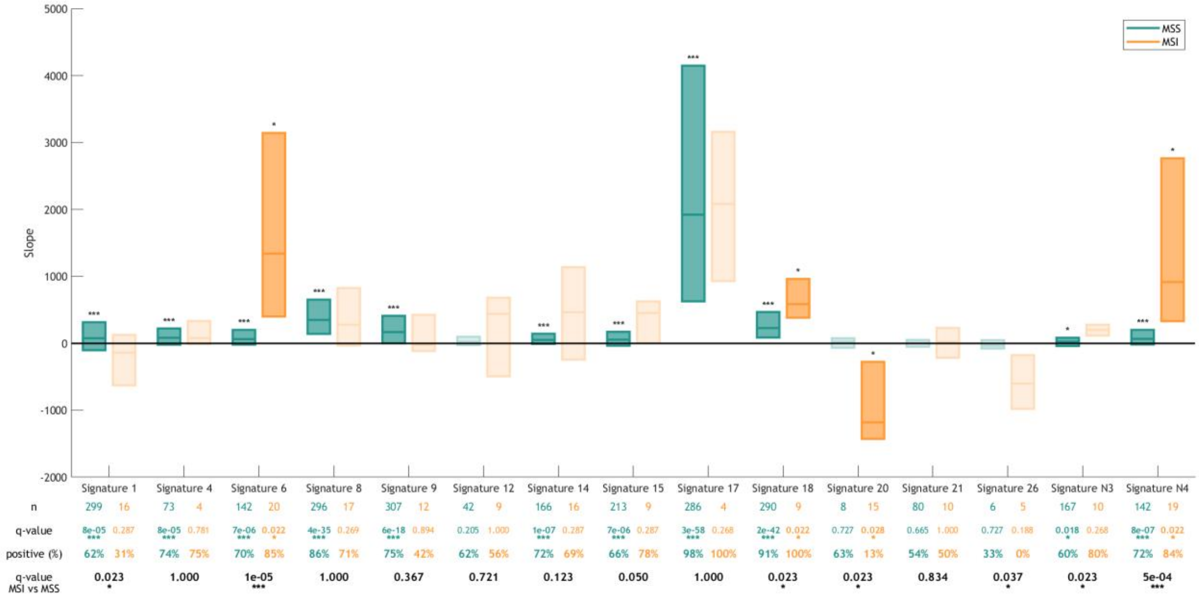

Fig. S26: Comparison of effects of replication timing on mutational signatures in MSI and MSS samples. The boxplots show distribution of slope of correlation of signature exposure with replication timing in individual patients, grouped by the MSI status. The analysis was performed on tissues with MSI samples: gastric, colorectum, oesophageal adenocarcinoma. The values below the signature names show number of samples (n), Benjamini-Hochberg corrected p-values (signtest of the slopes), the percentage of samples with a positive slope, and comparison of slopes in MSI vs MSS samples (ranksum test of slopes in the two groups; Benjamini-Hochberg corrected).

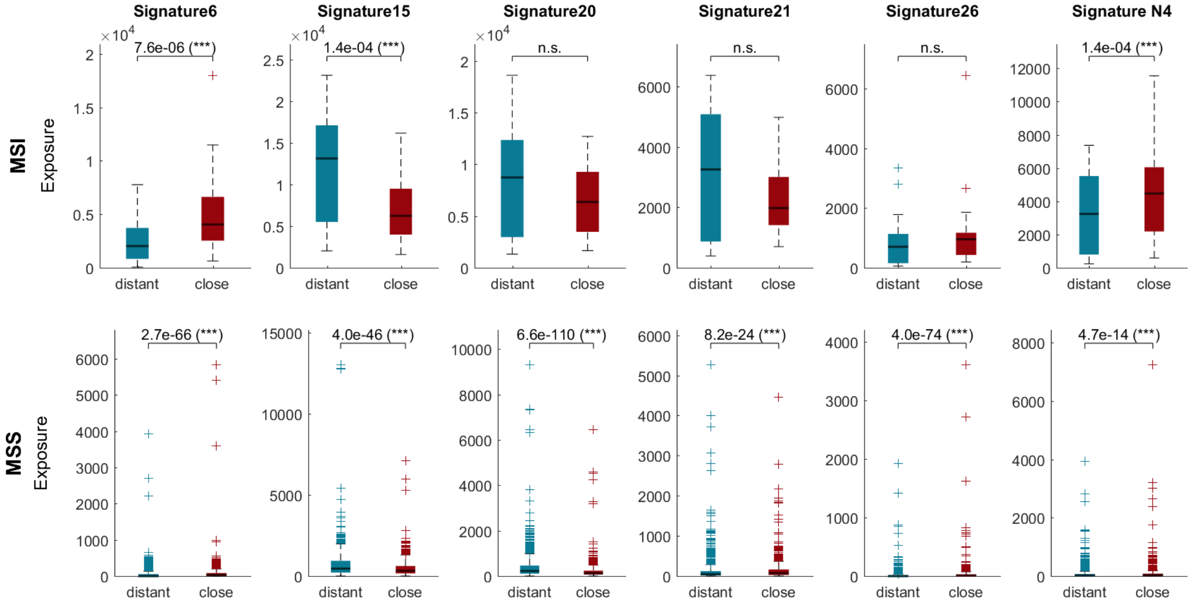

Fig. S27: MMR signatures 6 and N4 have increased exposures around ORIs both in MSI and MSS samples. Based on SNS-seq of ORI, exposures to signatures were compared in regions close to ORI (at most 250 kbp) and distant from ORI (between 500 kbp and 1 Mbp). The difference was evaluated with signtest, $p < 0.05$ was considered significant.

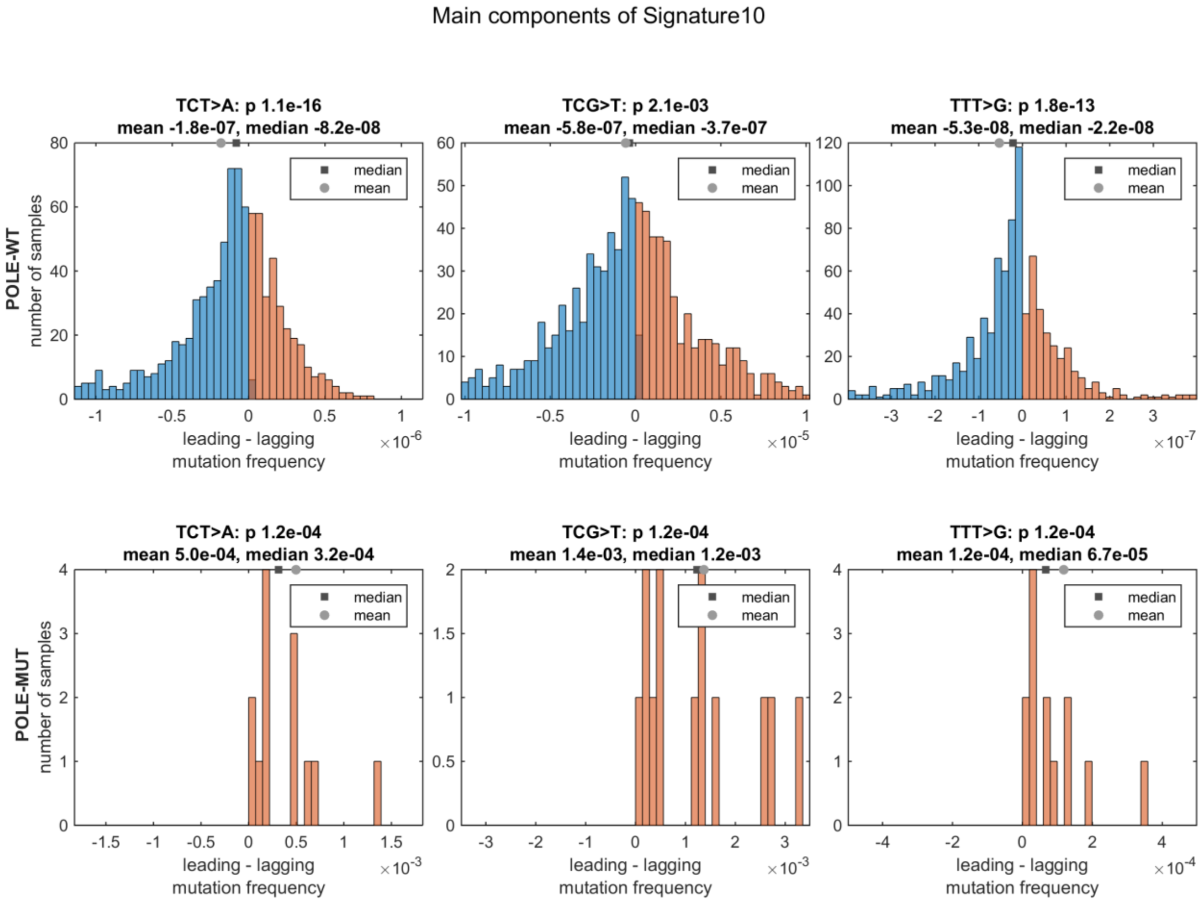

Fig. S28: Inverse exposure of signature 10 in POLE-MUT vs. POLE-WT samples. Frequency of mutations in TCT>A, TCG>T, and TTT>G, the three major components of signature 10, is higher on the lagging strand than on the leading strand in POLE-WT samples, whereas it is higher on the leading strand in POLE-MUT. Only samples exposed to signature 10 (exposure above 10) are shown. Signtest was used to evaluate the mutation frequency difference between the leading and lagging strands.

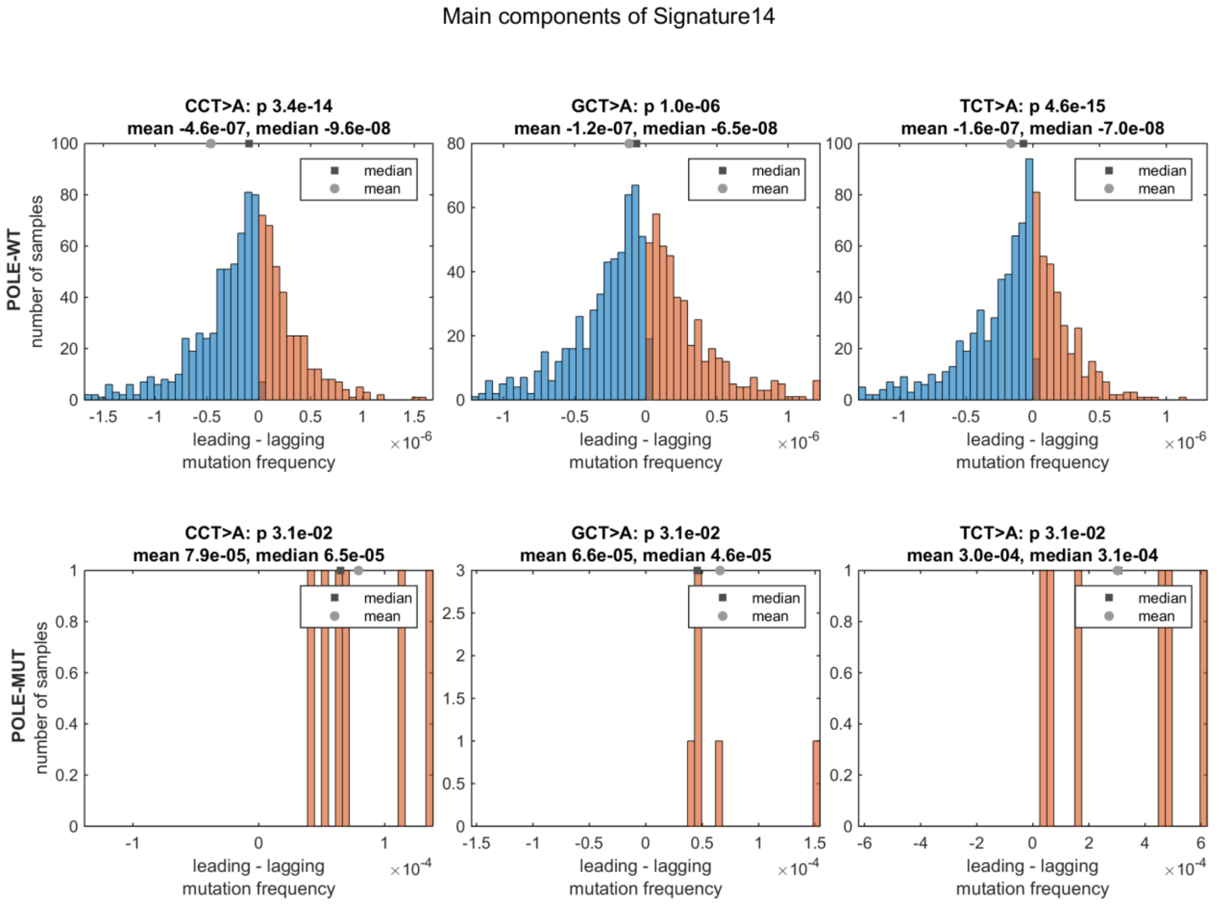

Fig. S29: Inverse exposure of signature 14 in POLE-MUT vs. POLE-WT samples. Frequency of mutations in CCT>A, GCT>A, and TCT>A, the three major components of signature 14, is higher on the lagging strand than on the leading strand in POLE-WT samples, whereas it is higher on the leading strand in POLE-MUT. Only samples exposed to signature 14 (exposure above 10) are shown. Signtest was used to evaluate the mutation frequency difference between the leading and lagging strands.

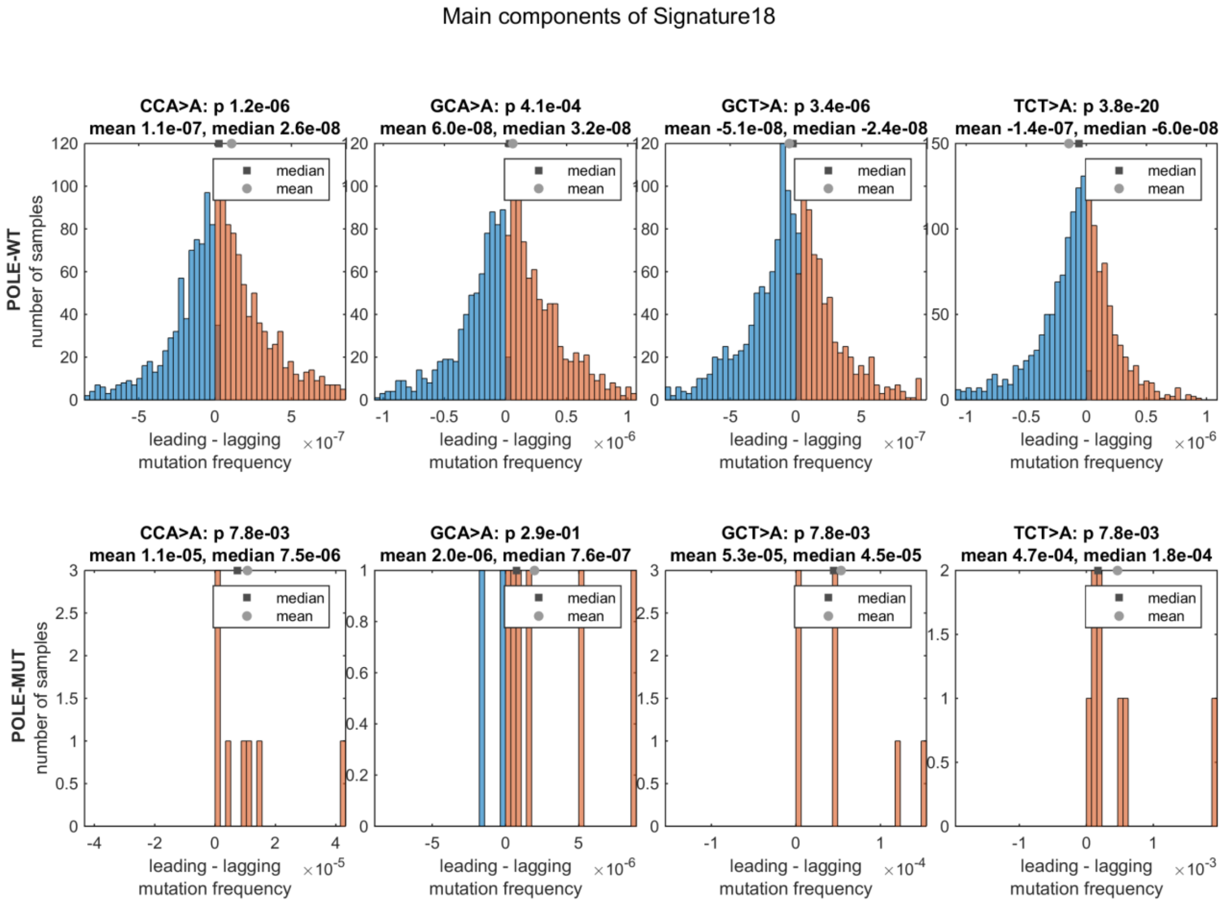

Fig. S30: Inverse exposure of signature 18 in POLE-MUT vs. POLE-WT samples. Frequency of mutations in CCA>A, GCA>A, GCT>A, and TCT>A, the four major components of signature 18, in POLE-WT and POLE-MUT. Only samples exposed to signature 18 (exposure above 10) are shown. Signtest was used to evaluate the mutation frequency difference between the leading and lagging strands.

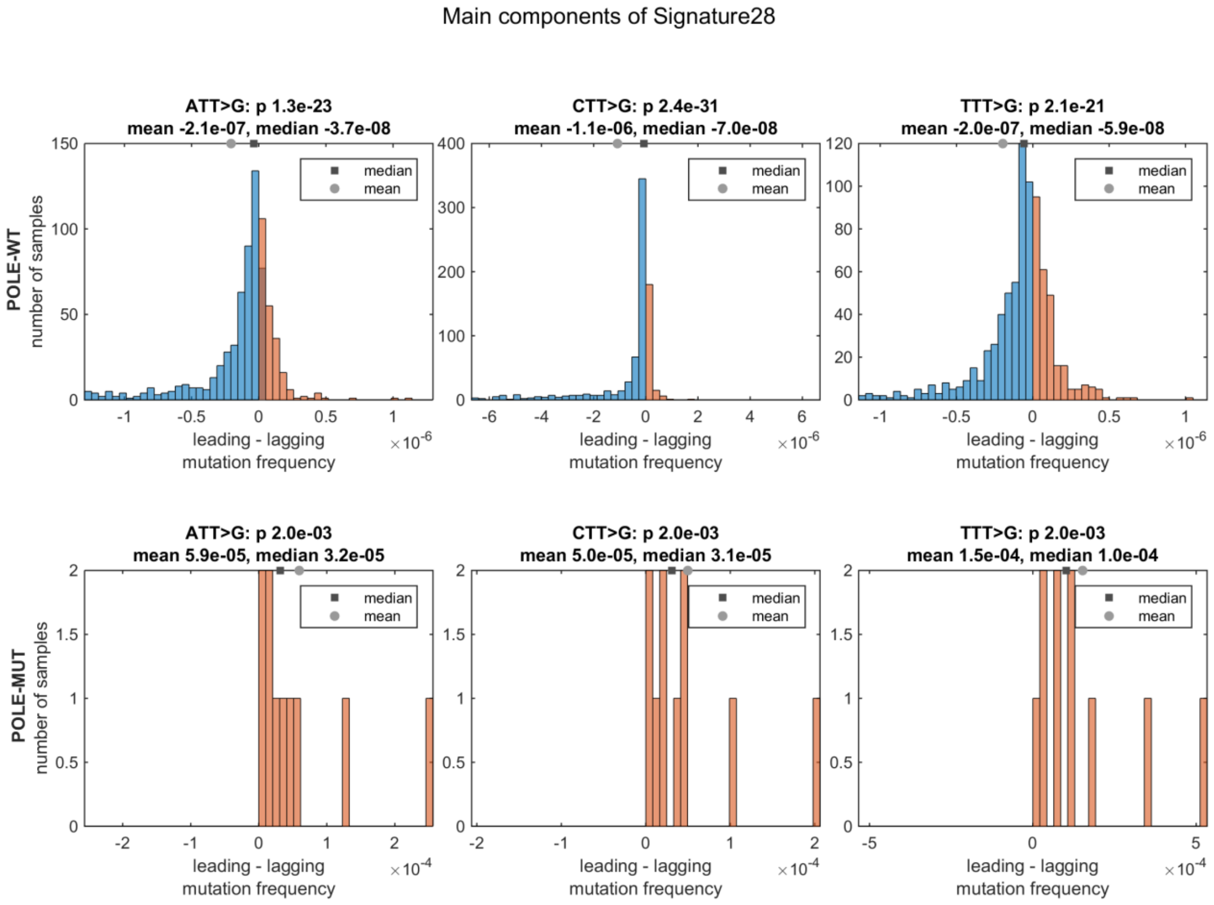

Fig. S31: Inverse exposure of signature 28 in POLE-MUT vs. POLE-WT samples. Frequency of mutations in ATT>G, CTT>G, and TTT>G, the three major components of signature 28, is higher on the lagging strand than on the leading strand in POLE-WT samples, whereas it is higher on the leading strand in POLE-MUT. Only samples exposed to signature 28 (exposure above 10) are shown. Signtest was used to evaluate the mutation frequency difference between the leading and lagging strands.

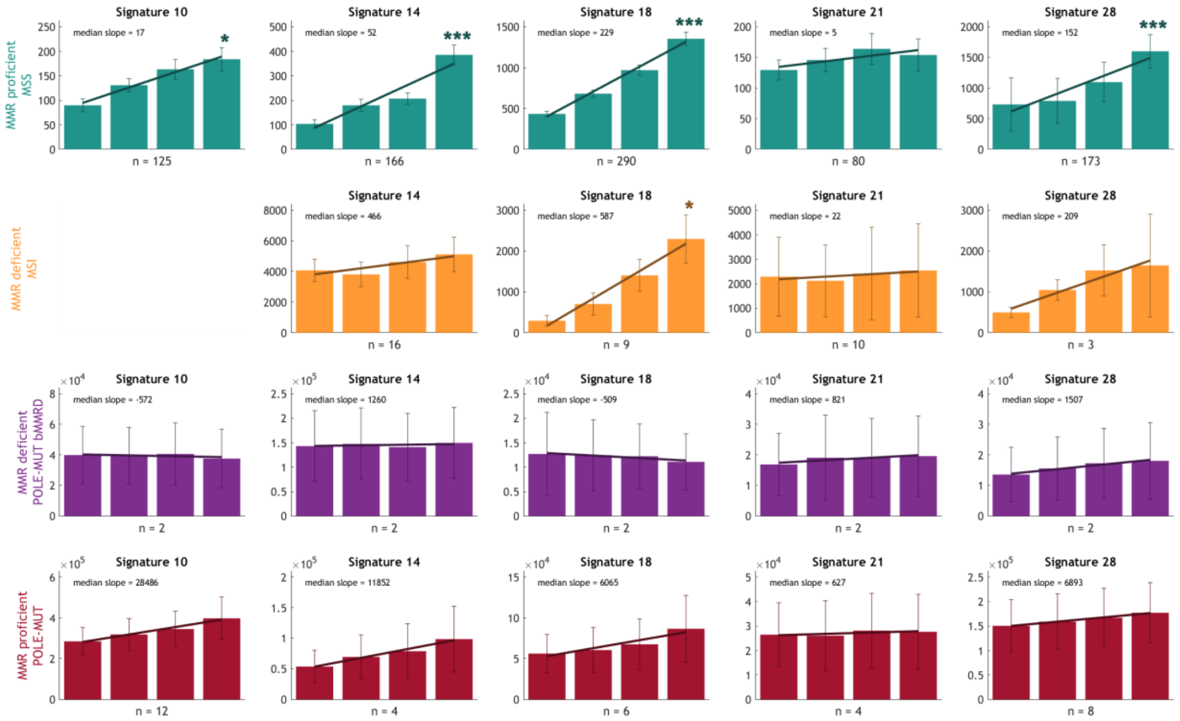

Fig. S32: Comparison of effects of replication timing on mutational signatures in MSI, MSS, POLE-MUT, and POLE-WT samples. Average exposure to mutational signatures with respect to replication timing quartiles: early (first bin) to late (last bin). Samples are grouped according to their MSI status: MMR proficient POLE-WT in green (first row), MMR deficient POLE-WT in orange (second row), MMR deficient POLE-MUT in purple (third row), and MMR proficient POLE-MUT in carmine red (fourth row). The error bars represent standard error of the mean. Signatures with exposure of at least 10 in at least 4 POLE-MUT samples were included. The asterisks denote signatures with significant effect of replication timing (signtest of the slopes, Benjamini-Hochberg corrected).

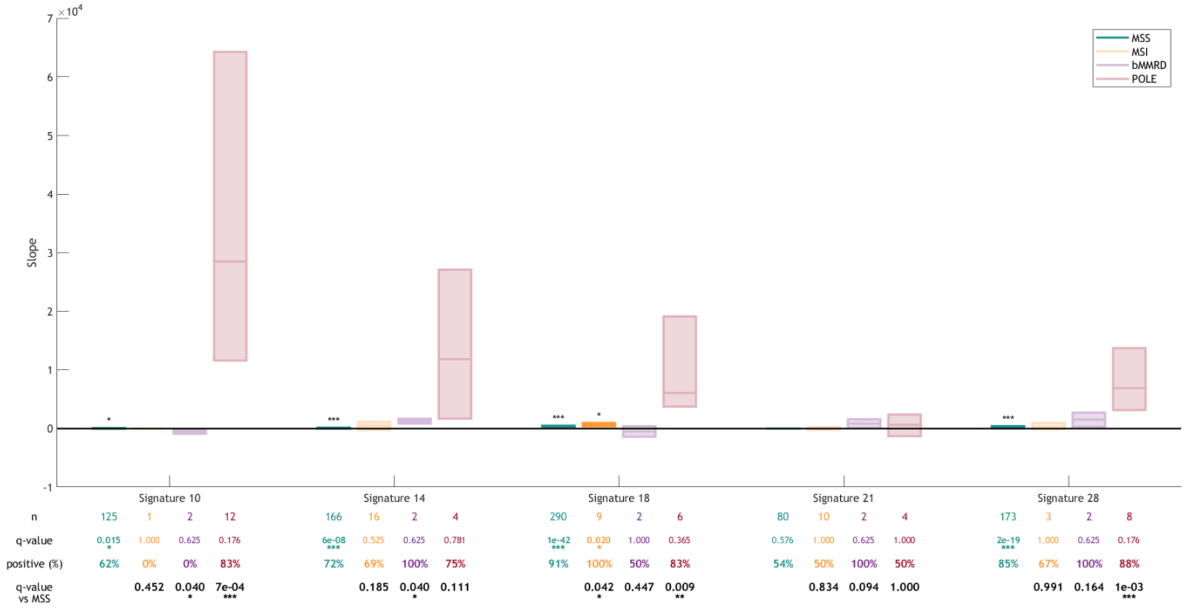

Fig. S33: Comparison of effects of replication timing on mutational signatures in MSS, MSI, and POLE-MUT samples. The boxplots show distribution of slope of correlation of signature exposure with replication timing in individual patients, grouped by the MSI and POLE-MUT status (bMMRD: biallelic mismatch repair deficient POLE-MUT samples). The values below the signature names show number of samples (n), Benjamini-Hochberg corrected p-values (signtest of the slopes), the percentage of samples with a positive slope, and comparison of slopes in MSI vs MSS samples (ranksum test of slopes in the two groups; Benjamini-Hochberg corrected).
