# Supplementary material for: Mutational signature distribution varies with DNA replication timing and strand asymmetry
Source: Genome Biol. 2018 Sep 10;19:129. doi: 10.1186/s13059-018-1509-y (PMC6130095; doi:10.1186/s13059-018-1509-y)
Supplement: Supplementary file 3 — Table S2. Overview of the strand asymmetry and correlation with replication timing in mutational signatures. (PDF 205 kb) [file 13059_2018_1509_MOESM3_ESM.pdf]

Table S2: Overview of the strand asymmetry and correlation with replication timing in mutational signatures.

	strand asymmetry		correlation with replication timing		
**signature**	**sample type**	**dominant mutation types**	**sample type**	**enriched in**	**effect of MMR**
1	all	NCG>NCT leading	all	late	slope decreased in MSI
2	all	TCN>TTN lagging	all	late	NA (not in MSI)
3	all	mixed	all	late	NA (not in MSI)
4	lung	C>A leading	all	late	n.s.
5	-	-	all	early	n.s.
6	MSI (n.s.)	C>T leading	all	late	slope increased in MSI
7	all	C>T lagging	all	late	NA (not in MSI)
8	all	C>A leading	all	late	n.s.
9	all	mixed	all	late	n.s.
10	all	POLE-MUT: leading, POLE-WT: lagging	all	late	NA (not in MSI)
12	all	T>C leading	all	late	n.s.
13	all	TCN>TGN lagging	all	late	NA (not in MSI)
14	all	POLE-MUT: leading, POLE-WT: lagging	all	late	n.s.
15	MSI (n.s.)	C>T leading	all	late	n.s.
16	all	mixed	all	early	NA (not in MSI)
17	all	T>N lagging	all	late	n.s.
18	all	mixed	all	late	slope increased in MSI
19	-	-	-	-	NA (not in MSI)
20	MSI	C>T leading, C>A and T>C lagging	all	early	slope decreased (negative) in MSI
21	all	C>T leading, T>C lagging	-	-	n.s.
22	kidney (n.s.)	T>A leading	all	late	NA (not in MSI)
23	all	C>T leading	-	-	NA (not in MSI)
25	all	mixed	-	-	NA (not in MSI)
26	MSI (n.s.)	C>T leading, C>A and T>C lagging	MSI (n.s.)	early	slope decreased in MSI
28	all	POLE-MUT: leading, POLE-WT: lagging	all	late	NA (not in MSI)
N1	all	mixed	all	late	NA (not in MSI)
N2	all	mixed	-	-	NA (not in MSI)
N3	all	mixed	all	late	slope increased in MSI
N4	all	mixed	all	late	slope increased in MSI
