# Supplementary material for: Mutational signature distribution varies with DNA replication timing and strand asymmetry
Source: Genome Biol. 2018 Sep 10;19:129. doi: 10.1186/s13059-018-1509-y (PMC6130095; doi:10.1186/s13059-018-1509-y)
Supplement: Supplementary file 7 — Review history. (DOCX 64 kb) [file 13059_2018_1509_MOESM7_ESM.docx]

**First round of review**

**Reviewer 1**

**Are the methods appropriate to the aims of the study, are they well described, and are necessary controls included?**
No - I have substantial methodological concerns, not helped by the poor description of the methods used. There are also key control analyses missing (e.g. the suggested random permutation on origins/replication domains).

**Are the conclusions adequately supported by the data shown?**

No - even if the data is correctly calculated, there is a conceptual problem that would undermine certainly the replication timing aspect of the results and similar arguments could be made for the strand-asymmetry.

We already have good evidence that the preferential targeting of missmatch repair (MMR) to early replicating regions explains much of the difference in mutation rate between early and late replication (Supek & Lehner, Nature 2015). Regardless of the source of the mutation or mutagenic lesion, MMR could be shaping the observed distribution of mutations, for example POLE proof-reading defective signatures (#10 and #7 in Fig 4) both show lower mutation rate near origins and a correlation of mutation rate across replication timing quartiles. However, this replication time effect could be that POLE adds its mutations uniformly and then biases in MMR differentially repair those mismatch lesions. This isn’t just a hypothetical, THIS IS the killer analysis from (Supek & Lehner, Nature 2015) that demonstrated differential MMR over replication timing. The same arguments can be applied to all the replication timing correlations. A single repair process with differential activity over different replication timing domains could potentially explain all replication-time correlations. That the magnitude differs between signatures could simply be the matching of the mutation signature of the underlying mutational process and the biases in repair of MMR (or any other temporally biased repair process). In summary, the differential repair activity has not been de convolved form the mutation signatures, it is already known to be an important replication-time-dependent shaper of mutation rate so could explain all such patterns.

**Are sufficient details provided to allow replication and comparison with related analyses that may have been performed?**

No - methods lack detail, main metric "exposure" is not defined, we know which studies the sequencing/mutation data came from but not which samples.

**Is the paper of broad interest to others in the field, or of outstanding interest to a broad audience of biologists?**

Yes: Very current and important field, right questions poor execution.

**Comments to authors**

In this manuscript, Tomkova et al set out to explore differences in point mutation signatures of human cancers relative to replication timing and leading versus lagging strand asymmetry. They have used publicly available whole genome sequencing data and adaptations of the approaches pioneered by Alexandrov et al to both identify signatures and de-convolve them into their weighted contributions. The novelty of this work is the integration with inferred origins of replication and to a lesser extent the partitioning of the genome by replication timing. The main finding is that most (76%) of the 29 mutation signatures considered show relationships with replication, a replication-strand (and) or temporal asymmetry in the mutation signature.

The association of replication timing, higher mutation rate in later replicating regions, has been well established (partly stemming from the senior author's 2012 Nature publication and follow-up). Here the authors are claiming to extend that to show different effects on distinct mutation signatures. The strand asymmetry, differential mutation depending on whether the mutational lesion/event occurs on the leading or lagging strand, has been demonstrated for a hand-full of processes such as APOBEC editing and proof-reading defects in replicative polymerases as the authors have cited, but the results presented in this manuscript argue that strand asymmetries are the rule rather than the exception.

This is a very interesting set of questions and work that is of the moment. A conceptual concern with regard to the replication timing is that we already have good evidence that the preferential targeting of missmatch repair (MMR) to early replicating regions explains much of the difference in mutation rate between early and late replication (Supek & Lehner, Nature 2015). Regardless of the source of the mutation or mutagenic lesion, MMR could be shaping the observed distribution of mutations, for example POLE proof-reading defective signatures (#10 and #7 in Fig 4) both show lower mutation rate near origins and a correlation of mutation rate across replication timing quartiles. However, this replication time effect could be POLE adds its mutations uniformly and then biases in MMR differentially repair those mismatch lesions. This isn't just a hypothetical, IT IS the killer analysis from (Supek & Lehner, Nature 2015) that showed differential MMR over replication timing. The same arguments can be applied to all the replication timing correlations. A single repair process with differential activity over different replication timing domains could potentially explain all temporal correlations. The magnitude differs between signatures could simply be the matching of the mutation signature of the underlying mutational process and the biases in repair of MMR (or any other temporally biassed repair process). In summary, the differential repair activity has not been de convolved form the mutation signatures, it is already known to be an important replication-time-dependent shaper of mutation rate so could explain all such patterns.


In addition to the concerns above I found the manuscript poorly described and lacking sufficient technical detail to assess the validity of these claims.

Major concerns
1. For figures 2, 3 and 4 (and most of the supplemental figures) we don't know what samples we are looking at. I Figures 3 and 4: How many? (can be approximately inferred from 4th column histograms), what distribution of cancer types? If the aggregation analyses (columns 1,2,3,5) are dominated by a small number of hyper-mutated patients with those signature or not. What window sizes are use for the column 2 and 3 analyses (is defined in methods, should be clear from the figure or its legend).

2. What are the units of the "exposure". This is a term introduced in this manuscript, is one of the main metrics this work is based on yet it is not defined. I infer that it something like the genome scaled normalised contribution of mutations for the defined signature but I should not be having to guess. Form the differences in scale it also looks like it is probably summed across patients for figures 2 and 3 but again who knows!

3. I'm not convinced the rate vectors (mutation signatures) have been correctly calculated. The methods state that "The remaining signatures were then normalized by the frequency of trinucleotides in the leading and lagging strand and subsequently multiplied by the frequency of trinucleotides in the genome." This suggests to me that the 192 value mutation count vectors were divided by the occurrence of each trinucleotide summed over the forward and reverse strand. This would not be correct as it would average the trinucleotide composition between strands and it is know that this asymmetric between leading and lagging strands (see for example Petryk et al, Nature Comms. 2016 and references there in). More generally, the manuscript lacks control analyses, for example randomly permuting the inferred origin sites to show a temporally flat and strand-symmetric mutation signature confirming that all steps of the analysis are performing correctly. Code and more details of exactly how analyses were performed need to be provided.

4. TCGA samples were mutation called using Strelka within the project. Such raw mutation calls are typically subject to stringent filtering followed by some form of validation, quality checking or cross-referencing. Somatic mutations are publicly available for the TCGA whole genomes and by virtue of being used by multiple groups the community collectively has some idea of problems and biases in such data.

Other issues to address
5. There should be a complete list of genome/patient identifiers so for each genome dataset used. The work cannot be reproduced without it.

6. Page 5, paragraph 2, the authors assert that there is "very strong correlation between replication timing data", should be at least quantified and ideally supplemental figure to illustrate.

**Reviewer 2**

**Are the methods appropriate to the aims of the study, are they well described, and are necessary controls included?**
Not always. See comments to the authors

**Are the conclusions adequately supported by the data shown?**

Yes, although some results could be better explained. See comments to the authors

**Are sufficient details provided to allow replication and comparison with related analyses that may have been performed?**

No. This is the weakest part of the study. See comments to the authors

**Does the work represent a significant advance over previously published studies? Please explain.**

The work is mostly descriptive. However, it offers novel insights in the role of replication in influencing cancer mutational signatures

**Is the paper of broad interest to others in the field, or of outstanding interest to a broad audience of biologists?**

Yes: Choice of statistical testing seems adequate.

Comments to authors:

The paper from Tomkova et al aims to quantify the contribution of replication to cancer mutational signatures. They find a replication bias in most of the mutational signatures andoffer extensive explanation of what the reasons for this could be.

The study is potentially interesting, albeit mostly descriptive. In my opinion the description of what the authors have done is often unclear and this needs improvements before the study can be properly evaluated. 

For example, the authors explain their method of assigning the strand specificity of mutational signatures in Figure 1. The approach is based on ref.11, 13, and 15. However, this reviewer fails to understand how the authors go from replication domains to strand. If this information is contained in ref [11], it should be made clear to the reader in the form of a figure or better explained in the methods of the m/s because it is important to understand a vital step of their approach. 

Similarly, the mutational signatures were derived using the code developed in another publication [Alexandrov et al]. Again, since the authors refer to several parameters (minimal error, maximal stability, number of signatures, etc), they should recapitulate the method that they have used. This improves reproducibility and avoids cross reference with previous work.

Also, in the conclusions the authors state that their study "produces testable hypotheses which we anticipate to be experimentally validated in the future" - it would be good to give some examples of these testable hypothesis and suggestions of how they could be tested.


SPECIFIC POINTS
In my opinion, the authors could do a better job in improving the description of the methods to make their study reproducible. Several points of what they have done lack the necessary level of information to reproduce the data (see below).

Also, reference to supplementary figures (28!, maybe they could be reduced/grouped) in the main text is not quite straightforward. 

- The UCSC CGHub has been dismissed and the authors should update the source of samples accordingly.

-p.12: "Signatures extracted in each cancer type and COSMIC signatures were all pooled together (with equal values in the leading and lagging part in the COSMIC signatures) and were clustered using unsupervised hierarchical clustering (with cosine distance and complete linkage. linkage). A threshold was selected to identify clusters of similar signatures." What threshold? If I were to reproduce these results what threshold should I use?

- what type of 'manual examination' was done? Maybe this could be reported in a supplementary figure or table.

- "For the subsequent analysis, the signatures were converted back to 96 values: the 25 previously observed signatures were used in their original form and average of the leading and lagging part were used for the 4 newly identified signatures." I do not understand what this means.

- "Each signature was annotated with strand direction: which of the 96 mutation types were higher on the leading strand and which on the lagging strand" - please revise English

- p.13: "the least exposed signature was incrementally removed (in both leading and lagging parts) while the resulting error did not exceed the original error by 0.5%." - when did this process stop?

- Legend of Figure 3 and 4 is oddly dived into 'columns'. Could this be converted into a more conventional division into panels?

Authors’ response to reviewers

Reviewer 1

*Reviewer #1: In this manuscript, Tomkova et al set out to explore differences in point mutation signatures of human cancers relative to replication timing and leading versus lagging strand asymmetry. They have used publicly available whole genome sequencing data and adaptations of the approaches pioneered by Alexandrov et al to both identify signatures and de-convolve them into their weighted contributions. The novelty of this work is the integration with inferred origins of replication and to a lesser extent the partitioning of the genome by replication timing. The main finding is that most  (76%) of the 29 mutation signatures considered show relationships with replication, a replication-strand (and) or temporal asymmetry in the mutation signature.*

*The association of replication timing, higher mutation rate in later replicating regions, has been well established (partly stemming from the senior author's 2012 Nature publication and follow-up). Here the authors are claiming to extend that to show different effects on distinct mutation signatures. The strand asymmetry, differential mutation depending on whether the mutational lesion/event occurs on the leading or lagging strand, has been demonstrated for a hand-full of processes such as APOBEC editing and proof-reading defects in replicative polymerases as the authors have cited, but the results presented in this manuscript argue that strand asymmetries are the rule rather than the exception.*

*This is a very interesting set of questions and work that is of the moment. A conceptual concern with regard to the replication timing is that we already have good evidence that the preferential targeting of missmatch repair (MMR) to early replicating regions explains much of the difference in mutation rate between early and late replication (Supek & Lehner, Nature 2015). Regardless of the source of the mutation or mutagenic lesion, MMR could be shaping the observed distribution of mutations, for example POLE proof-reading defective signatures (#10 and #7 in Fig 4) both show lower mutation rate near origins and a correlation of mutation rate across replication timing quartiles. However, this replication time effect could be POLE adds its mutations uniformly and then biases in MMR differentially repair those mismatch lesions. This isn't just a hypothetical, IT IS the killer analysis from (Supek & Lehner, Nature 2015) that showed differential MMR over replication timing. The same arguments can be applied to all the replication timing correlations. A single repair process with differential activity over different replication timing domains could potentially explain all temporal correlations. The magnitude differs between signatures could simply be the matching of the mutation signature of the underlying mutational process and the biases in repair of MMR (or any other temporally biassed repair process). In summary, the differential repair activity has not been de convolved form the mutation signatures, it is already known to be an important replication-time-dependent shaper of mutation rate so could explain all such patterns.*

**Each mutational signature is a result of a DNA damaging process and the subsequent repair. Since the major repair pathway for mutations introduced by Pol ε is MMR, it is very possible that MMR is shaping the profile of POLE-linked signatures. It is however unlikely that MMR is the cause for the enrichment of mutations in late-replicated regions in all signatures. In order to explore in which of the signatures MMR could be driving the correlation, we extended the analysis by comparing signature exposures in MSI and MSS samples (in those signatures that were present in at least 4 MSI samples). The results show that MSI samples have a significant correlation with replication timing in a number of MMR-related (6, 20, and N4) and MMR-unrelated (8, 17, and 18) signatures. Interestingly, the slope is steeper in MSI samples than MSS samples in signature 17 (log2 fold-change from early to late MSI: 12.4, MSS: 2.2) and signature 18 (log2 fold change MSI: 3.7, MSS: 1.3). These results show that MMR alone cannot explain temporal correlations in these signatures.**

**On the other hand, our analysis of POLE-M samples with and without biallelic mismatch repair deficiency shows that the correlation stays significant in MMR-deficient samples only in signature 28, but is lost in signatures 10, 14, and 18, suggesting that MMR can be the cause of the correlation in these three signatures.**

**Of note, signature #7 is not associated with POLE proof-reading defects, but linked to UV light exposure. We do not have any WGS MSI samples with an exposure to signature 7, so we could not repeat the analysis from above. However, the main pathway responsible for the repair of UV-induced damage is nucleotide excision repair (NER), not MMR. Both differential activity of NER in early vs. late-replicated regions (possibly because of the correlation with differential accessibility) and differential usage of DNA damage tolerance pathways could explain the steep correlation with replication in signature 7 and other signatures associated with DNA damage caused by external mutagens.**

*In addition to the concerns above I found the manuscript poorly described and lacking sufficient technical detail to assess the validity of these claims.*

*Major concerns*

*1. For figures 2, 3 and 4 (and most of the supplemental figures) we don't know what samples we are looking at. I Figures 3 and 4: How many? (can be approximately inferred from 4th column histograms), what distribution of cancer types?*

**We have added a pie-chart of the proportional contributions by individual tissue types (number of samples with a given signature, weighted by their exposure, to best represent the contributions in the plots with average exposure) and the total number of samples in the plot.**

*If the aggregation analyses (columns 1,2,3,5) are dominated by a small number of hyper-mutated patients with those signature or not.*

**It is true that the plots with averaged values (columns 2, 3, and 5) could be influenced by outlier hyper-mutated samples. We have therefore changed the visualisation by removing any potential outlier values first (using Tukey's fences with k = 2). Moreover, we have plotted standard errors of the mean in the form of error-bars in the column 5.**

*What window sizes are use for the column 2 and 3 analyses (is defined in methods, should be clear from the figure or its legend).*

**We have added this information into the legend.**

*2. What are the units of the "exposure". This is a term introduced in this manuscript, is one of the main metrics this work is based on yet it is not defined. I infer that it something like the genome scaled normalised contribution of mutations for the defined signature but I should not be having to guess. Form the differences in scale it also looks like it is probably summed across patients for figures 2 and 3 but again who knows!*

**The general term of “exposure” to a signature is not introduced by us, but already used since the first mutational signatures paper** (Alexandrov, Nik-Zainal, Wedge, Campbell, & Stratton, 2013)**. We have used the description suggested by the reviewer, as it well describes the metric used in our method.**

**Figures 2 and 3 (columns 2, 3, and 5) show the average values across patients. We have changed the y-axis to “average exposure”.**

*3. I'm not convinced the rate vectors (mutation signatures) have been correctly calculated. The methods state that "The remaining signatures were then normalized by the frequency of trinucleotides in the leading and lagging strand and subsequently multiplied by the frequency of trinucleotides in the genome." This suggests to me that the 192 value mutation count vectors were divided by the occurrence of each trinucleotide summed over the forward and reverse strand. This would not be correct as it would average the trinucleotide composition between strands and it is know that this asymmetric between leading and lagging strands (see for example Petryk et al, Nature Comms. 2016 and references there in). More generally, the manuscript lacks control analyses, for example randomly permuting the inferred origin sites to show a temporally flat and strand-symmetric mutation signature confirming that all steps of the analysis are performing correctly. Code and more details of exactly how analyses were performed need to be provided.*

**The fact that the composition of trinucleotides between strands is to some extent asymmetric is exactly one of the reasons why the mutation counts need to be corrected for the trinucleotide frequency. If the correction was not performed, a signature could have more mutations on one of the strands simply because there are more positions of that trinucleotide on that strand where the mutations could have happened. This normalization is therefore performed both in the step of extracting signatures and computing exposures to the signatures. This is important for the correction of differences in trinucleotide frequency between the two strands, but also for differences between the regions that were taken into account in the given analysis.**

**We have added the control analysis with random origins and randomized replication timing, which shows that all signatures are strand-symmetric and temporally flat (Fig. 2–figure supplement 3–4).**

**The code is available at https://bitbucket.org/bsblabludwig/replicationasymmetry.**

*4. TCGA samples were mutation called using Strelka within the project. Such raw mutation calls are typically subject to stringent filtering followed by some form of validation, quality checking or cross-referencing. Somatic mutations are publicly available for the TCGA whole genomes and by virtue of being used by multiple groups the community collectively has some idea of problems and biases in such data.*

**We are not aware of any publicly available variant calls for the TCGA whole genomes, as we found only WXS calls at the official https://portal.gdc.cancer.gov/ portal. It should be noted that only a subset of genomes used in this study was processed by ourselves. The majority of somatic mutation data were taken from public sources, so any bias introduced by our variant calling would be limited.**

*Other issues to address*

*5. There should be a complete list of genome/patient identifiers so for each genome dataset used. The work cannot be reproduced without it.*

**We have added the list of all samples and their identifiers as an additional supplementary table 2.**

*6. Page 5, paragraph 2, the authors assert that there is "very strong correlation between replication timing data", should be at least quantified and ideally supplemental figure to illustrate.*

**This sentence was referring to Fig. 2–figure supplement 5–7, which show how the log_2_ fold-change (from early to late) of exposure values correlate when using the different genomic regions. However, as we have now realized that this sentence could be confusing for the reader, we have removed it from the updated manuscript.**

Reviewer 2

*Reviewer #2: The paper from Tomkova et al aims to quantify the contribution of replication to cancer mutational signatures. They find a replication bias in most of the mutational signatures and offer extensive explanation of what the reasons for this could be.*

*The study is potentially interesting, albeit mostly descriptive. In my opinion the description of what the authors have done is often unclear and this needs improvements before the study can be properly evaluated.*

*For example, the authors explain their method of assigning the strand specificity of mutational signatures in Figure 1. The approach is based on ref.11, 13, and 15. However, this reviewer fails to understand how the authors go from replication domains to strand. If this information is contained in ref [11], it should be made clear to the reader in the form of a figure or better explained in the methods of the m/s because it is important to understand a vital step of their approach.*

**We have extended the methods with a summary of how left- and right-replicating domains and leading and lagging strands were defined in ref. 11.**

*Similarly, the mutational signatures were derived using the code developed in another publication [Alexandrov et al]. Again, since the authors refer to several parameters (minimal error, maximal stability, number of signatures, etc), they should recapitulate the method that they have used. This improves reproducibility and avoids cross reference with previous work.*

**We have added more technical details of the extraction of signatures introduced by Alexandrov et al. to the methods.**

*Also, in the conclusions the authors state that their study "produces testable hypotheses which we anticipate to be experimentally validated in the future" - it would be good to give some examples of these testable hypothesis and suggestions of how they could be tested.*

**We have added several examples, which could be tested by a combination of *in vitro* and *in vivo* models (using exposure to the given mutagen, measuring resulting mutations, possibly using one of the recent ultra-sensitive techniques, such as Maximum Depth Sequencing). Similarly, hypotheses about polymerases (Pol ε, Pol α, Pol η) can be tested *in vitro* using MDS.**

*SPECIFIC POINTS*

*In my opinion, the authors could do a better job in improving the description of the methods to make their study reproducible. Several points of what they have done lack the necessary level of information to reproduce the data (see below).*

*Also, reference to supplementary figures (28!, maybe they could be reduced/grouped) in the main text is not quite straightforward.*

**We have grouped six supplementary figures of Fig. 2 and reduced the number of supplementary figures of Fig. 1 by reorganizing the plots. We are of the opinion that the other supplementary figures would be difficult to read if more of them were grouped together. At the same time, we think that reducing their number would not be appropriate, as it would mean to hide results from a selected set of signatures.**

*- The UCSC CGHub has been dismissed and the authors should update the source of samples accordingly.*

**We have updated the source (Genomic Data Commons Data Portal).**

*-p.12: "Signatures extracted in each cancer type and COSMIC signatures were all pooled together (with equal values in the leading and lagging part in the COSMIC signatures) and were clustered using unsupervised hierarchical clustering (with cosine distance and complete linkage. linkage). A threshold was selected to identify clusters of similar signatures." What threshold? If I were to reproduce these results what threshold should I use?*

**A threshold of 27 signatures was used, as it best corresponded to the previously identified COSMIC signatures. We have updated the relevant part of the text in the methods.**

*- what type of 'manual examination' was done? Maybe this could be reported in a supplementary figure or table.*

**We have added a new Fig. 1–figure supplement 6 describing the four operations we have done: joining two clusters corresponding to signature 1 (in a similar way how the signature 1A and 1B were joined into the COSMIC signature 1), joining two clusters corresponding to signature 10, splitting one cluster into clusters corresponding to signature 6, signature 14, and signature 15, and finally splitting one cluster into signature 12 and N4.**

*- "For the subsequent analysis, the signatures were converted back to 96 values: the 25 previously observed signatures were used in their original form and average of the leading and lagging part were used for the 4 newly identified signatures." I do not understand what this means.*

**We have extended this sentence. The 25 COSMIC signatures were used in the exactly same form as how they can be downloaded from the COSMIC website (http://cancer.sanger.ac.uk/cancergenome/assets/signatures_probabilities.txt). For the 4 novel signatures, we converted the 192-elements-long strand specific signatures (containing leading part and lagging part) into 96-elements-long signatures by averaging the leading and lagging parts.**

*- "Each signature was annotated with strand direction: which of the 96 mutation types were higher on the leading strand and which on the lagging strand" - please revise English*

**We have rewritten the sentence as “Each signature was annotated with the dominant strand direction (leading vs lagging) in each of the 96 mutation types.” In other words, each mutation type in the signature was annotated with a boolean value, according to whether it was higher on the leading or lagging direction in the 192-elements-long strand specific signatures.**

*- p.13: "the least exposed signature was incrementally removed (in both leading and lagging parts) while the resulting error did not exceed the original error by 0.5%." - when did this process stop?*

**It stopped right before the error exceeded 0.5%.**

*- Legend of Figure 3 and 4 is oddly dived into 'columns'. Could this be converted into a more conventional division into panels?*

**We have added labels of the column (panel) indices into the figure.**

**Second round of review**

**Reviewer 1**

**Are the methods appropriate to the aims of the study, are they well described, and are necessary controls included?**
Generally yes. F-tests aren't justified (need to test assumption of normality) and don't appear to have been subject to multiple testing correction.

**Are the conclusions adequately supported by the data shown?**

Yes, although some results could be better explained. See comments to the authors No.

See main review comments.

**Are sufficient details provided to allow replication and comparison with related analyses that may have been performed?**

Yes but is perhaps not a clearly described as it could be.

**Does the work represent a significant advance over previously published studies? Please explain.**

Yes, it's interesting and well thought out work addressing important and current questions. However, I am not convinced the data presented supports the main conclusion. There is a more trivial explanation for the apparent biases that is not adequately excluded.

**Is the paper of broad interest to others in the field, or of outstanding interest to a broad audience of biologists?**

Yes: Of interest to cancer biology, evolution and the biology of DNA replication.

Comments to author:

Thinking of the question what would I cite this paper for - what specific point does it demonstrate that has not previously been demonstrated? The most appropriate phrase would be "The relationship between replication timing and leading/lagging strand synthesis with mutation signatures might be more complicated than previously thought".

My major concern with the initial submission of this work was that the replication timing and preferential strand targeting of a single (or multiple) repair processes could, in fact would be expected to, impose replication time and strand asymmetries on to patterns even if they were originally symmetric and uniform. The main assertion of this work is that most of the processes generating each of these mutation signatures are intrinsically non-uniform over replication time and/or they are replication strand biased. Mismatch repair (MMR) is the obvious, though not only candidate as it has already been shown to be preferentially targeted to early replicating regions and shows replication strand asymmetries. Seeking to address this, the authors have investigated the timing and strand biases of signatures in both MMR competent and MMR deficient cancers. This is absolutely the right thing to do but is inevitably limited by the number of MMR deficient cancer sequences available and the signatures that can be deconvolved from their overall mutation profile. For replication time this is well summarised in Fig. 4 - figure supplement 11. Despite a smattering of *'s through the orange bar-plots, very few (possibly only signature 18) show a convincing replication time correlation in the MMR deficient cancers. Although signatures 6, 8 and N4 all additionally report a significant correlation these are based on F-tests (without demonstrating the assumed normality of sample estimates) reporting p<0.05 without apparent multiple testing correction and standard error of the mean estimates showing wide and often overlapping intervals. Even based on these nominal p-value thresholds only 4/11 of the signatures showing replication time correlation in MMR competent cancers show evidence of that correlation in MMR deficient and of those 4 signatures 6, 8 and probably N4 are less than convincing. I would argue that the replication timing correlation is generally lost (or at least not shown here to be maintained) across most of the signatures. Turning to replication strand asymmetries, this data is less well collated and only apparently shown for MMR associated signatures (Fig. 4 - supplement 1) which do clearly show strand asymmetries that have previously been well established as signatures of MMR deficiency. In summary for this section, I an not convinced that my principal concern has been addressed despite the analyses presented. We could still be predominantly looking at MMR biases superimposed on other signatures.

This paper remains a very difficult to read. The methodological descriptions are improved but what has been done will still not be readily grasped by any but the most diligent reader. Further expansion of the Figure 1 schematic, for example illustrating the trivial flipping of histogram bars to point down rather than up for a change occurring predominantly on the lagging strand template (but not expressing how biased that specific change is between strands). Also help in the interpretation of columns 4 and 5 of Figure 3 (and similar figures) may aid interpretation.

I am now satisfied that the strand asymmetries and composition corrections are appropriately calculated and Figure 2 supplements 3 and 4 are a useful addition.

A summary table (in the main paper) would be very helpful in bringing together the desperate data. It would report on each mutation signature (1-28, N1, N2, N3, N4), what if any it's strand bias and what if any it's replication timing bias, and how both of these biases are influenced by missmatch repair competence.

**Reviewer 3**

**Are the methods appropriate to the aims of the study, are they well described, and are necessary controls included?**
Some details missing, see below

**Are the conclusions adequately supported by the data shown?**

Yes

**Are sufficient details provided to allow replication and comparison with related analyses that may have been performed?**

Some details missing, see below

**Does the work represent a significant advance over previously published studies? Please explain.**

Yes, in the context of mutational signatures together with the replication direction and timing. The signatures analysed here are comprehensive and the findings provide novel insights about the mutational processes in the context of replication.

**Is the paper of broad interest to others in the field, or of outstanding interest to a broad audience of biologists?**

Yes: Yes, the choice of statistical approach and the novel biological insights of mutational processes.

Comments to authors:

Tomkova et al analyses the replication strand bias and correlation of mutation rate with replication timing for a wide range of signatures. They find that there is an influence of replication in the mutation patterns for most of the signatures (24 out of 29). The analysis is sound, results are interesting and provide novel insights into the body of knowledge in the field.

The authors addressed satisfactorily the comments from reviewer #2.

There are several issues that should be improved or clarified before the paper is published.


How do authors explain signature 13 has a correlation with replication timing only for inverse but not for matching exposure (Fig. 3, column 5)?


Page 7: "Two mutational signatures (10 and 14) have been associated with Pol E, the main leading strand polymerase [23,27]".

The references to support the association between Signature 14 and POLE is missing here. Also, in this context, it needs to be clarified that the signature 10 is associated with POLE "mutants" (based on the previous studies, for example, ref. 12).

Note that a recent study by Andrianova et al., Genome Research, 2017 (DOI: 10.1101/gr.219915.116) has shown that the signature 10 is evident in POLE mutant samples with intact mismatch repair, whereas signature 14 is in POLE mutant samples with deficient mismatch repair. 

With respect to the above studies, how confident are the authors that the estimates of Signature 10 exposure (or mutations) observed in POLE WT samples are really from that signature (Figure 4)? Could that be a spurious/wrong association? Would be good that authors at least comment on that possibility.


Page 4: Authors should explain how are the MSI and POLE samples identified. The details are missing in the methods section.


Page 6: "Some MMR signatures in MMR deficient patients do not significantly correlate with replication timing (sig. 15, 21, 26) or do so only in one direction of replication (such as a negative correlation in the leading direction in sig. 20), whereas others show a clear increase in the late replicated regions (sig. 6 and N4, Fig. 4-figure supplement 1)."


Many MSI tumors do not have a complete impairment of MMR, and also the MMR deficiency could have been acquired late in the tumorigenic process, so that a number of mutations may have been accumulated in those tumors before the MMR deficiency. This could explain, at least partly, that there is correlation with replication timing in MSI and still be related to MMR. Are these signatures present in bMMRD tumors? If so, that would give a more definitive answer to the correlation of the replication timing of this signatures with MMR. If bMMRD samples do not show mutations on these signatures, at least authors show acknowledge that this is a limitation of the study that prevents a final conclusion.


Comparing the mutation pattern in MSI and MSS tumors (Fig. 4-figure supplement 1 and Fig. 4-figure supplement 2) several signatures seem to increase their replication strand bias in MSI tumors (eg. 20 and 21). Did the authors notice that? Do they have any explanation for that?


Page 13: Methods, exclude regions: which specific mappability track (or k-mer) has been used from the UCSC genome browser?


Page 15: "In order to minimize the number of spurious signature exposures, the least exposed signature was incrementally removed (in both leading and lagging parts) while as long as the resulting error did not exceed the original error by 0.5%."

What is the rationale for the choice of 0.5%? Is the choice based on the ref. 69 where the authors defined a similar threshold based on randomly generated tumors?


Would be useful that the authors provide the values of 29 strand-specific signatures, and the sample-wise exposure (matching and inverse) as a supplementary table. 


Also, a supplementary table with the details of the data points to reproduce figure 2 would be useful.


In figures related to signatures (e.g., Fig 3 and 4), specify what is the scale of the y-axis and the meaning of star symbol (on column 1). 


OTHER COMMENTS:

In discussion the authors say:
Our results also add a new perspective to the recent debate regarding the correlation of tissue-specific cell division rates with cancer risk [3]. It has been argued that this correlation is primarily attributable to "bad luck" in the form of random errors that are introduced during replication by DNA polymerases. However, the range of mutational signatures observed in cancer samples makes a purely replication-driven etiology of cancer mutations unlikely [62,63].

I do not see how the results presented in this paper do add any new perspective on this debate. The fact that "the range of mutational signatures observed in cancer samples makes a purely replication-driven etiology of cancer mutations unlikely" doesn't need the results of the present paper.


The scale median asymmetry in figure 2A (matching exposure - invese exposure) and in column 4 of figure 3 and 4 is misleading, as higher values don't mean higher asymmetry. Higher values can be achieved for those signatures with higher number of mutations. Would it be possible to find a value that is more meaningful of asymmetry itself, somehow normalized by the total number of mutations?


The list of authors in this citation are incorrect.
62. Gao Z, Wyman MJ, Sella G, Przeworski M, Campbell P, Nik-Zainal S. Interpreting the Dependence of Mutation Rates on Age and Time. Barton NH, editor. PLOS Biol. [Internet]. Public Library of Science; 2016 [cited 2017 Mar 10];14:e1002355. Available from: <http://dx.plos.org/10.1371/journal.pbio.1002355>

Authors’ response to reviewers

Reviewer 1

Thinking of the question what would I cite this paper for - what specific point does it demonstrate that has not previously been demonstrated? The most appropriate phrase would be "The relationship between replication timing and leading/lagging strand synthesis with mutation signatures might be more complicated than previously thought".

My major concern with the initial submission of this work was that the replication timing and preferential strand targeting of a single (or multiple) repair processes could, in fact would be expected to, impose replication time and strand asymmetries on to patterns even if they were originally symmetric and uniform. The main assertion of this work is that most of the processes generating each of these mutation signatures are intrinsically non-uniform over replication time and/or they are replication strand biased. Mismatch repair (MMR) is the obvious, though not only candidate as it has already been shown to be preferentially targeted to early replicating regions and shows replication strand asymmetries. Seeking to address this, the authors have investigated the timing and strand biases of signatures in both MMR competent and MMR deficient cancers. This is absolutely the right thing to do but is inevitably limited by the number of MMR deficient cancer sequences available and the signatures that can be deconvolved from their overall mutation profile. For replication time this is well summarised in Fig. 4 - figure supplement 11. Despite a smattering of *'s through the orange bar-plots, very few (possibly only signature 18) show a convincing replication time correlation in the MMR deficient cancers. Although signatures 6, 8 and N4 all additionally report a significant correlation these are based on F-tests (without demonstrating the assumed normality of sample estimates) reporting p<0.05 without apparent multiple testing correction and standard error of the mean estimates showing wide and often overlapping intervals. Even based on these nominal p-value thresholds only 4/11 of the signatures showing replication time correlation in MMR competent cancers show evidence of that correlation in MMR deficient and of those 4 signatures 6, 8 and probably N4 are less than convincing. I would argue that the replication timing correlation is generally lost (or at least not shown here to be maintained) across most of the signatures. Turning to replication strand asymmetries, this data is less well collated and only apparently shown for MMR associated signatures (Fig. 4 - supplement 1) which do clearly show strand asymmetries that have previously been well established as signatures of MMR deficiency. In summary for this section, I am not convinced that my principal concern has been addressed despite the analyses presented. We could still be predominantly looking at MMR biases superimposed on other signatures.

*We thank you for pointing out the missing multiple testing correction in Fig. 4-supplement 11 and validation of normality in the F-tests. In order to avoid usage of a parametric test, we have changed the analysis. We now calculate the correlation between replication timing and mutation rate per sample, and test if the median of the correlations deviates from 0 using a signtest. The resulting p-values were corrected for multiple testing using the Benjamini-Hochberg method. We have also added a comparison of slopes in MSS vs MSI samples. The updated analysis shows that signatures 6, 18, and N4 are significantly positively correlated with replication timing in both MSS and MSI samples, and signature 20 is significantly negatively correlated with replication timing in MSI samples. Moreover, the slope of the correlation is steeper in all these four signatures in MSI samples than MSS samples. In conclusion, although MMR is likely to contribute to the correlation with replication timing in some signatures (such as sig. 1, 10, 14, or 18), the results indicate that in other signatures MMR is not the only relevant factor.*

This paper remains a very difficult to read. The methodological descriptions are improved but what has been done will still not be readily grasped by any but the most diligent reader. Further expansion of the Figure 1 schematic, for example illustrating the trivial flipping of histogram bars to point down rather than up for a change occurring predominantly on the lagging strand template (but not expressing how biased that specific change is between strands). Also help in the interpretation of columns 4 and 5 of Figure 3 (and similar figures) may aid interpretation.

*We have expanded Figure 1, adding more details about the steps, and included examples explaining the graphs used in columns 4 and 5 in Figures 3 and 4.*

I am now satisfied that the strand asymmetries and composition corrections are appropriately calculated and Figure 2 supplements 3 and 4 are a useful addition.

A summary table (in the main paper) would be very helpful in bringing together the desperate data. It would report on each mutation signature (1-28, N1, N2, N3, N4), what if any it's strand bias and what if any it's replication timing bias, and how both of these biases are influenced by missmatch repair competence.

*We have added the summary of results for individual signatures as Table 1.*

Reviewer 2

Tomkova et al analyses the replication strand bias and correlation of mutation rate with replication timing for a wide range of signatures. They find that there is an influence of replication in the mutation patterns for most of the signatures (24 out of 29). The analysis is sound, results are interesting and provide novel insights into the body of knowledge in the field.

The authors addressed satisfactorily the comments from reviewer #2.

There are several issues that should be improved or clarified before the paper is published.

How do authors explain signature 13 has a correlation with replication timing only for inverse but not for matching exposure (Fig. 3, column 5)?

*APOBEC-induced deamination of C to U, the suggested mechanism behind sig 13, is thought to occur more frequently on the single-stranded template for the lagging strand. It has been hypothesized that the lack of replication-timing correlation for signature 13 is because mutations on the lagging strand template prevent MMR from working, since the template is needed to correct the error (Haradhvala et al., 2016). In contrast, the correlation of signature 13 with replication timing on the leading strand template could be the result of APOBEC acting during processes other than lagging strand replication. For example, single-stranded DNA during double-strand break repair has been suggested to be a substrate for APOBEC (Roberts et al., 2012), and might be slightly enriched in late-replicating regions.*

Page 7: "Two mutational signatures (10 and 14) have been associated with Pol E, the main leading strand polymerase [23,27]". The references to support the association between Signature 14 and POLE is missing here.

*Thank you for pointing out the missing reference. We have realized that the association between POLE and signature 14 was not described before, although it is relatively obvious from the studies which observed the mutations characteristic for signature 14 (when knowing the data on which the studies are based on) (Alexandrov et al., 2013; Shlien et al., 2015). We have therefore clarified this in the updated manuscript.*

Also, in this context, it needs to be clarified that the signature 10 is associated with POLE "mutants" (based on the previous studies, for example, ref. 12).

*We have clarified this in the updated manuscript.*

Note that a recent study by Andrianova et al., Genome Research, 2017 (DOI: 10.1101/gr.219915.116) has shown that the signature 10 is evident in POLE mutant samples with intact mismatch repair, whereas signature 14 is in POLE mutant samples with deficient mismatch repair.

*We have included this reference (who in fact explored the link between signature 14 and POLE based on our mention in the version of this manuscript on bioRxiv) into the updated manuscript.*

With respect to the above studies, how confident are the authors that the estimates of Signature 10 exposure (or mutations) observed in POLE WT samples are really from that signature (Figure 4)? Could that be a spurious/wrong association? Would be good that authors at least comment on that possibility.

*We in fact already tried to explore this possibility in the last version of this manuscript, by looking at the frequencies of the major mutation types of the signatures (e.g., TCT>TAT, TCG>TTG, and TTT>TGT in signature 10), i.e., independently of the signatures analysis. This approached confirmed the conclusions.*

*Now, we added one additional complementary approach to rule out the possibility of the results being driven by spurious signature exposures. We argued that spurious exposures would be sensitive to perturbations of the input mutation frequencies. We therefore performed 1000 random perturbations of the input frequencies of the 96 mutation types in each sample and computed exposures to signatures in each perturbation. Only signatures with sufficiently low variation in the exposures across perturbations were allowed for that sample. The results confirmed that the observations are signature 10 (and the other three signatures: 14, 18, and 28) is robust to perturbations, as the asymmetry remained significant in all cases.*

Page 4: Authors should explain how are the MSI and POLE samples identified. The details are missing in the methods section.

*We have clarified this in the updated manuscript.*

Page 6: "Some MMR signatures in MMR deficient patients do not significantly correlate with replication timing (sig. 15, 21, 26) or do so only in one direction of replication (such as a negative correlation in the leading direction in sig. 20), whereas others show a clear increase in the late replicated regions (sig. 6 and N4, Fig. 4-figure supplement 1)." Many MSI tumors do not have a complete impairment of MMR, and also the MMR deficiency could have been acquired late in the tumorigenic process, so that a number of mutations may have been accumulated in those tumors before the MMR deficiency. This could explain, at least partly, that there is correlation with replication timing in MSI and still be related to MMR.

*We agree that remaining but decreased correlation with replication timing in MSI samples could be in fact caused by partial impairment of MMR, or mutations acquired before the MMR impairment. We have updated the analysis for this question based on suggestions from reviewer 1. Interestingly, the updated analysis shows that four signatures have significantly steeper slopes in MSI compared to MSS samples (three with a positive slope, one with a negative slope). This observation seems difficult to explain by mutations acquired before MMR impairment or partial impairment of MMR.*

Are these signatures present in bMMRD tumors? If so, that would give a more definitive answer to the correlation of the replication timing of this signatures with MMR. If bMMRD samples do not show mutations on these signatures, at least authors show acknowledge that this is a limitation of the study that prevents a final conclusion.

*We agree that further analyses on bMMRD tumors would be very interesting in this respect. Unfortunately, we only have access to 2 WGS bMMRD tumors and are therefore underpowered for any such investigations.*

Comparing the mutation pattern in MSI and MSS tumors (Fig. 4-figure supplement 1 and Fig. 4-figure supplement 2) several signatures seem to increase their replication strand bias in MSI tumors (eg. 20 and 21). Did the authors notice that? Do they have any explanation for that?

*The suggested cause of replication strand asymmetry in mutagenesis observed in MMR-deficient samples is that MMR balances mutational asymmetries generated by DNA polymerases during replication (Haradhvala et al., 2016; Lujan et al., 2012). The trace of MMR-associated signatures in MSS samples can be due to mutations that escaped the repair by (proficient) MMR. We can speculate that after the balancing by the proficient MMR, the escaped mutations no longer exhibit the strand asymmetry. However, further research, including experimental research, is needed to validate the mechanisms of MMR balancing strand asymmetries and the related questions.*

Page 13: Methods, exclude regions: which specific mappability track (or k-mer) has been used from the UCSC genome browser?

*We have clarified this in the updated manuscript.*

Page 15: "In order to minimize the number of spurious signature exposures, the least exposed signature was incrementally removed (in both leading and lagging parts) while as long as the resulting error did not exceed the original error by 0.5%."

What is the rationale for the choice of 0.5%? Is the choice based on the ref. 69 where the authors defined a similar threshold based on randomly generated tumors?

*The value of 0.5% was chosen empirically as a tradeoff between noise removal and keeping most of the assigned signatures.*

Would be useful that the authors provide the values of 29 strand-specific signatures, and the sample-wise exposure (matching and inverse) as a supplementary table.

*We have added the strand-specific signatures as Supplementary table 3 and sample-wise exposures as Supplementary table 2.*

Also, a supplementary table with the details of the data points to reproduce figure 2 would be useful.

*We have added the data of Figure 2 as Supplementary table 4.*

In figures related to signatures (e.g., Fig 3 and 4), specify what is the scale of the y-axis and the meaning of star symbol (on column 1).

*We have clarified this in the updated manuscript.*

OTHER COMMENTS:

In discussion the authors say:

Our results also add a new perspective to the recent debate regarding the correlation of tissue-specific cell division rates with cancer risk [3]. It has been argued that this correlation is primarily attributable to "bad luck" in the form of random errors that are introduced during replication by DNA polymerases. However, the range of mutational signatures observed in cancer samples makes a purely replication-driven etiology of cancer mutations unlikely [62,63].

I do not see how the results presented in this paper do add any new perspective on this debate. The fact that "the range of mutational signatures observed in cancer samples makes a purely replication-driven etiology of cancer mutations unlikely" doesn't need the results of the present paper.

*This sentence was not meant to add a new perspective, but instead to summarize one of the previous arguments against the “bad-luck” theory. Our additions to the debate were described in the following sentences. We have expanded this part of the discussion, hopefully making the new perspective clearer.*

The scale median asymmetry in figure 2A (matching exposure - invese exposure) and in column 4 of figure 3 and 4 is misleading, as higher values don't mean higher asymmetry. Higher values can be achieved for those signatures with higher number of mutations. Would it be possible to find a value that is more meaningful of asymmetry itself, somehow normalized by the total number of mutations?

*The strand asymmetry is usually measured by two metrics: a difference between the two strands (leading – lagging), or log-transformed ratio of the two strands (log(leading/lagging)). We believe that both metrics correctly quantify size effect of the strand asymmetry, although each of them has a different interpretation. In our initial explorations, we have noticed that the second metric has a bias towards low total numbers of mutations (a similar issue in a different setting is described, e.g., in (Mariani et al., 2003)). We have therefore decided to use the first metric. Importantly, due to the properties of signtest, the choice of the metric does not affect evaluation of significance of strand asymmetry (in column 4 of Figures 3 and 4 and in y-axis of Figure 2A). In figures 3 and 4, the other columns are also not affected by the metric, as they give information about both strands separately.*

*Finally, in order to enable simple comparison of the signatures also in terms of relative values of the leading and lagging strand, we have added Fig. 2–figure supplement 1 with the second metric. We have also highlighted the difference between the two metrics in signature 17.*

The list of authors in this citation are incorrect.

62. Gao Z, Wyman MJ, Sella G, Przeworski M, Campbell P, Nik-Zainal S. Interpreting the Dependence of Mutation Rates on Age and Time. Barton NH, editor. PLOS Biol. [Internet]. Public Library of Science; 2016 [cited 2017 Mar 10];14:e1002355. Available from: http://dx.plos.org/10.1371/journal.pbio.1002355

*Thank you for noticing this error. We have corrected it in the revised manuscript.*

**Third round of review**

**Reviewer 1**

The principal concerns I have raised at each stage of this review process have still not been fully addressed. I appreciate the efforts of the authors to disentangle replication processes from repair processes but largely due to limited data they have not been able to convincingly achieve this.

As I have pointed out, there is merit in this work and I can see the benefit in its publication but in current form the conclusions are over stated - the title itself "widespread impact of DNA replication of mutational mechanisms in cancer" argues for a causal relationship between replication and the observed mutational patterns which has not been shown. Balanced reporting of these findings should:

1. Revise the title to something without causal implication, e.g. "Widespread correlation of DNA replication with mutational mechanisms in cancer".

2. The abstract should substitute "impacted by" with "correlated with" and should also raise the caveat that correlations could be be a result of biases in surveillance and repair in addition to or instead of replication.

Authors response to reviewers

*Thank you again for your consideration of our manuscript. In response to the reviewer's comments, we have modified the title and the abstract of the manuscript to reflect that our results demonstrate correlation, not causation.*
